# On stratified single-valued soft topogenous structures

**DOI:** 10.1016/j.heliyon.2024.e27926

**Published:** 2024-03-16

**Authors:** Fahad Alsharari, Yaser Saber, Hanan Alohali, Mesfer H. Alqahtani, Mubarak Ebodey, Tawfik Elmasry, Jafar Alsharif, Amal F. Soliman, Florentin Smarandache, Fahad Sikander

**Affiliations:** a Department of Mathematics, College of Science, Jouf University, Sakaka 72311, Saudi Arabia; b Department of Mathematics, College of Science Al-Zulfi, Majmaah University, P. O. Box 66, Al-Majmaah 11952, Saudi Arabia; c Department of Mathematics, Faculty of Science, Al-Azhar University, Assiut, 71524, Egypt; d Department of Mathematics, College of Science, King Saud University, P.O. Box 2455, Riyadh 11451, Saudi Arabia; e Department of Mathematics, University College of Umluj, University of Tabuk, Tabuk 48322, Saudi Arabia; f Department of Business Administration, Faculty of Science and Humanities at Hotat Sudair, Majmaah University, 11952, Riyadh, Saudi Arabia; g Department of Mathematics, College of Science and Humanities in Alkharj, Prince Sattam Bin Abdulaziz University, Alkharj, Saudi Arabia; h Department of Basic Science, Benha Faculty of Engineering, Benha University, Banha, Egypt; i Department of Mathematics, University of New Mexico, Gallup, NM 87301, USA; j Department of Basics Sciences, College of Science and Theoretical studies, Saudi Electronic University, Jeddah 23442, Saudi Arabia

**Keywords:** Stratified, Single-valued neutrosophic soft quasi-uniformity, Single-valued neutrosophic soft topogenous order, Single-valued neutrosophic soft quasi-proximity

## Abstract

This paper presents novel concepts including stratified single-valued neutrosophic soft topogenous (*stratified svns-topogenous*), stratified single-valued neutrosophic soft filter (*stratified svns-filter*), stratified single-valued neutrosophic soft quasi uniformity (*stratified svnsq-uniformity*) and stratified single-valued neutrosophic soft quasi proximity (*stratified svnsq-proximity*). Additionally, we present the idea of single-valued neutrosophic soft topogenous structures, formed by integrating svns-topogenous with svns-filter, and discuss their properties. Furthermore, we explore the connections between these single-valued neutrosophic soft topological structures and their corresponding stratifications.

## 1 Introduction and preliminaries

Various methodologies have been scrutinized for effectively handling uncertainties, encompassing fuzzy set theory [1], intuitionistic fuzzy set theory [2], vague set theory, interval mathematics [3], [4], and rough set theory [5]. However, these approaches have encountered significant challenges. Soft set theory, introduced by Molodtsov [6], has emerged as a promising alternative and has been successfully applied in diverse fields such as function smoothness [7], game theory [8], Riemann integration [9], and probability theory [10]. Notably, recent advancements in soft set theory and its applications have been particularly noteworthy in certain domains.

Fuzzy sets: Handle uncertainty by assigning degrees of membership, Intuitionistic fuzzy sets: Allow for more uncertainty with an extra degree of non-membership, Vague sets: Handle uncertainty by having a boundary region of membership, Interval mathematics: Deals with uncertainty by working with ranges of numbers, Rough sets: Handle uncertainty by approximating vague concepts, Stochastic programming: Models uncertainty through probability distributions, Robust optimization: Provides solutions that are valid under all scenarios, Simulation: Mimics real-world processes to evaluate different strategies and Decision analysis: Uses decision trees to evaluate uncertain outcomes.

Maji et al. [11], [12] provided an application for decision-making issues along with a few new definitions of soft sets. Dey and colleagues have examined the gray relational projection approach, generalized neutrosophic soft set and multiattribute decision-making in [13], [14]. The findings reported in [11], started the study linking fuzzy and soft sets were enhanced by Aktas and Caǧman [15], Feng et al. [16], Chen et al. [17], Ali et al. [18] and Sun et al. [19]. Subsequent research on the fuzzy soft sets notion was conducted by Yang et al. [20], Kharal and Ahmed [21] and Ahmed and Khara [22]. Shabir and Naz [23] distinguished a variety of issues utilizing soft sets, subsuming separation axioms. Tanay and Kandemir [24] first developed concepts of fuzzy soft topology by utilizing Chang's concept of fuzzy topology. They explored the fundamental concepts by adopting Chang [25]'s definitions, whereas Pazar Varol and Aygün [26] defined the fuzzy soft topology in Lowen's sense. Aygünoǧlu et al. [27] defined Šostak's fuzzy soft topology. Saber et al. [28] framed single-valued neutrosophic soft topological spaces $\left(¥,T^{\upsilon },T^{\mu },T^{\omega }\right)$ (*svnst-space*). In the fuzzy paradigm, there exist three alternative ways to uniformity: Kotzé's [29] uniform covering approach, Hutton's [30] uniform operator approach and Lowen's [31] entourage approach.

Smarandache originally introduced the concept of a neutrosophic set [32], which subsequently led to research on both single-valued neutrosophic sets (*svns*) and neutrosophic sets (*ns*) by Wang et al. [33] and Salama et al. [34], [35]. Various applications have been explored in the works of several researchers [36], [37], [38], [39], [40]. Saber et al. conducted detailed studies on single-valued neutrosophic regularity space (*svnr-space*), single-valued neutrosophic ideals (*svnis*), stratification of *svnt-space*, single-valued neutrosophic soft sets (*svns*), and stratified modeling in soft fuzzy topological structures in extensive works [41], [42], [43], [44], [45], [46], [47].

It is widely acknowledged that theories such as fuzzy sets, intuitionistic fuzzy sets, and rough sets are viewed as extensions of neutrosophic set theory, serving as essential mathematical tools for managing uncertainty. The concepts of stratified single-valued neutrosophic soft topogenous, which build upon the ideas introduced by Varol et al. [26], Aygünoǧlu et al. [27] and Abbas et al. [48], [49], constitute a significant contribution of this paper.

Building upon the insights gained from previous analyses, we introduce the concepts of “stratified svnsq-uniformity” (or “stratified svns-topogenous order” and “stratified svnsq-proximity”) derived from predefined “svnsq-uniformity” (or “svns-topogenous order” and “svnsq-proximity”). We investigate several properties associated with these newly formulated structures. Additionally, we explore the interrelations between these single-valued neutrosophic soft topological structures and their respective stratifications.

Throughout this study, $\overset{˜}{\left(¥,⋎\right)}$ denotes the collection of all *svns* sets on ¥, where ⋎ is the set of all parameters on ¥ and ¥ indicated to an initial universe.

The *svns* set ${£}_{⋎}$ on ¥ is said to be ι−absolute snv-soft sets and indicated by ${\overset{˜}{⋎}}^{\iota }$, if ${£}_{{}_{e}}=\overset{¯}{\iota }$ for each e∈⋎, ι∈ς, $\overset{¯}{\iota }(x)=\iota$ for every x∈¥ (where, ${\left[{\overset{˜}{⋎}}^{\iota }\right]}^{c}={\overset{˜}{⋎}}^{\iota^{c}}$, ς=[0,1]) and $\varsigma_{0}=(0,1]$.

Definition 1 [32]. Let ¥≠ϕ. A neutrosophic set (*n-set*) on X defined as

$$\Theta =\{{\langle y,\nu_{{}_{\Theta }}(y),\mu_{{}_{\Theta }}(y),\omega_{{}_{\Theta }}(y)|y\in ¥,\nu_{{}_{\Theta }}(y),\mu_{{}_{\Theta }}(y),\omega_{{}_{\Theta }}(y)\in \rfloor }^{-}0,1^{+}\lfloor \},$$

representing the degree of membership where ($\nu_{{}_{\Theta }}(y)$), the degree of indeterminacy ($\mu_{{}_{\Theta }}(y)$), and degree of nonmembership ($\omega_{{}_{\Theta }}(y)$); ∀ y∈ς to the set Θ.

Definition 2 [33]. Let ¥≠ϕ. Then *svn-set* Θ on ¥ is defined as

$$\Theta =\{\langle y,\nu_{{}_{\Theta }}(y),\mu_{{}_{\Theta }}(y),\omega_{{}_{\Theta }}(y)|y\in ¥,\nu_{{}_{\Theta }}(y),\mu_{{}_{\Theta }}(y),\omega_{{}_{\Theta }}(y)\in \varsigma \},$$

where $\nu_{{}_{\Theta }},\mu_{{}_{\Theta }},\omega_{{}_{\Theta }}:¥\to \varsigma$ and $0\leq \nu_{{}_{\Theta }}(y)+\mu_{{}_{\Theta }}(y)+\omega_{{}_{\Theta }}(y)\leq 3$.

Definition 3 [28]. $f_{{}_{A}}$ is a *svns-set* on ¥ where, $f:⋎\to \varsigma^{¥}$; i.e., $f_{e}≜f(e)$ is a *svn-set* on ¥, for all e∈A and f(e)=〈0,1,1〉, if e∉A.

The *svn-set* f(e) is termed as an element of the *svns-set* $f_{{}_{A}}$. Thus, a *svns-set* $f_{⋎}$ on ¥ can be defined as:

$$\left(f,⋎\right)=\left\{\left(e,f(e)\right)|e\in ⋎,f(e)\in \varsigma^{¥}\right\}=\left\{e,\langle \nu_{{}_{f}}(e),\mu_{{}_{f}}(e),\omega_{{}_{f}}(e)\rangle )|e\in ¥,f(e)\in \varsigma^{¥}\right\},$$

where $\nu_{{}_{f}}:⋎\to \varsigma$ ($\nu_{{}_{f}}$ is termed as a membership function), $\mu_{f}:⋎\to \varsigma$ ($\mu_{{}_{f}}$ is termed as indeterminacy function), and $\omega_{{}_{f}}:⋎\to \varsigma$ ($\omega_{{}_{f}}$ is termed as a nonmembership function) of *svns-set*.

A *svns-set* $f_{⋎}$ on ¥ is termed as a null *svns-set* (for short, Φ), if $\nu_{{}_{f}}(e)=0$, $\mu_{{}_{f}}(e)=1$ and $\omega_{{}_{f}}(e)=1$, for any e∈⋎.

A *svns-set* $f_{⋎}$ on ¥ is termed as an absolute *svns-set* (for short, $\overset{˜}{⋎}$), if $\nu_{{}_{f}}(e)=1$, $\mu_{{}_{f}}(e)=0$ and $\omega_{{}_{f}}(e)=0$, for any e∈⋎.

Definition 4 [45]$\left(¥,T^{\upsilon },T^{\mu },T^{\omega }\right)$ is a *svnst*, if $T^{\upsilon },T^{\mu },T^{\omega }:⋎\to \varsigma^{\overset{˜}{\left(¥,⋎\right)}}$ it meets the next criteria: for every e∈⋎:

$\left(T_{1}\right)$$T_{e}^{\upsilon }(\Phi )=T_{e}^{\upsilon }(\overset{˜}{⋎})=1$ and $T_{e}^{\mu }(\Phi )=T_{e}^{\mu }(\overset{˜}{⋎})=T_{e}^{\omega }(\Phi )=T_{e}^{\omega }(\overset{˜}{⋎})=0$,

$\left(T_{2}\right)$$T_{e}^{\upsilon }({£}_{\sigma }⊓\hbar_{\alpha })\geq T_{e}^{\upsilon }({£}_{\sigma })\wedge T_{e}^{\upsilon }(\hbar_{\alpha })$, $T_{e}^{\mu }({£}_{\sigma }⊓\hbar_{\alpha })\leq T_{e}^{\mu }({£}_{\sigma })\vee T_{e}^{\mu }(\hbar_{\alpha })$,

 $T_{e}^{\omega }({£}_{\sigma }⊓\hbar_{\alpha })\leq T_{e}^{\omega }({£}_{\sigma })\vee T_{e}^{\omega }(\hbar_{\alpha })$, ∀ ${£}_{\sigma },\hbar_{\alpha }\in \overset{˜}{\left(¥,⋎\right)}$,

$\left(T_{3}\right)$$T_{e}^{\upsilon }({⨆}_{j\in J}{\left[{£}_{{}_{A}}\right]}_{j})\geq {⋀}_{j\in J}T_{e}^{\upsilon }({\left[{£}_{\sigma }\right]}_{j})$, $T_{e}^{\mu }({⨆}_{j\in J}{\left[{£}_{\sigma }\right]}_{j})\leq {⋁}_{j\in J}T_{e}^{\mu }({\left[{£}_{\sigma }\right]}_{j})$,

 $T_{e}^{\omega }({⨆}_{j\in J}{\left[{£}_{\sigma }\right]}_{j})\leq {⋁}_{j\in J}T_{e}^{\omega }({\left[{£}_{\sigma }\right]}_{j})$,  ∀ ${£}_{\sigma }\in \overset{˜}{\left(¥,⋎\right)}$.

The *svnst* $\left(T^{\nu },T^{\mu },T^{\omega }\right)$ is said to be stratified If it meets the next condition

$\left(T_{s}\right)$$T_{e}^{\upsilon }({\overset{˜}{⋎}}^{\iota })=1$ and $T_{e}^{\mu }({\overset{˜}{⋎}}^{\iota })=T_{e}^{\omega }({\overset{˜}{⋎}}^{\iota })=0$ for each e∈⋎, ι∈ς.

The quadrilateral $\left(¥,T^{\nu },T^{\mu },T^{\omega }\right)$ is named *stratified svnst-space*. Representing the degree of openness $\left(T_{e}^{\upsilon }({£}_{\sigma })\right)$, the degree of indeterminacy $\left(T_{e}^{\mu }({£}_{\sigma })\right)$, and the degree of non-openness $\left(T_{e}^{\omega }({£}_{\sigma })\right)$; of a *svns* set with respect to that parameter e∈⋎.

Sometimes, we will write $T^{\upsilon \mu \omega }$ for $\left(T^{\upsilon },T^{\mu },T^{\omega }\right)$.

Now we mind some concepts and nomenclature that will be applied in this paper.

Assume $\Psi (\overset{˜}{\left(¥,⋎\right)})$ denotes the collection of all mappings $z:\overset{˜}{\left(¥,⋎\right)}\to \overset{˜}{\left(¥,⋎\right)}$ with the next properties, for each ${£}_{\sigma }\in \overset{˜}{\left(¥,⋎\right)}$

$\left(z_{1}\right)$${£}_{\sigma }=z(f_{\sigma })$,

$\left(z_{2}\right)$$z({⊔}_{j\in J}{\left({£}_{\sigma }\right)}_{j})={⊔}_{j\in J}z({\left({£}_{\sigma }\right)}_{j})$.

For $z,t\in \Psi (\overset{˜}{\left(¥,⋎\right)}$ we define that z∘t and z⊓t by

$$(z\circ t)({£}_{\sigma })=z(t({£}_{\sigma })),$$

and

$$(z⊓t)({£}_{\sigma })=⊓\{z(\hbar_{\alpha })⊔t(g_{{}_{C}})|{£}_{\sigma }=\hbar_{\alpha }⊔g_{{}_{C}}\}.$$

Let $\psi_{{}_{\varphi }}:\overset{˜}{\left(¥,⋎\right)}\to \overset{˜}{\left(U,R\right)}$ be a mapping, $z\in \Psi (\overset{˜}{\left(U,R\right)})$, then $\psi_{{}_{\varphi }}^{-1}\circ z\circ \psi_{{}_{\varphi }}\in \Psi (\overset{˜}{\left(¥,⋎\right)})$, (equivalently, $\psi_{{}_{\varphi }}^{-1}(z(\psi_{{}_{\varphi }}({£}_{\sigma })))\in \overset{˜}{\left(¥,⋎\right)}$ for any ${£}_{\sigma }\in \overset{˜}{\left(¥,⋎\right)}$.

For each $z,t,c,v\in \Psi (\overset{˜}{\left(¥,⋎\right)}$, the next characteristics hold:

(1) If $z\leq z_{1}$ and $t\leq t_{1}$, then $z⊓t⊑z_{1}⊓t_{1}$,

(2) z⊓t⊑z, z⊓t⊑t and z⊓z=z,

(3) (z⊓t)⊓c=z⊓(t⊓c),

(4) (z⊓t)∘(c⊓v)=(z∘c)⊓(t∘v).

∀, ${£}_{\sigma }\in \overset{˜}{\left(¥,⋎\right)}$, ι∈ς define the mapping $\overset{ˆ}{\iota }:\overset{˜}{\left(¥,⋎\right)}\to \overset{˜}{\left(¥,⋎\right)}$, by

$$\overset{ˆ}{\iota }({£}_{\sigma })(x)=\left\{ \begin{array}{c} {\overset{˜}{⋎}}^{sup({£}_{\sigma })},\;\;\text{if}\;\;sup({£}_{\sigma })\leq \iota ,\\ \overset{˜}{⋎},\;\;\;\;\;\;\;\;\;\;\;\;\;\text{if}\;\;\text{otherwise}, \end{array}\right.$$

where $sup({£}_{\sigma })=\underset{x\in ¥}{⋁}{£}_{{}_{e}}(x)$, ∀ e∈σ. The map $\overset{ˆ}{\iota }$ satisfy the properties ($z_{1}$) and ($z_{2}$), that is, $\overset{ˆ}{\iota }\in \Psi (\overset{˜}{\left(¥,⋎\right)})$. Furthermore, $\overset{ˆ}{\iota }$ fulfills the next properties:

Lemma 1 *Let*$\iota ,\iota_{1},\iota_{2}\in \varsigma$*:*

(1) *If* $\iota_{1}\leq \iota_{2}$*, then* ${\overset{ˆ}{\iota }}_{2}⊑{\overset{ˆ}{\iota }}_{1}$*,*

(2) $\overset{ˆ}{\iota }\circ \overset{ˆ}{\iota }=\overset{ˆ}{\iota }$ *for each* ι∈ς*,*

(3) $z⊑\overset{ˆ}{0}$ *holds for all* $z\in \Psi (\overset{˜}{\left(¥,⋎\right)})$*.*

Lemma 2 *Let*$z_{\left({£}_{\sigma }\right)}:\overset{˜}{\left(¥,⋎\right)}\to \overset{˜}{\left(¥,⋎\right)}$*be a mapping defined by*

$$z_{\left({£}_{\sigma }\right)}(\hbar_{\alpha })=\left\{ \begin{array}{c} {£}_{\sigma },\;\;\text{if}\;\;\hbar_{\alpha }⊑{£}_{\sigma },\\ \overset{˜}{⋎},\;\;\;\;\text{if}\;\;\text{otherwise}. \end{array}\right.$$

*Then,*$z_{{£}_{\sigma }}\in \Psi (\overset{˜}{\left(¥,⋎\right)})$*and*$z_{({£}_{\sigma )}}\circ z_{\left({£}_{\sigma }\right)}=z_{\left({£}_{\sigma }\right)}$*.*

Theorem 1 *Let*$\left(¥,T_{{}_{⋎}}^{\upsilon \mu \omega }\right)$*be a* svnst-space*, for every* e∈⋎*,* ${£}_{\sigma }\in \Psi (\overset{˜}{¥,⋎})$ *we define the map* ${\left(T_{st}^{\upsilon }\right)}_{e}:⋎\to \varsigma^{\overset{˜}{\left(¥,⋎\right)}}$*,* ${\left(T_{st}^{\mu }\right)}_{e}:⋎\to \varsigma^{\overset{˜}{\left(¥,⋎\right)}}$ *and* ${\left(T_{st}^{\omega }\right)}_{e}:⋎\to \varsigma^{\overset{˜}{\left(¥,⋎\right)}}$ *as follows*

$${\left(T_{st}^{\upsilon }\right)}_{e}({£}_{\sigma })=⋁\left\{\underset{j\in J}{⋀}T_{e}^{\upsilon }({\left({£}_{\sigma }\right)}_{j})|{⊔}_{j\in J}({\left({£}_{\sigma }\right)}_{j}⊓{\overset{˜}{⋎}}^{\iota_{j}})={£}_{\sigma }\right\},$$

$${\left(T_{st}^{\mu }\right)}_{e}({£}_{\sigma })=⋀\left\{\underset{j\in J}{⋁}T_{e}^{\mu }({\left({£}_{\sigma }\right)}_{j})|{⊔}_{j\in J}({\left({£}_{\sigma }\right)}_{j}⊓{\overset{˜}{⋎}}^{\iota_{j}})={£}_{\sigma }\right\},$$

$${\left(T_{st}^{\omega }\right)}_{e}({£}_{\sigma })=⋀\left\{\underset{j\in J}{⋁}T_{e}^{\omega }({\left({£}_{\sigma }\right)}_{j})|{⊔}_{j\in J}({\left({£}_{\sigma }\right)}_{j}⊓{\overset{˜}{⋎}}^{\iota_{j}})={£}_{\sigma }\right\},$$

*where* ⋁ *and* ⋀ *are taken over all families* $\left\{{\left({£}_{\sigma }\right)}_{j}:j\in J\right\}$ *with* ${£}_{\sigma }={⊔}_{j\in J}({\left({£}_{\sigma }\right)}_{j}⊓{\overset{˜}{⋎}}^{\iota_{j}})$*. Then* ${\left(T_{st}^{\upsilon \mu \omega }\right)}_{⋎}$ *is the coarsest* stratified svnst *on* ¥ *which is finer than* $T_{⋎}^{\upsilon \mu \omega }$*. And* ${\left(T_{st}^{\upsilon \mu \omega }\right)}_{⋎}$ *is called the stratification of a* svnst $T_{⋎}^{\upsilon \mu \omega }$ *on* ¥*.*

## 2 Stratified single-valued neutrosophic soft quasi-uniform spaces

Definition 5 Let $K^{\upsilon },K^{\mu },K^{\omega }:⋎\to \varsigma^{\Psi (\overset{˜}{\left(¥,⋎\right)})}$ and $\overset{˜}{e}\in ⋎$. Then $\left(K^{\upsilon },K^{\mu },K^{\omega }\right)$ is called *svnsq-uniformity* on ¥, if it satisfies these properties:

($K_{1}$) There exists $z\in \Psi (\overset{˜}{\left(¥,⋎\right)})$ such that $K_{e}^{\upsilon }(z)=1$, $K_{e}^{\mu }(z)=0$ and $K_{e}^{\omega }(z)=0$.

($K_{2}$) If $z,t\in \Psi (\overset{˜}{\left(¥,⋎\right)})$ and z⊑t, then $K_{e}^{\upsilon }(z)\leq K_{e}^{\upsilon }(t)$, $K_{e}^{\mu }(z)\geq K_{e}^{\mu }(t)$ and

 $K_{e}^{\omega }(z)\geq K_{e}^{\omega }(t)$.

($K_{3}$) For $z,t\in \Psi (\overset{˜}{\left(¥,⋎\right)})$, $K_{e}^{\upsilon }(z⊓t)\geq K_{e}^{\upsilon }(z)\wedge K_{e}^{\upsilon }(t)$, $K_{e}^{\mu }(z⊓t)\leq K_{e}^{\mu }(z)\vee K_{e}^{\mu }(t)$ and

 $K_{e}^{\omega }(z⊓t)\leq K_{e}^{\omega }(z)\vee K_{e}^{\omega }(t)$.

($K_{4}$) For $z\in \Psi (\overset{˜}{\left(¥,⋎\right)})$, $⋁\{K_{e}^{\upsilon }(z_{1})|z_{1}\circ z_{1}=z\}\geq K_{e}^{\upsilon }(z)$, $⋀\{K_{e}^{\mu }(z_{1})|z_{1}\circ z_{1}=z\}\leq$

 $K_{e}^{\mu }(z)$ and $⋀\{K_{e}^{\omega }(z_{1})|z_{1}\circ z_{1}=z\}\leq K_{e}^{\omega }(z)$.

The pair $\left(¥,K_{⋎}^{\upsilon \mu \omega }\right)$ is called a *svvsq-uniform space*.

A *svnsq-uniformity* $K_{⋎}^{\upsilon \mu \omega }$ is said to be stratified if it provides that

($K_{S}$) $K_{e}^{\upsilon }(\overset{ˆ}{\iota })=1$, $K_{e}^{\mu }(\overset{ˆ}{\iota })=0$ and $K_{e}^{\omega }(\overset{ˆ}{\iota })=0$ for any ι∈ς.

So, the pair $\left(¥,K_{{}_{\text{E}}}^{\upsilon \mu \omega }\right)$ is called a *stratified svnsq-uniform space*.

Let ${\left(K_{⋎}^{\upsilon \mu \omega }\right)}_{1}$ and ${\left(K_{⋎}^{\upsilon \mu \omega }\right)}_{2}$ be *stratified svnsq-uniformities* on ¥. We say that ${\left(K_{⋎}^{\upsilon \mu \omega }\right)}_{1}$ is finer than ${\left(K_{⋎}^{\upsilon \mu \omega }\right)}_{2}$ [${\left(K_{⋎}^{\upsilon \mu \omega }\right)}_{2}$ is coarser than ${\left(K_{⋎}^{\upsilon \mu \omega }\right)}_{1}$] denoted by ${\left(K_{⋎}^{\upsilon \mu \omega }\right)}_{2}⊑{\left(K_{⋎}^{\upsilon \mu \omega }\right)}_{1}$ if

$${\left(K_{e}^{\upsilon }\right)}_{2}(z)\leq {\left(K_{e}^{\upsilon }\right)}_{1}(z),\;\;\;{\left(K_{e}^{\mu }\right)}_{2}(z)\geq {\left(K_{e}^{\mu }\right)}_{1}(z),\;\;\;{\left(K_{e}^{\omega }\right)}_{2}(z)\geq {\left(K_{e}^{\omega }\right)}_{1}(z),$$

for any $e\in ⋎,z\in \Psi (\overset{˜}{\left(¥,⋎\right)})$.

Assume that $\left(¥,K_{⋎}^{\upsilon \mu \omega }\right)$ and $\left(U,G_{R}^{\upsilon \mu \omega }\right)$ are *svnsq-uniform spaces*. Then, $\psi_{{}_{\varphi }}:\overset{˜}{\left(¥,⋎\right)}\to \overset{˜}{\left(U,R\right)}$ is named a *svns-uniformly continuous* iff

$$G_{\varphi (e)}^{\upsilon }(t)\leq K_{e}^{\upsilon }(\psi_{{}_{\varphi }}^{-1}\circ t\circ \psi_{{}_{\varphi }}),\;\;\;\;G_{\varphi (e)}^{\mu }(t)\geq K_{e}^{\mu }(\psi_{{}_{\varphi }}^{-1}\circ t\circ \psi_{{}_{\varphi }}),$$

$$G_{\varphi (e)}^{\omega }(t)\geq K_{e}^{\omega }(\psi_{{}_{\varphi }}^{-1}\circ t\circ \psi_{{}_{\varphi }}),$$

for every $t\in \Psi (\overset{˜}{\left(\text{Y},R\right)})$, e∈⋎.

Sometimes in this paper we will use $K^{\upsilon \mu \omega }$ instead of $\left(K^{\upsilon },K^{\mu },K^{\omega }\right)$.

Theorem 2 *Assume that*$\left(¥,K_{⋎}^{\upsilon \mu \omega }\right)$*is* svnsq-uniform space *on* ¥*. Define*

$${\left(K_{st}^{\upsilon }\right)}_{e}(z)=⋁\{K_{e}^{\upsilon }(t)|t⊓\overset{ˆ}{\iota }⊑z\}for\;any\;e\in ⋎,\;\iota \in \varsigma ,\;z\in \Psi (\overset{˜}{\left(¥,⋎\right)}),$$

$${\left(K_{st}^{\mu }\right)}_{e}(z)=⋀\{K_{e}^{\mu }(t)|t⊓\overset{ˆ}{\iota }⊑z\}for\;any\;e\in ⋎,\;\iota \in \varsigma ,\;z\in \Psi (\overset{˜}{\left(¥,⋎\right)}),$$

$${\left(K_{st}^{\omega }\right)}_{e}(z)=⋀\{K_{e}^{\omega }(t)|t⊓\overset{ˆ}{\iota }⊑z\}for\;any\;e\in ⋎,\;\iota \in \varsigma ,\;z\in \Psi (\overset{˜}{\left(¥,⋎\right)}).$$

*Then* ${\left(K_{st}^{\upsilon \mu \omega }\right)}_{⋎}$ *is the coarsest* stratified svnsq-uniformity *which is finer than* $K_{⋎}^{\upsilon \mu \omega }$*.*

Proof ($K_{1}$) There exists $z\in \Psi (\overset{˜}{\left(¥,⋎\right)})$ such that $K_{e}^{\upsilon }(z)=1$, $K_{e}^{\mu }(z)=0$, $K_{e}^{\omega }(z)=0$. Since, $z=z⊓\overset{ˆ}{0}$, ${\left(K_{st}^{\upsilon }\right)}_{e}(z)=1$, ${\left(K_{st}^{\mu }\right)}_{e}(z)=0$, ${\left(K_{st}^{\omega }\right)}_{e}(z)=0$.

($K_{2}$) Obvious.

($K_{3}$) Assume that there exists $z_{1},z_{2}\in \Psi (\overset{˜}{\left(¥,⋎\right)})$ such that

$${\left(K_{st}^{\upsilon }\right)}_{e}(z_{1}⊓z_{2})≱{\left(K_{st}^{\upsilon }\right)}_{e}(z_{1})\wedge {\left(K_{st}^{\upsilon }\right)}_{e}(z_{2}),$$

$${\left(K_{st}^{\mu }\right)}_{e}(z_{1}⊓z_{2})≰{\left(K_{st}^{\mu }\right)}_{e}(z_{1})\vee {\left(K_{st}^{\mu }\right)}_{e}(z_{2}),$$

$${\left(K_{st}^{\omega }\right)}_{e}(z_{1}⊓z_{2})≰{\left(K_{st}^{\omega }\right)}_{e}(z_{1})\vee {\left(K_{st}^{\omega }\right)}_{e}(z_{2}).$$

From the concept of ${\left(K_{st}^{\upsilon \mu \omega }\right)}_{⋎}$ there are $t_{1},t_{2}\in \Psi (\overset{˜}{\left(¥,⋎\right)})$, $\iota_{1},\iota_{2}\in \varsigma$ with $z_{1}⊒t_{1}⊓\overset{ˆ}{\iota_{1}}$ and $z_{2}⊒t_{2}⊓\overset{ˆ}{\iota_{2}}$ such that

$${\left(K_{st}^{\upsilon }\right)}_{e}(z_{1}⊓z_{2})≱K_{e}^{\upsilon }(t_{1})\wedge K_{e}^{\upsilon }(t_{2}),$$

(1) $${\left(K_{st}^{\mu }\right)}_{e}(z_{1}⊓z_{2})≰K_{e}^{\mu }(t_{1})\vee K_{e}^{\mu }(t_{2}),$$

$${\left(K_{st}^{\omega }\right)}_{e}(z_{1}⊓z_{2})≰K_{e}^{\omega }(t_{1})\vee K_{e}^{\omega }(t_{2}).$$

On another side, $\left(z_{1}⊓z_{2})⊒(t_{1}⊓t_{2})⊓(\overset{ˆ}{\iota_{1}}⊓\overset{ˆ}{\iota_{2}}\right)$. By Lemma 1(3), we obtain $(\overset{ˆ}{\iota_{1}}⊓\overset{ˆ}{\iota_{2}})=\overset{ˆ}{\iota_{1}}$ or $\overset{ˆ}{\iota_{2}}$, then

$${\left(K_{st}^{\upsilon }\right)}_{e}(z_{1}⊓z_{2})\geq K_{e}^{\upsilon }(t_{1}⊓t_{2})\geq K_{e}^{\upsilon }(t_{1})\wedge K_{e}^{\upsilon }(t_{2}),$$

$${\left(K_{st}^{\mu }\right)}_{e}(z_{1}⊓z_{2})\leq K_{e}^{\mu }(t_{1}⊓t_{2})\leq K_{e}^{\mu }(t_{1})\vee K_{e}^{\mu }(t_{2}),$$

$${\left(K_{st}^{\omega }\right)}_{e}(z_{1}⊓z_{2})\leq K_{e}^{\omega }(t_{1}⊓t_{2})\leq K_{e}^{\omega }(t_{1})\vee K_{e}^{\omega }(t_{2}).$$

It is a contradiction for equation (1). Hence ($K_{3}$) holds.

($K_{4}$) Let $z\in \Psi (\overset{˜}{\left(¥,⋎\right)})$, be a given such that

$${\left(K_{st}^{\upsilon }\right)}_{e}(z)≰⋁\{{\left(K_{st}^{\upsilon }\right)}_{e}(z_{1})|z⊒z_{1}\circ z_{1}\},$$

$${\left(K_{st}^{\mu }\right)}_{e}(z)≱⋀\{{\left(K_{st}^{\mu }\right)}_{e}(z_{1})|z⊒z_{1}\circ z_{1}\},$$

$${\left(K_{st}^{\omega }\right)}_{e}(z)≱⋀\{{\left(K_{st}^{\omega }\right)}_{e}(z_{1})|z⊒z_{1}\circ z_{1}\}.$$

From the concept of $\left[{\left(K_{st}^{\upsilon }\right)}_{e}(z),{\left(K_{st}^{\mu }\right)}_{e}(z\right)$, ${\left(K_{st}^{\omega }\right)}_{e}(z)$] there exist $t\in \Psi (\overset{˜}{\left(¥,⋎\right)})$, ι∈ς with $z⊒t⊓\overset{ˆ}{\iota }$ such that

$$K_{e}^{\upsilon }(t)≰\{{\left(K_{st}^{\upsilon }\right)}_{e}(z_{1})|z⊒z_{1}\circ z_{1}\},$$

$$K_{e}^{\mu }(t)≱\{{\left(K_{st}^{\mu }\right)}_{e}(z_{1})|z⊒z_{1}\circ z_{1}\},$$

$$K_{e}^{\omega }(t)≱\{{\left(K_{st}^{\omega }\right)}_{e}(z_{1})|z⊒z_{1}\circ z_{1}\}.$$

Since $K_{⋎}^{\upsilon \mu \omega }$ is *svnsq-uniformity* on ¥,

$$⋁\{K_{e}^{\upsilon }(c)|t⊒c\circ c\}\geq K_{e}^{\upsilon }(t),$$

$$⋀\{K_{e}^{\mu }(c)|t⊒c\circ c\}\leq K_{e}^{\mu }(t),$$

$$⋀\{K_{e}^{\omega }(c)|t⊒c\circ c\}\leq K_{e}^{\omega }(t).$$

There exist $c\in \Psi (\overset{˜}{\left(¥,⋎\right)})$ with c∘c⊑t such that

$$K_{e}^{\upsilon }(c)≰⋁\{{\left(K_{st}^{\upsilon }\right)}_{e}(z_{1})|z⊒z_{1}\circ z_{1}\},$$

(2) $$K_{e}^{\mu }(c)≱⋀\{{\left(K_{st}^{\mu }\right)}_{e}(z_{1})|z⊒z_{1}\circ z_{1}\},$$

$$K_{e}^{\omega }(c)≱⋀\{{\left(K_{st}^{\omega }\right)}_{e}(z_{1})|z⊒z_{1}\circ z_{1}.$$

On another side,

$$(c⊓\overset{ˆ}{\iota })\circ (c⊓\overset{ˆ}{\iota })⊑(c\circ c)⊓\overset{ˆ}{\iota }⊑t⊓\overset{ˆ}{\iota }⊑z,$$

that is, $c⊓\overset{ˆ}{\iota }=z_{1}$ with $z=z_{1}\circ z_{1}$,

$$⋁\{{\left(K_{st}^{\upsilon }\right)}_{e}(z_{1})|z⊒z_{1}\circ z_{1}\geq {\left(K_{st}^{\upsilon }\right)}_{e}(z_{1})\geq K_{e}^{\upsilon }(c),$$

$$⋀\{{\left(K_{st}^{\mu }\right)}_{e}(z_{1})|z⊒z_{1}\circ z_{1}\}\leq {\left(K_{st}^{\mu }\right)}_{e}(z_{1})\leq K_{e}^{\mu }(c),$$

$$⋀\{{\left(K_{st}^{\omega }\right)}_{e}(z_{1})|z⊒z_{1}\circ z_{1}\}\leq {\left(K_{st}^{\omega }\right)}_{e}(z_{1})\leq K_{e}^{\omega }(c).$$

In this case, it is a contradiction with the hypothesis as we can see from Equations (2). Hence ($K_{4}$) holds.

($K_{S}$) By Lemma 1(3), we have $z⊑\overset{ˆ}{0}$ holds for every $z\in \Psi (\overset{˜}{\left(¥,⋎\right)})$. Since ${¥}_{⋎}^{\upsilon \mu \omega }$ satisfies the conditions $K_{1}$ and $K_{2}$, we obtain $K_{e}^{\upsilon }(\overset{ˆ}{0})=1$, $K_{e}^{\mu }(\overset{ˆ}{0})=0$ and $K_{e}^{\omega }(\overset{ˆ}{0})=0$. So, $\overset{ˆ}{0}⊓\overset{ˆ}{\iota }=\overset{ˆ}{\iota }$ for every ι∈ς, then ${\left(K_{st}^{\upsilon }\right)}_{e}(\overset{ˆ}{\iota })=1$, ${\left(K_{st}^{\mu }\right)}_{e}(\overset{ˆ}{\iota })=0$, ${\left(K_{st}^{\omega }\right)}_{e}(\overset{ˆ}{\iota })=0$. Hence, ${\left(K_{st}^{\upsilon \mu \omega }\right)}_{⋎}$ is stratified.

For $t=t⊓\overset{ˆ}{0}$ and $t\in \Psi (\overset{˜}{\left(¥,⋎\right)})$. Then, ${\left(K_{st}^{\upsilon }\right)}_{e}(t)\geq K_{e}^{\upsilon }(t)$, ${\left(K_{st}^{\mu }\right)}_{e}(t)\leq K_{e}^{\mu }(t)$ and ${\left(K_{st}^{\omega }\right)}_{e}(t)\leq K_{e}^{\omega }(t)$. Hence, ${\left(K_{st}^{\upsilon \mu \omega }\right)}_{⋎}$ is finer than $K_{⋎}^{\upsilon \mu \omega }$.

Finally, let $G_{⋎}^{\upsilon \mu \omega }$ be *stratified svnsq-uniformity* finer than $K_{⋎}^{\upsilon \mu \omega }$.

Presume that there exists $z\in \Psi (\overset{˜}{\left(¥,⋎\right)})$ s.t.,

$$G_{e}^{\upsilon }(z)≱{\left(K_{st}^{\upsilon }\right)}_{e}(z),\;\;G_{e}^{\mu }(z)≰{\left(K_{st}^{\mu }\right)}_{e}(z),\;\;G_{e}^{\omega }(z)≰{\left(K_{st}^{\omega }\right)}_{e}(z).$$

From the concept of [${{{K_{st}^{\upsilon })}_{e}(z),K_{st}^{\mu })}_{e}(z),K_{st}^{\omega })}_{e}(z)$]), there exist $t\in \Psi (\overset{˜}{\left(¥,⋎\right)})$, ι∈ς with $z⊒t⊓\overset{ˆ}{\iota }$ such that

(3) $$G_{e}^{\upsilon }(z)≱K_{e}^{\upsilon }(t),\;\;G_{e}^{\mu }(z)≰K_{e}^{\mu }(t),\;\;G_{e}^{\omega }(z)≰K_{e}^{\omega }(t).$$

Since $G_{⋎}^{\upsilon \mu \omega }$ be *stratified svnsq-uniformity*,

$$G_{e}^{\upsilon }(z)\geq G_{e}^{\upsilon }(t⊓\overset{ˆ}{\iota })\geq G_{e}^{\upsilon }(t)\wedge G_{e}^{\upsilon }(\overset{ˆ}{\iota }))\geq G_{e}^{\upsilon }(t)\geq K_{e}^{\upsilon }(t),$$

$$G_{e}^{\mu }(z)\leq G_{e}^{\upsilon }(t⊓\overset{ˆ}{\iota })\leq G_{e}^{\mu }(t)\wedge G_{e}^{\upsilon }(\overset{ˆ}{\iota }))\leq G_{e}^{\mu }(t)\leq K_{e}^{\mu }(t),$$

$$G_{e}^{\mu }(z)\leq G_{e}^{\omega }(t⊓\overset{ˆ}{\iota })\leq G_{e}^{\omega }(t)\wedge G_{e}^{\omega }(\overset{ˆ}{\iota }))\leq G_{e}^{\omega }(t)\leq K_{e}^{\omega }(t).$$

In this case, the hypothesis is contradicted, as we see in Equations (3). Therefore, ${\left(K_{st}^{\upsilon \mu \omega }\right)}_{⋎}$ is the coarsest *stratified svnsq-uniformity* which is finer than $K_{⋎}^{\upsilon \mu \omega }$. □

Theorem 3 *Let*$\left(¥,K_{⋎}^{\upsilon \mu \omega }\right)$*and*$\left(U,G_{R}^{\upsilon \mu \omega }\right)$*be two* svnsq-uniform spaces*. If the mapping* $\psi_{{}_{\varphi }}:(¥,K_{⋎}^{\upsilon \mu \omega })\to (U,G_{R}^{\upsilon \mu \omega })$ *is a* svns-uniformly continuous*, then the mapping* $\psi_{{}_{\varphi }}:(¥,{\left(K_{st}^{\upsilon \mu \omega }\right)}_{⋎})\to (U,{\left(G_{st}^{\upsilon \mu \omega }\right)}_{R})$ *is a* svns-uniformly continuous*, where* ${\left(K_{st}^{\upsilon \mu \omega }\right)}_{⋎}$ *and* ${\left(G_{st}^{\upsilon \mu \omega }\right)}_{⋎}$ *are the stratification of* $K_{⋎}^{\upsilon \mu \omega }$ *and* $G_{⋎}^{\upsilon \mu \omega }$*, respectively.*

Proof We will prove that

$${\left(K_{st}^{\upsilon }\right)}_{e}(\psi_{{}_{\varphi }}^{-1}\circ t\circ \psi_{{}_{\varphi }})\geq {\left(G_{st}^{\upsilon }\right)}_{\varphi (e)}(t),$$

$${\left(K_{st}^{\mu }\right)}_{e}(\psi_{{}_{\varphi }}^{-1}\circ t\circ \psi_{{}_{\varphi }})\leq {\left(G_{st}^{\mu }\right)}_{\varphi (e)}(t),$$

$${\left(K_{st}^{\omega }\right)}_{e}(\psi_{{}_{\varphi }}^{-1}\circ t\circ \psi_{{}_{\varphi }})\leq {\left(G_{st}^{\omega }\right)}_{\varphi (e)}(t),$$

for each $t\in \Psi (\overset{˜}{\left(U,R\right)})$. Assume that

$${\left(K_{st}^{\upsilon }\right)}_{e}(\psi_{{}_{\varphi }}^{-1}\circ t\circ \psi_{{}_{\varphi }})≱{\left(G_{st}^{\upsilon }\right)}_{\varphi (e)}(t),\;\;\;\;{\left(K_{st}^{\mu }\right)}_{e}(\psi_{{}_{\varphi }}^{-1}\circ t\circ \psi_{{}_{\varphi }})≰{\left(G_{st}^{\mu }\right)}_{\varphi (e)}(t),$$

$${\left(K_{st}^{\omega }\right)}_{e}(\psi_{{}_{\varphi }}^{-1}\circ t\circ \psi_{{}_{\varphi }})≰{\left(G_{st}^{\omega }\right)}_{\varphi (e)}(t).$$

From the concept of [${\left(G_{st}^{\upsilon }\right)}_{\varphi (e)}(t),{\left(G_{st}^{\mu }\right)}_{\varphi (e)}(t),{\left(G_{st}^{\omega }\right)}_{\varphi (e))}(t)$]), there exist $c\in \Psi (\overset{˜}{\left(U,R\right)})$, ι∈ς with $t⊒c⊓\overset{ˆ}{\iota }$ such that

$${\left(K_{st}^{\upsilon }\right)}_{e}(\psi_{{}_{\varphi }}^{-1}\circ t\circ \psi_{{}_{\varphi }})≱G_{\varphi (e)}^{\upsilon }(c),$$

(4) $${\left(K_{st}^{\mu }\right)}_{e}(\psi_{{}_{\varphi }}^{-1}\circ t\circ \psi_{{}_{\varphi }})≰G_{\varphi (e)}^{\mu }(c),$$

$${\left(K_{st}^{\omega }\right)}_{e}(\psi_{{}_{\varphi }}^{-1}\circ t\circ \psi_{{}_{\varphi }})≰G_{\varphi (e)}^{\omega }(c).$$

Since $\psi_{{}_{\varphi }}:(¥,K_{⋎}^{\upsilon \mu \omega })\to (U,G_{R}^{\upsilon \mu \omega })$ is a *svns-uniformly continuous*,

$$K_{e}^{\upsilon }(\psi_{{}_{\varphi }}^{-1}\circ c\circ \psi_{{}_{\varphi }})\geq G_{\varphi (e)}^{\upsilon }(c),\;\;\;\;K_{e}^{\mu }(\psi_{{}_{\varphi }}^{-1}\circ c\circ \psi_{{}_{\varphi }})\leq G_{\varphi (e)}^{\mu }(c),$$

$$K_{e}^{\omega }(\psi_{{}_{\varphi }}^{-1}\circ c\circ \psi_{{}_{\varphi }})\leq G_{\varphi (e)}^{\omega }(c).$$

From the concept of [${\left(K_{st}^{\upsilon }\right)}_{e}(\psi_{{}_{\varphi }}^{-1}\circ t\circ \psi_{{}_{\varphi }}),{\left(K_{st}^{\mu }\right)}_{e}(\psi_{{}_{\varphi }}^{-1}\circ t\circ \psi_{{}_{\varphi }}),{\left(K_{st}^{\omega }\right)}_{e}(\psi_{{}_{\varphi }}^{-1}\circ t\circ \psi_{{}_{\varphi }})$] we obtain

$${\left(K_{st}^{\upsilon }\right)}_{e}(\psi_{{}_{\varphi }}^{-1}\circ t\circ \psi_{{}_{\varphi }})\geq K_{e}^{\upsilon }(\psi_{{}_{\varphi }}^{-1}\circ c\circ \psi_{{}_{\varphi }})\geq G_{\varphi (e)}^{\upsilon }(c),$$

$${\left(K_{st}^{\mu }\right)}_{e}(\psi_{{}_{\varphi }}^{-1}\circ t\circ \psi_{{}_{\varphi }})\leq K_{e}^{\mu }(\psi_{{}_{\varphi }}^{-1}\circ c\circ \psi_{{}_{\varphi }})\leq G_{\varphi (e)}^{\mu }(c),$$

$${\left(K_{st}^{\omega }\right)}_{e}(\psi_{{}_{\varphi }}^{-1}\circ t\circ \psi_{{}_{\varphi }})\leq K_{e}^{\omega }(\psi_{{}_{\varphi }}^{-1}\circ c\circ \psi_{{}_{\varphi }})\leq G_{\varphi (e)}^{\omega }(c).$$

In this case, the hypothesis is contradicted as we can see from Equations (4). □

Theorem 4 *Let*$\left(¥,T_{{}_{⋎}}^{\upsilon \mu \omega }\right)$*be a* stratified svnsts*. Define,*

$$K_{e}^{\upsilon }(z)=⋁\left\{\underset{j\in J}{⋀}T_{e}^{\upsilon }({\left({£}_{\sigma }\right)}_{j})|\;\forall \;z\in \Psi (\overset{˜}{\left(¥,⋎\right)}),\;{⊓}_{j\in J}z_{{\left({£}_{\sigma }\right)}_{j}}⊑z\right\},$$

$$K_{e}^{\mu }(z)=⋀\left\{\underset{j\in J}{⋁}T_{e}^{\mu }({\left({£}_{\sigma }\right)}_{j})|\;\forall \;z\in \Psi (\overset{˜}{\left(¥,⋎\right)}),\;{⊓}_{j\in J}z_{{\left({£}_{\sigma }\right)}_{j}}⊑z\right\},$$

$$K_{e}^{\omega }(z)=⋀\left\{\underset{j\in J}{⋁}T_{e}^{\omega }({\left({£}_{\sigma }\right)}_{j})|\;\forall \;z\in \Psi (\overset{˜}{\left(¥,⋎\right)}),\;{⊓}_{j\in J}z_{{\left({£}_{\sigma }\right)}_{j}}⊑z\right\},$$

*where* ⋁ *and* ⋀ *are possessed over all collections* $\left\{{\left({£}_{\sigma }\right)}_{j}|{⊓}_{j\in J}z_{{\left({£}_{\sigma }\right)}_{j}}⊑z\right\}$*. Then* $K_{⋎}^{\upsilon \mu \omega }$ *is* stratified svnsq-uniformity *on* ¥*.*

Proof ($K_{1}$) and ($K_{2}$) straightforward.

($K_{3}$) Assume there exists $z,t\in \Psi (\overset{˜}{\left(¥,⋎\right)})$, such that $K_{e}^{\upsilon }(z⊓t)≱K_{e}^{\upsilon }(z)\wedge K_{e}^{\upsilon }(t),\;\;\;K_{e}^{\mu }(z⊓t)≰K_{e}^{\upsilon }(z)\vee K_{e}^{\mu }(t)$, and $K_{e}^{\omega }(z⊓t)≰K_{e}^{\omega }(z)\vee K_{e}^{\omega }(t)$. Then, by the concept of $K_{⋎}^{\upsilon \mu \omega }$, there exist two finite collections $\left\{{\left(\hbar_{\alpha }\right)}_{i}|t⊒{⊓}_{i\in \Gamma }t_{{\left(\hbar_{\alpha }\right)}_{i}}\right\}$ and $\left\{{\left({£}_{\sigma }\right)}_{j}|z⊒{⊓}_{j\in J}z_{{\left({£}_{\sigma }\right)}_{j}}\right\}$ such that

$$K_{e}^{\upsilon }(z⊓t)≱\left[\underset{j\in J}{⋀}T_{e}^{\upsilon }({\left({£}_{\sigma }\right)}_{j})\right]\wedge \left[\underset{i\in \Gamma }{⋀}T_{e}^{\upsilon }({\left(\hbar_{\alpha }\right)}_{i})\right],$$

(5) $$K_{e}^{\mu }(z⊓t)≰\left[\underset{j\in J}{⋁}T_{e}^{\mu }({\left({£}_{\sigma }\right)}_{j})\right]\vee \left[\underset{i\in \Gamma }{⋁}T_{e}^{\mu }({\left(\hbar_{\alpha }\right)}_{i})\right],$$

$$K_{e}^{\omega }(z⊓t)≰\left[\underset{j\in J}{⋁}T_{e}^{\omega }({\left({£}_{\sigma }\right)}_{j})\right]\vee \left[\underset{i\in \Gamma }{⋁}T_{e}^{\omega }({\left(\hbar_{\alpha }\right)}_{i})\right].$$

On another side,

$$z⊓t⊒({⊓}_{j\in J}z_{{\left({£}_{\sigma }\right)}_{j}})\wedge ({⊓}_{i\in \Gamma }t_{{\left(\hbar_{\alpha }\right)}_{i}})⊒{⊓}_{j\in J,i\in \Gamma }{\left(z⊓t\right)}_{{\left({£}_{\sigma }\right)}_{j}⊓{\left(\hbar_{\alpha }\right)}_{i}}.$$

Then by the definition of $K_{⋎}^{\upsilon \mu \omega }$,

$$K_{e}^{\upsilon }(z⊓t)\geq \underset{j\in J,i\in \Gamma }{⋀}T_{e}^{\upsilon }({\left({£}_{\sigma }\right)}_{j}⊓{\left(\hbar_{\alpha }\right)}_{i})\geq \underset{j\in J,i\in \Gamma }{⋀}(T_{e}^{\upsilon }({\left({£}_{\sigma }\right)}_{j})⊓T_{e}^{\upsilon }({\left(\hbar_{\alpha }\right)}_{i}))\geq \left[\underset{j\in J}{⋀}T_{e}^{\upsilon }({\left({£}_{\sigma }\right)}_{j})\right]\wedge \left[\underset{i\in \Gamma }{⋀}T_{e}^{\upsilon }({\left(\hbar_{\alpha }\right)}_{i})\right],$$

$$K_{e}^{\mu }(z⊓t)\leq \underset{j\in J,i\in \Gamma }{⋁}T_{e}^{\mu }({\left({£}_{\sigma }\right)}_{j}⊓{\left(\hbar_{\alpha }\right)}_{i})\leq \underset{j\in J,i\in \Gamma }{⋁}(T_{e}^{\mu }({\left({£}_{\sigma }\right)}_{j})⊔T_{e}^{\mu }({\left(\hbar_{\alpha }\right)}_{i}))\leq \left[\underset{j\in J}{⋁}T_{e}^{\mu }({\left({£}_{\sigma }\right)}_{j})\right]\vee \left[\underset{i\in \Gamma }{⋁}T_{e}^{\mu }({\left(\hbar_{\alpha }\right)}_{i})\right],$$

$$K_{e}^{\omega }(z⊓t)\leq \underset{j\in J,i\in \Gamma }{⋁}T_{e}^{\omega }({\left({£}_{\sigma }\right)}_{j}⊓{\left(\hbar_{\alpha }\right)}_{i})\leq \underset{j\in J,i\in \Gamma }{⋁}(T_{e}^{\omega }({\left({£}_{\sigma }\right)}_{j})⊓T_{e}^{\omega }{\left(\hbar_{\alpha }\right)}_{i}))\leq \left[\underset{j\in J}{⋁}T_{e}^{\omega }({\left({£}_{\sigma }\right)}_{j})\right]\vee \left[\underset{i\in \Gamma }{⋁}T_{e}^{\omega }({\left(\hbar_{\alpha }\right)}_{i})\right],$$

which is a contradiction for equations (5), and then $\left(K_{3}\right)$ holds.

($K_{4}$) Since, $z_{{}_{{£}_{\sigma }}}\circ z_{{}_{{£}_{\sigma }}}=z_{{}_{{£}_{\sigma }}}$. From the Lemma 2, then, ($K_{4}$) holds.

($K_{1}$) and ($K_{S}$) There exists $z=z_{{\overset{˜}{⋎}}^{\iota }}=\overset{ˆ}{\iota }$, then $K_{e}^{\upsilon }(\overset{ˆ}{\iota })\geq T_{e}^{\upsilon }({\overset{˜}{⋎}}^{\iota })=1$, $K_{e}^{\mu }(\overset{ˆ}{\iota })\leq T_{e}^{\mu }({\overset{˜}{⋎}}^{\iota })=0$ and $K_{e}^{\omega }(\overset{ˆ}{\iota })\leq T_{e}^{\omega }({\overset{˜}{⋎}}^{\iota })=0$. Therefore, $K_{e}^{\upsilon }(\overset{ˆ}{\iota })=1$, $K_{e}^{\mu }(\overset{ˆ}{\iota })=0$ and $K_{e}^{\omega }(\overset{ˆ}{\iota })=0$ for each ι∈ς. Hence, $K_{⋎}^{\upsilon \mu \omega }$ is stratified. □

## 3 Stratified single-valued neutrosophic soft topogenous order spaces

Definition 6 Maps $H^{\upsilon }:⋎\to \varsigma^{\overset{˜}{\left(¥,⋎\right)}\times \overset{˜}{\left(¥,⋎\right)}}$, $H^{\mu }:⋎\to \varsigma^{\overset{˜}{\left(¥,⋎\right)}\times \overset{˜}{\left(¥,⋎\right)}}$ and $H^{\omega }:⋎\to \varsigma^{\overset{˜}{\left(¥,⋎\right)}\times \overset{˜}{\left(¥,⋎\right)}}$ are said to be *svns-topogenous order* on ¥ if it fulfills the next properties: ∀ e∈⋎ and ${£}_{\sigma },\hbar_{\alpha }\in \overset{˜}{\left(¥,⋎\right)}$,

($H_{1}$) $H_{e}^{\mu }(\overset{˜}{⋎},\overset{˜}{⋎})=H_{e}^{\mu }(\Phi ,\Phi )=H_{e}^{\omega }(\overset{˜}{⋎},\overset{˜}{⋎})=H_{e}^{\omega }(\Phi ,\Phi )=0$, $H_{e}^{\upsilon }(\overset{˜}{⋎},\overset{˜}{⋎})=H_{e}^{\upsilon }(\Phi ,\Phi )=1$,

($H_{2}$) If $H_{e}^{\upsilon }({£}_{\sigma },\hbar_{\alpha })\neq 0$, $H_{e}^{\mu }({£}_{\sigma },\hbar_{\alpha })\neq 1$ and $H_{e}^{\omega }({£}_{\sigma },\hbar_{\alpha })\neq 1$, then ${£}_{\sigma }⊑\hbar_{\alpha }$.

($H_{3}$) If ${£}_{\sigma }⊑{\left({£}_{\sigma }\right)}_{1}$, ${\left(\hbar_{\alpha }\right)}_{1}⊑\hbar_{\alpha }$, then $H_{e}^{\upsilon }({\left({£}_{\sigma }\right)}_{1},{\left(\hbar_{\alpha }\right)}_{1})\leq H_{e}^{\upsilon }({£}_{\sigma },\hbar_{\alpha })$,

 $H_{e}^{\mu }({\left({£}_{\sigma }\right)}_{1},{\left(\hbar_{\alpha }\right)}_{1})\geq H_{e}^{\mu }({£}_{\sigma },\hbar_{\alpha })$ and $H_{e}^{\omega }({\left({£}_{\sigma }\right)}_{1},{\left(\hbar_{\alpha }\right)}_{1})\geq H_{e}^{\omega }({£}_{\sigma },\hbar_{\alpha })$.

($H_{4}$) (i)

$$H_{e}^{\upsilon }({\left({£}_{\sigma }\right)}_{1}⊔{\left({£}_{\sigma }\right)}_{2},{\left(\hbar_{\alpha }\right)}_{1}⊔{\left(\hbar_{\alpha }\right)}_{2})\geq H_{e}^{\upsilon }({\left({£}_{\sigma }\right)}_{1},{\left(\hbar_{\alpha }\right)}_{1})\wedge H_{e}^{\upsilon }({\left({£}_{\sigma }\right)}_{2},{\left(\hbar_{\alpha }\right)}_{2}),$$

$$H_{e}^{\mu }({\left({£}_{\sigma }\right)}_{1}⊔{\left({£}_{\sigma }\right)}_{2},{\left(\hbar_{\alpha }\right)}_{1}⊔{\left(\hbar_{\alpha }\right)}_{2})\leq H_{e}^{\mu }({\left({£}_{\sigma }\right)}_{1},{\left(\hbar_{\alpha }\right)}_{1})\vee H_{e}^{\mu }({\left({£}_{\sigma }\right)}_{2},{\left(\hbar_{\alpha }\right)}_{2}),$$

$$H_{e}^{\omega }({\left({£}_{\sigma }\right)}_{1}⊔{\left({£}_{\sigma }\right)}_{2},{\left(\hbar_{\alpha }\right)}_{1}⊔{\left(\hbar_{\alpha }\right)}_{2})\leq H_{e}^{\omega }({\left({£}_{\sigma }\right)}_{1},{\left(\hbar_{\alpha }\right)}_{1})\vee H_{e}^{\omega }({\left({£}_{\sigma }\right)}_{2},{\left(\hbar_{\alpha }\right)}_{2}).$$

 (ii)

$$H_{e}^{\upsilon }{\left({\left({£}_{\sigma }\right)}_{1}⊓{\left({£}_{\sigma }\right)}_{2},{\left(\hbar_{\alpha }\right)}_{1}⊓\hbar_{\alpha }\right)}_{2})\geq H_{e}^{\upsilon }({\left({£}_{\sigma }\right)}_{1},{\left(\hbar_{\alpha }\right)}_{1})\wedge H_{e}^{\upsilon }({\left({£}_{\sigma }\right)}_{2},{\left(\hbar_{\alpha }\right)}_{2}),$$

$$H_{e}^{\upsilon }{\left({\left({£}_{\sigma }\right)}_{1}⊓{\left({£}_{\sigma }\right)}_{2},{\left(\hbar_{\alpha }\right)}_{1}⊓\hbar_{\alpha }\right)}_{2})\leq H_{e}^{\upsilon }({\left({£}_{\sigma }\right)}_{1},{\left(\hbar_{\alpha }\right)}_{1})\vee H_{e}^{\upsilon }({\left({£}_{\sigma }\right)}_{2},{\left(\hbar_{\alpha }\right)}_{2}).$$

$$H_{e}^{\omega }({\left({£}_{\sigma }\right)}_{1}⊓{\left({£}_{\sigma }\right)}_{2},{\left(\hbar_{\sigma }\right)}_{1}⊓{\left(\hbar_{\sigma }\right)}_{2})\leq H_{e}^{\omega }({\left({£}_{\sigma }\right)}_{1},{\left(\hbar_{\alpha }\right)}_{1})\vee H_{e}^{\omega }({\left({£}_{\sigma }\right)}_{2},{\left(\hbar_{\alpha }\right)}_{2}).$$

Therefore, ($¥,H_{⋎}^{\upsilon \mu \omega }$) is termed to be a *svns-topogenous order space*. Also, $H_{¥}^{\upsilon \mu \omega }$ is said to be

(1) Symmetrical iff $H_{e}^{\upsilon }({£}_{\sigma },\hbar_{\alpha })=H_{e}^{\upsilon }(\hbar_{\alpha }^{c},{£}_{\sigma }^{c})$, $H_{e}^{\mu }({£}_{\sigma },\hbar_{\alpha })=H_{e}^{\mu }(\hbar_{\alpha }^{c},{£}_{\sigma }^{c})$ and $H_{e}^{\omega }(\hbar_{\alpha },{£}_{\sigma })=H_{e}^{\omega }(\hbar_{\alpha }^{c},{£}_{\sigma }^{c})$.

(2) Perfect iff

$$H_{e}^{\upsilon }({⊔}_{j\in J}{\left({£}_{\sigma }\right)}_{j},{⊔}_{j\in J}{\left(\hbar_{\alpha }\right)}_{j})\geq \underset{j\in J}{⋀}H_{e}^{\upsilon }({\left({£}_{\sigma }\right)}_{j},{\left(\hbar_{\alpha }\right)}_{j}),$$

$$H_{e}^{\mu }{\left({⊔}_{j\in J}{\left({£}_{\sigma }\right)}_{j},{⊔}_{j\in J}(\hbar_{\alpha })\right)}_{j}\leq \underset{j\in J}{⋁}H_{e}^{\mu }({\left({£}_{\sigma }\right)}_{j},{\left(\hbar_{\alpha }\right)}_{j}),$$

$$H_{e}^{\omega }{\left({⊔}_{j\in J}{\left({£}_{\sigma }\right)}_{j},{⊔}_{j\in J}(\hbar_{\alpha })\right)}_{j}\leq \underset{j\in J}{⋁}H_{e}^{\omega }({\left({£}_{\sigma }\right)}_{j},{\left(\hbar_{\alpha }\right)}_{j}).$$

(3) Stratified iff $H_{⋎}^{\upsilon \mu \omega }$ satisfies the condition:

($H_{S}$) For every ι∈ς, $H_{e}^{\upsilon }({\overset{˜}{⋎}}^{\iota },{\overset{˜}{⋎}}^{\iota })=1$, $H_{e}^{\mu }({\overset{˜}{⋎}}^{\iota },{\overset{˜}{⋎}}^{\iota })=0$, $H_{e}^{\omega }({\overset{˜}{⋎}}^{\iota },{\overset{˜}{⋎}}^{\iota })=0$.

Suppose that ${\left(H_{⋎}^{\upsilon \mu \omega }\right)}_{1}$ and ${\left(H_{⋎}^{\upsilon \mu \omega }\right)}_{2}$ be *svns-topogenous order space* on ⋎. In our opinion ${\left(H_{⋎}^{\upsilon \mu \omega }\right)}_{1}$ is finer than ${\left(H_{⋎}^{\upsilon \mu \omega }\right)}_{2}$ $\left[{\left(H_{⋎}^{\upsilon \mu \omega }\right)}_{2}\;is\;coarser\;than\;{\left(H_{⋎}^{\upsilon \mu \omega }\right)}_{1}\right]$ indicated by ${\left(H_{⋎}^{\upsilon \mu \omega }\right)}_{1}⊒{\left(H_{⋎}^{\upsilon \mu \omega }\right)}_{2}$ if

$${\left(H_{e}^{\upsilon }\right)}_{2}({£}_{\sigma },\hbar_{\alpha })\leq {\left(H_{e}^{\upsilon }\right)}_{1}({£}_{\sigma },\hbar_{\alpha }),\;\;\;\;\;{\left(H_{e}^{\mu }\right)}_{2}({£}_{\sigma },\hbar_{\alpha })\geq {\left(H_{e}^{\mu }\right)}_{1}({£}_{\sigma },\hbar_{\alpha }),$$

$$\;\;{\left(H_{e}^{\omega }\right)}_{2}({£}_{\sigma },\hbar_{\alpha })\geq {\left(H_{e}^{\omega }\right)}_{1}({£}_{\sigma },\hbar_{\alpha }),\;\forall \;{£}_{\sigma },\hbar_{\alpha }\in \overset{˜}{\left(¥,⋎\right)},e\in ⋎.$$

Suppose $\left(¥,{\left(H_{⋎}^{\upsilon \mu \omega }\right)}_{1}\right)$ and $\left(U,{\left(H_{R}^{\upsilon \mu \omega }\right)}_{2}\right)$ be two *svns-topogenous order spaces*.

Then a map $\psi_{{}_{\varphi }}:\overset{˜}{\left(¥,⋎\right)}\to \overset{˜}{\left(U,R\right)}$ is called *snv-soft topogenous continuous* iff

$${\left(H_{\varphi (e)}^{\upsilon }\right)}_{2}({£}_{\sigma },\hbar_{\alpha })\leq {\left(H_{e}^{\upsilon }\right)}_{1}(\psi_{{}_{\varphi }}^{-1}({£}_{\sigma }),\psi_{{}_{\varphi }}^{-1}(\hbar_{\alpha })),$$

$${\left(H_{\varphi (e)}^{\mu }\right)}_{2}({£}_{\sigma },\hbar_{\alpha })\geq {\left(H_{e}^{\mu }\right)}_{1}(\psi_{{}_{\varphi }}^{-1}({£}_{\sigma }),\psi_{{}_{\varphi }}^{-1}(\hbar_{\alpha })),$$

$${\left(H_{\varphi (e)}^{\omega }\right)}_{2}({£}_{\sigma },\hbar_{\alpha })\geq {\left(H_{e}^{\omega }\right)}_{1}(\psi_{{}_{\varphi }}^{-1}({£}_{\sigma }),\psi_{{}_{\varphi }}^{-1}(\hbar_{\alpha })),$$

for any ${£}_{\sigma },\hbar_{\alpha }\in \overset{˜}{\left(U,R\right)}$, e∈⋎.

Theorem 5 *Let*$\left(¥,H_{⋎}^{\upsilon \mu \omega }\right)$*be* svns-topogenous order space*. Define*

$${\left(H_{st}^{\upsilon }\right)}_{e}({£}_{\sigma },\hbar_{\alpha })=\underset{\left\{{\left({£}_{\sigma }\right)}_{j},{\left(\hbar_{\alpha }\right)}_{j},{\overset{˜}{⋎}}^{\iota_{j}}|j\in J\right\}\in N({£}_{\sigma },\hbar_{\alpha })}{⋁}\left\{\underset{\left({\left({£}_{\sigma }\right)}_{i},{\left(\hbar_{\alpha }\right)}_{i},{\overset{˜}{⋎}}^{\iota_{i}}\right)\in A}{⋀}H_{e}^{\upsilon }({\left({£}_{\sigma }\right)}_{i},{\left(\hbar_{\alpha }\right)}_{i})\right\},$$

$${\left(H_{st}^{\mu }\right)}_{e}({£}_{\sigma },\hbar_{\alpha })=\underset{\left\{{\left({£}_{\sigma }\right)}_{j},{\left(\hbar_{\alpha }\right)}_{j},{\overset{˜}{⋎}}^{\iota_{j}}|j\in J\right\}\in N({£}_{\sigma },\hbar_{\alpha })}{⋀}\left\{\underset{\left({\left({£}_{\sigma }\right)}_{i},{\left(\hbar_{\alpha }\right)}_{i},{\overset{˜}{⋎}}^{\iota_{i}}\right)\in A}{⋁}H_{e}^{\mu }({\left({£}_{\sigma }\right)}_{i},{\left(\hbar_{\alpha }\right)}_{i})\right\},$$

$${\left(H_{st}^{\omega }\right)}_{e}({£}_{\sigma },\hbar_{\alpha })=\underset{\left\{{\left({£}_{\sigma }\right)}_{j},{\left(\hbar_{\alpha }\right)}_{j},{\overset{˜}{⋎}}^{\iota_{j}}|j\in J\right\}\in N({£}_{\sigma },\hbar_{\alpha })}{⋀}\left\{\underset{\left({\left({£}_{\sigma }\right)}_{i},{\left(\hbar_{\alpha }\right)}_{i},{\overset{˜}{⋎}}^{\iota_{i}}\right)\in A}{⋁}H_{e}^{\omega }({\left({£}_{\sigma }\right)}_{i},{\left(\hbar_{\alpha }\right)}_{i})\right\},$$

*where* $A=\left\{{\left({£}_{\sigma }\right)}_{j},{\left(\hbar_{\alpha }\right)}_{j},{\overset{˜}{⋎}}^{\iota_{j}}|j\in J\right\}$ *and* $N({£}_{\sigma },\hbar_{\alpha })=\{\{({\left({£}_{\sigma }\right)}_{j},{\left(\hbar_{\alpha }\right)}_{j},{\overset{˜}{⋎}}^{\iota_{j}})|j\in J,J\;is\;finite)\}|{£}_{\sigma }⊑{⊔}_{j\in J}({\left({£}_{\sigma }\right)}_{j}⊓{\overset{˜}{⋎}}^{\iota_{j}})\;and\;\hbar_{\alpha }⊒{⊔}_{j\in J}({\left(\hbar_{\alpha }\right)}_{j}⊓{\overset{˜}{⋎}}^{\iota_{j}}),\iota \in \varsigma \}$*. Then* ${\left(H_{st}^{\upsilon \mu \omega }\right)}_{⋎}$ *is the coarsest* stratified svns-topogenous order *on* ¥ *which is finer than* $H_{⋎}^{\upsilon \mu \omega }$*.*

Proof ($H_{1}$), ($H_{2}$), ($H_{3}$) straightforward.

($H_{4}$) (i) Assume that there exist, ${\left({£}_{\sigma }\right)}_{1},{\left(\hbar_{\alpha }\right)}_{1},{\left({£}_{\sigma }\right)}_{2},{\left(\hbar_{\alpha }\right)}_{2}\in \overset{˜}{\left(¥,⋎\right)}$ such that

$${\left(H_{st}^{\upsilon }\right)}_{e}({\left({£}_{\sigma }\right)}_{1}⊔{\left({£}_{\sigma }\right)}_{2},{\left(\hbar_{\alpha }\right)}_{1}⊔{\left(\hbar_{\alpha }\right)}_{2})≱{\left(H_{st}^{\upsilon }\right)}_{e}({\left({£}_{\sigma }\right)}_{1},{\left(\hbar_{\alpha }\right)}_{1})\wedge {\left(H_{st}^{\upsilon }\right)}_{e}({\left({£}_{\sigma }\right)}_{2},{\left(\hbar_{\alpha }\right)}_{2}),$$

$${\left(H_{st}^{\mu }\right)}_{e}({\left({£}_{\sigma }\right)}_{1}⊔{\left({£}_{\sigma }\right)}_{2},{\left(\hbar_{\alpha }\right)}_{1}⊔{\left(\hbar_{\alpha }\right)}_{2})≰{\left(H_{st}^{\mu }\right)}_{e}({\left({£}_{\sigma }\right)}_{1},{\left(\hbar_{\alpha }\right)}_{1})\vee {\left(H_{st}^{\mu }\right)}_{e}({\left({£}_{\sigma }\right)}_{2},{\left(\hbar_{\alpha }\right)}_{2}),$$

$${\left(H_{st}^{\omega }\right)}_{e}({\left({£}_{{}_{A}}\right)}_{1}⊔{\left({£}_{\sigma }\right)}_{2},{\left(\hbar_{\alpha }\right)}_{1}⊔{\left(\hbar_{\alpha }\right)}_{2})≰{\left(H_{st}^{\omega }\right)}_{e}({\left({£}_{\sigma }\right)}_{1},{\left(\hbar_{\alpha }\right)}_{1})\vee {\left(H_{st}^{\omega }\right)}_{e}({\left({£}_{\sigma }\right)}_{2},{\left(\hbar_{\alpha }\right)}_{2}),$$

therefore, there exists $r\in \varsigma_{0}$ such that

$${\left(H_{st}^{\upsilon }\right)}_{e}({\left({£}_{\sigma }\right)}_{1}⊔{\left({£}_{\sigma }\right)}_{2},{\left(\hbar_{\alpha }\right)}_{1}⊔{\left(\hbar_{\alpha }\right)}_{2})<r\leq {\left(H_{st}^{\upsilon }\right)}_{e}({\left({£}_{\sigma }\right)}_{1},{\left(\hbar_{\alpha }\right)}_{1})\wedge {\left(H_{st}^{\upsilon }\right)}_{e}({\left({£}_{\sigma }\right)}_{2},{\left(\hbar_{\alpha }\right)}_{2}),$$

(6) $${\left(H_{st}^{\mu }\right)}_{e}({\left({£}_{\sigma }\right)}_{1}⊔{\left({£}_{\sigma }\right)}_{2},{\left(\hbar_{\alpha }\right)}_{1}⊔{\left(\hbar_{\alpha }\right)}_{2})\geq 1-r>{\left(H_{st}^{\mu }\right)}_{e}({\left({£}_{\sigma }\right)}_{1},{\left(\hbar_{\alpha }\right)}_{1})\vee {\left(H_{st}^{\mu }\right)}_{e}({\left({£}_{\sigma }\right)}_{2},{\left(\hbar_{\alpha }\right)}_{2}),$$

$${\left(H_{st}^{\omega }\right)}_{e}({\left({£}_{\sigma }\right)}_{1}⊔{\left({£}_{\sigma }\right)}_{2},{\left(\hbar_{\alpha }\right)}_{1}⊔{\left(\hbar_{\alpha }\right)}_{2})\geq 1-r>{\left(H_{st}^{\omega }\right)}_{e}({\left({£}_{\sigma }\right)}_{1},{\left(\hbar_{\alpha }\right)}_{1})\vee {\left(H_{st}^{\omega }\right)}_{e}({\left({£}_{\sigma }\right)}_{2},{\left(\hbar_{\alpha }\right)}_{2}).$$

From the concept of ${\left(H_{st}^{\upsilon \mu \omega }\right)}_{⋎}$, there are ∀,i∈Jandk∈Γ, $\left\{({\left({£}_{\sigma }\right)}_{i},{\left(\hbar_{\alpha }\right)}_{i},{\overset{˜}{⋎}}^{\iota_{i}})\}\in N({\left({£}_{\sigma }\right)}_{1},{\left(\hbar_{\alpha }\right)}_{1}\right)$ and $\left\{({\left({£}_{\sigma }\right)}_{k},{\left(\hbar_{\alpha }\right)}_{k},{\overset{˜}{⋎}}^{\iota_{{}_{k}}})\}\in N({\left({£}_{\sigma }\right)}_{2},{\left(\hbar_{\alpha }\right)}_{2}\right)$, such that

$${\left(H_{st}^{\upsilon }\right)}_{e}({\left({£}_{\sigma }\right)}_{1}⊔{\left({£}_{\sigma }\right)}_{2},{\left(\hbar_{\alpha }\right)}_{1}⊔{\left(\hbar_{\alpha }\right)}_{2})\geq \underset{i,k}{⋀}H_{e}^{\upsilon }({\left({£}_{\sigma }\right)}_{i}⊔{\left({£}_{\sigma }\right)}_{k},{\left(\hbar_{\alpha }\right)}_{i}⊔{\left(\hbar_{\alpha }\right)}_{k})\geq \underset{i,k}{⋀}H_{e}^{\upsilon }({\left({£}_{\sigma }\right)}_{i},{\left(\hbar_{\alpha }\right)}_{i})\wedge H_{e}^{\upsilon }({\left({£}_{\sigma }\right)}_{k},{\left(\hbar_{\alpha }\right)}_{k})\geq r,$$

$${\left(H_{st}^{\mu }\right)}_{e}({\left({£}_{\sigma }\right)}_{1}⊔{\left({£}_{\sigma }\right)}_{2},{\left(\hbar_{\alpha }\right)}_{1}⊔{\left(\hbar_{\alpha }\right)}_{2})\leq \underset{i,k}{⋁}H_{e}^{\upsilon }({\left({£}_{\sigma }\right)}_{i}⊔{\left({£}_{\sigma }\right)}_{k},{\left(\hbar_{\alpha }\right)}_{i}⊔{\left(\hbar_{\alpha }\right)}_{k})\leq \underset{i,k}{⋁}H_{e}^{\mu }({\left({£}_{\sigma }\right)}_{i},{\left(\hbar_{\alpha }\right)}_{i})\vee H_{e}^{\mu }({\left({£}_{\sigma }\right)}_{k},{\left(\hbar_{\alpha }\right)}_{k})<1-r,$$

$${\left(H_{st}^{\omega }\right)}_{e}({\left({£}_{\sigma }\right)}_{1}⊔{\left({£}_{\sigma }\right)}_{2},{\left(\hbar_{\alpha }\right)}_{1}⊔{\left(\hbar_{\alpha }\right)}_{2})\leq \underset{i,k}{⋁}H_{e}^{\upsilon }({\left({£}_{\sigma }\right)}_{i}⊔{\left({£}_{\sigma }\right)}_{k},{\left(\hbar_{\alpha }\right)}_{i}⊔{\left(\hbar_{\alpha }\right)}_{k})\leq \underset{i,k}{⋁}H_{e}^{\omega }({\left({£}_{\sigma }\right)}_{i},{\left(\hbar_{\alpha }\right)}_{i})\vee H_{e}^{\omega }({\left({£}_{\sigma }\right)}_{k},{\left(\hbar_{\alpha }\right)}_{k})<1-r.$$

In this case, it is a contradiction with the hypothesis, as is clear from Equation No. (6). Hence, $H_{4}(i)$ holds.

(ii) In the same way as used to prove (i).

($H_{S}$) Since ${\overset{˜}{⋎}}^{\iota }={\overset{˜}{⋎}}^{\iota }⊓\overset{˜}{⋎}$,

$${\left(H_{st}^{\upsilon }\right)}_{e}({\overset{˜}{⋎}}^{\iota },{\overset{˜}{⋎}}^{\iota })\geq H_{e}^{\upsilon }(\overset{˜}{⋎},\overset{˜}{⋎})=1,\;\;\;\;{\left(H_{st}^{\mu }\right)}_{e}({\overset{˜}{⋎}}^{\iota },{\overset{˜}{⋎}}^{\iota })\leq H_{e}^{\mu }(\overset{˜}{⋎},\overset{˜}{⋎})=0,$$

$${\left(H_{st}^{\omega }\right)}_{e}({\overset{˜}{⋎}}^{\iota },{\overset{˜}{⋎}}^{\iota })\leq H_{e}^{\omega }(\overset{˜}{⋎},\overset{˜}{⋎})=0$$

Hence, ${\left(H_{st}^{\upsilon }\right)}_{e}({\overset{˜}{⋎}}^{\iota },{\overset{˜}{⋎}}^{\iota })=1$, ${\left(H_{st}^{\mu }\right)}_{e}({\overset{˜}{⋎}}^{\iota },{\overset{˜}{⋎}}^{\iota })=0$ and ${\left(H_{st}^{\omega }\right)}_{e}({\overset{˜}{⋎}}^{\iota },{\overset{˜}{⋎}}^{\iota })=0$, ∀ ι∈ς.

On another side, since ${£}_{\sigma }⊑{£}_{\sigma }⊓\overset{˜}{⋎}$ and $\hbar_{\alpha }⊒\hbar_{\alpha }⊓\overset{˜}{⋎}$ we obtain

$${\left(H_{st}^{\upsilon }\right)}_{e}({£}_{\sigma },\hbar_{\alpha })\geq H_{e}^{\upsilon }({£}_{\sigma },\hbar_{\alpha }),\;\;\;\;\;\;{\left(H_{st}^{\mu }\right)}_{e}({£}_{\sigma },\hbar_{\alpha })\leq H_{e}^{\mu }({£}_{\sigma },\hbar_{\alpha }),$$

$${\left(H_{st}^{\omega }\right)}_{e}({£}_{\sigma },\hbar_{\alpha })\leq H_{e}^{\omega }({£}_{\sigma },\hbar_{\alpha }),\;\forall \;{£}_{\sigma },\hbar_{\alpha }\in \overset{˜}{\left(¥,⋎\right)}.$$

Hence, ${\left(H_{st}^{\upsilon \mu \omega }\right)}_{⋎}$ is the *stratified svns-topogenous order* on ⋎ which is finer than $H_{⋎}^{\upsilon \mu \omega }$.

Finally, suppose that ${\left(H_{⋎}^{\upsilon \mu \omega }\right)}^{⋆}$ be *stratified svns-topogenous* and finer than $H_{⋎}^{\upsilon \mu \omega }$ then, $\;\forall \;{£}_{\sigma },\hbar_{\alpha }\in \overset{˜}{\left(¥,⋎\right)},\iota \in \varsigma ,e\in ⋎$.

$${\left(H_{e}^{\upsilon }\right)}^{⋆}({£}_{\sigma },\hbar_{\alpha })\geq H_{e}^{\upsilon }({£}_{\sigma },\hbar_{\alpha }),\;\;\;\;\;\;{\left(H_{e}^{\mu }\right)}^{⋆}({£}_{\sigma },\hbar_{\alpha })\leq H_{e}^{\mu }({£}_{\sigma },\hbar_{\alpha }),$$

$${\left(H_{e}^{\omega }\right)}^{⋆}({£}_{\sigma },\hbar_{\alpha })\leq H_{e}^{\omega }({£}_{\sigma },\hbar_{\alpha }).$$

Now we will prove that

$${\left(H_{e}^{\upsilon }\right)}^{⋆}({£}_{\sigma },\hbar_{\alpha })\geq {\left(H_{st}^{\upsilon }\right)}_{e}({£}_{\sigma },\hbar_{\alpha }),\;\;\;\;\;\;{\left(H_{e}^{\mu }\right)}^{⋆}({£}_{\sigma },\hbar_{\alpha })\leq {\left(H_{st}^{\mu }\right)}_{e}({£}_{\sigma },\hbar_{\alpha }),$$

$${\left(H_{e}^{\omega }\right)}^{⋆}({£}_{\sigma },\hbar_{\alpha })\leq {\left(H_{st}^{\omega }\right)}_{e}({£}_{\sigma },\hbar_{\alpha }).$$

Suppose there exist ${£}_{\sigma },\hbar_{\alpha }\in \overset{˜}{(¥,⋎}),\;e\in ⋎$ such that

$${\left(H_{e}^{\upsilon }\right)}^{⋆}({£}_{\sigma },\hbar_{\alpha })≱{\left(H_{st}^{\upsilon }\right)}_{e}({£}_{\sigma },\hbar_{\alpha }),\;\;\;\;\;\;{\left(H_{e}^{\mu }\right)}^{⋆}({£}_{\sigma },\hbar_{\alpha })≰{\left(H_{st}^{\mu }\right)}_{e}({£}_{\sigma },\hbar_{\alpha }),$$

$${\left(H_{e}^{\omega }\right)}^{⋆}({£}_{\sigma },\hbar_{\alpha })≰{\left(H_{st}^{\omega }\right)}_{e}({£}_{\sigma },\hbar_{\alpha }),$$

then there is r∈ς such that

$${\left(H_{e}^{\upsilon }\right)}^{⋆}({£}_{\sigma },\hbar_{\alpha })<r\leq {\left(H_{st}^{\upsilon }\right)}_{e}({£}_{\sigma },\hbar_{\alpha }),$$

(7) $${\left(H_{e}^{\mu }\right)}^{⋆}({£}_{\sigma },\hbar_{\alpha })\geq 1-r>{\left(H_{st}^{\mu }\right)}_{e}({£}_{\sigma },\hbar_{\alpha }),$$

$${\left(H_{e}^{\omega }\right)}^{⋆}({£}_{\sigma },\hbar_{\alpha })\geq 1-r>{\left(H_{st}^{\omega }\right)}_{e}({£}_{\sigma },\hbar_{\alpha }).$$

From the concept of ${\left(H_{st}^{\upsilon \mu \omega }\right)}_{⋎}$, there exists $A=\{({\left({£}_{\sigma }\right)}_{j},{\left(\hbar_{\alpha }\right)}_{j},{\overset{˜}{⋎}}^{\iota_{j}})\}\in N({£}_{\sigma },\hbar_{\alpha })$, for every j∈J such that

$${\left(H_{e}^{\upsilon }\right)}^{⋆}({£}_{\sigma },\hbar_{\alpha })\geq {\left(H_{e}^{\upsilon }\right)}^{⋆}\left({⊔}_{j\in J}[{\left({£}_{\sigma }\right)}_{j}⊓{\overset{˜}{⋎}}^{\iota_{j}}],\;{⊔}_{j\in J}[{\left(\hbar_{\alpha }\right)}_{j}⊓{\overset{˜}{⋎}}^{\iota_{j}}]\right)\geq \underset{j\in J}{⋀}{\left(H_{e}^{\upsilon }\right)}^{⋆}\left({\left({£}_{\sigma }\right)}_{j}⊓{\overset{˜}{⋎}}^{\iota_{j}},\;{\left(\hbar_{\alpha }\right)}_{j}⊓{\overset{˜}{⋎}}^{\iota_{j}}\right)\geq \underset{j\in J}{⋀}\left[{\left(H_{e}^{\upsilon }\right)}^{⋆}({\left({£}_{\sigma }\right)}_{j},{\left(\hbar_{\alpha }\right)}_{j})\wedge {\left(H_{e}^{\upsilon }\right)}^{⋆}({\overset{˜}{⋎}}^{\iota_{j}},{\overset{˜}{⋎}}^{\iota_{j}})\right]\geq \underset{\left({\left({£}_{\sigma }\right)}_{i},{\left(\hbar_{\alpha }\right)}_{i},{\overset{˜}{⋎}}^{\iota_{i}}\right)\in A}{⋀}H_{e}^{\upsilon }({\left({£}_{\sigma }\right)}_{i},{\left(\hbar_{\alpha }\right)}_{i})\geq r,$$

$${\left(H_{e}^{\mu }\right)}^{⋆}({£}_{\sigma },\hbar_{\alpha })\leq {\left(H_{e}^{\mu }\right)}^{⋆}\left({⊔}_{j\in J}[{\left({£}_{\sigma }\right)}_{j}⊓{\overset{˜}{⋎}}^{\iota_{j}}],\;{⊔}_{j\in J}[{\left(\hbar_{\alpha }\right)}_{j}⊓{\overset{˜}{⋎}}^{\iota_{j}}]\right)\leq \underset{j\in J}{⋁}{\left(H_{e}^{\mu }\right)}^{⋆}({\left({£}_{\sigma }\right)}_{j}⊓{\overset{˜}{⋎}}^{\iota_{j}},\;{\left(\hbar_{\alpha }\right)}_{j}⊓{\overset{˜}{⋎}}^{\iota_{j}})\leq \underset{j\in J}{⋁}\left[{\left(H_{e}^{\mu }\right)}^{⋆}({\left({£}_{\sigma }\right)}_{j},{\left(\hbar_{\alpha }\right)}_{j})\vee {\left(H_{e}^{\mu }\right)}^{⋆}({\overset{˜}{⋎}}^{\iota_{j}},{\overset{˜}{⋎}}^{\iota_{j}})\right]\leq \underset{\left({\left({£}_{\sigma }\right)}_{i},{\left(\hbar_{v}\right)}_{i},{\overset{˜}{⋎}}^{\iota_{i}}\right)\in A}{⋁}H_{e}^{\mu }({\left({£}_{\sigma }\right)}_{i},{\left(\hbar_{\alpha }\right)}_{i})\leq 1-r,$$

$${\left(H_{e}^{\omega }\right)}^{⋆}({£}_{\sigma },\hbar_{\alpha })\leq {\left(H_{e}^{\omega }\right)}^{⋆}\left({⊔}_{j\in J}[{\left({£}_{\sigma }\right)}_{j}⊓{\overset{˜}{⋎}}^{\iota_{j}}],\;{⊔}_{j\in J}[{\left(\hbar_{\alpha }\right)}_{j}⊓{\overset{˜}{⋎}}^{\iota_{j}}]\right)\leq \underset{j\in J}{⋁}{\left(H_{e}^{\omega }\right)}^{⋆}({\left({£}_{\sigma }\right)}_{j}⊓{\overset{˜}{⋎}}^{\iota_{j}},\;{\left(\hbar_{\alpha }\right)}_{j}⊓{\overset{˜}{⋎}}^{\iota_{j}})\leq \underset{j\in J}{⋁}\left[{\left(H_{e}^{\omega }\right)}^{⋆}({\left({£}_{\sigma }\right)}_{j},{\left(\hbar_{\alpha }\right)}_{j})\vee {\left(H_{e}^{\omega }\right)}^{⋆}({\overset{˜}{⋎}}^{\iota_{j}},{\overset{˜}{⋎}}^{\iota_{j}})\right]\leq \underset{\left({\left({£}_{\sigma }\right)}_{i},{\left(\hbar_{\alpha }\right)}_{i},{\overset{˜}{⋎}}^{\iota_{i}}\right)\in A}{⋁}H_{e}^{\omega }({\left({£}_{\sigma }\right)}_{i},{\left(\hbar_{\alpha }\right)}_{i})\leq 1-r.$$

In this instance, the hypothesis is contradicted as is clear from Equations (7). Therefore, the coarsest *stratified svns- topogenous order* on ⋎ is ${\left(H_{st}^{\upsilon \mu \omega }\right)}_{⋎}$ which is finer than $H_{⋎}^{\upsilon \mu \omega }$. □

Theorem 6 *Let* $\left(¥,H_{⋎}^{\upsilon \mu \omega }\right)$ *and* $\left(U,{\left(H_{R}^{\upsilon \mu \omega }\right)}^{{}^{⋆}}\right)$ *be two svns-topogenous order spaces, if* $\psi_{{}_{\varphi }}:(⋎,H_{⋎}^{\upsilon \mu \omega })\to (U,{\left(H_{R}^{\upsilon \mu \omega }\right)}^{{}^{⋆}})$ *be a svns-topogenous continuous, then* $\psi_{{}_{\varphi }}:(¥,{\left(H_{⋎}^{\upsilon \mu \omega }\right)}_{st})\to (U,{\left(H_{R}^{\upsilon \mu \omega }\right)}_{\text{st}}^{{}^{⋆}})$ *is a svns-topogenous continuous.*

Proof Assume that ${£}_{\sigma },\hbar_{\alpha }\in \overset{˜}{\left(U,R\right)}$ with ${⊔}_{j\in J}({\left({£}_{\sigma }\right)}_{j}⊓{\overset{˜}{R}}^{\iota_{j}})⊒{£}_{\sigma }$ and ${⊔}_{j\in J}({\left(\hbar_{\alpha }\right)}_{j}⊓{\overset{˜}{R}}^{\iota_{j}})⊑\hbar_{\alpha }$. Then ${⊔}_{j\in J}\psi_{{}_{\varphi }}^{-1}{\left({£}_{\sigma }\right)}_{j}⊓{\overset{˜}{⋎}}^{\iota_{j}}⊒\psi_{{}_{\varphi }}^{-1}({£}_{\sigma })$ and ${⊔}_{j\in J}\psi_{{}_{\varphi }}^{-1}{\left(\hbar_{\alpha }\right)}_{j})⊓{\overset{˜}{⋎}}^{\iota_{j}}⊑\psi_{{}_{\varphi }}^{-1}(\hbar_{\alpha })$. For all collections $N({£}_{\sigma },\hbar_{\alpha })=\{\{B=\{({\left({£}_{\sigma }\right)}_{j},{\left(\hbar_{\alpha }\right)}_{j},{\overset{˜}{R}}^{\iota_{j}})\}|{£}_{\sigma }⊑{⊔}_{j\in J}({\left({£}_{\sigma }\right)}_{j}⊓{\overset{˜}{R}}^{\iota_{j}})\;and\;\hbar_{\alpha }⊒{⊔}_{j\in J}({\left(\hbar_{\alpha }\right)}_{j}⊓{\overset{˜}{R}}^{\iota_{j}})\}$ ∀, |j∈J and ι∈ς, we have

$${\left(H_{e}^{\upsilon }\right)}_{st}(\psi_{{}_{\varphi }}^{-1}({£}_{\sigma }),\psi_{{}_{\varphi }}^{-1}(\hbar_{\alpha }))\geq \underset{\left(\psi_{{}_{\varphi }}^{-1}({\left({£}_{\sigma }\right)}_{j_{{}_{l}}}),\psi_{{}_{\varphi }}^{-1}({\left(\hbar_{\alpha }\right)}_{j_{{}_{l}}}),{\overset{˜}{⋎}}^{\iota_{j_{{}_{l}}}}\right)\in \left\{\psi_{{}_{\varphi }}^{-1}({\left({£}_{\sigma }\right)}_{j}),\psi_{{}_{\varphi }}^{-1}({\left(\hbar_{\alpha }\right)}_{j}),{\overset{˜}{⋎}}^{\iota_{j}}|j\in J\right\}}{⋁}\;H_{e}^{\upsilon }\left(\psi_{{}_{\varphi }}^{-1}({\left({£}_{\sigma }\right)}_{j_{{}_{l}}}),\psi_{{}_{\varphi }}^{-1}({\left(\hbar_{\alpha }\right)}_{j_{{}_{l}}})\right)\geq \underset{\left({\left({£}_{\sigma }\right)}_{j_{{}_{l}}},{\left(\hbar_{\alpha }\right)}_{j_{{}_{l}}}),{\overset{˜}{R}}^{\iota_{j_{{}_{l}}}}\right)\in B}{⋁}{\left(H_{\varphi (e)}^{\upsilon }\right)}^{⋆}({\left({£}_{\sigma }\right)}_{j_{{}_{l}}},{\left(\hbar_{\alpha }\right)}_{j_{{}_{l}}})\geq \underset{N({£}_{\sigma },\hbar_{\alpha })}{⋀}\left(\underset{\left({\left({£}_{\sigma }\right)}_{j_{{}_{l}}},{\left(\hbar_{\alpha }\right)}_{j_{{}_{l}}}),{\overset{˜}{R}}^{\iota_{j_{{}_{l}}}}\right)\in B}{⋁}{\left(H_{\varphi (e)}^{\upsilon }\right)}^{⋆}({\left({£}_{\sigma }\right)}_{j_{{}_{l}}},{\left(\hbar_{\alpha }\right)}_{j_{{}_{l}}})\right)={\left(H_{\varphi (e)}^{\upsilon }\right)}_{st}^{{}^{⋆}}({£}_{\sigma },\hbar_{\alpha })$$

$${\left(H_{e}^{\mu }\right)}_{st}\left(\psi_{{}_{\varphi }}^{-1}({£}_{\sigma }),\psi_{{}_{\varphi }}^{-1}(\hbar_{\alpha })\right)\leq \underset{\left(\psi_{{}_{\varphi }}^{-1}({\left({£}_{\sigma }\right)}_{j_{{}_{l}}}),\psi_{{}_{\varphi }}^{-1}({\left(\hbar_{\alpha }\right)}_{j_{{}_{l}}}),{\overset{˜}{⋎}}^{\iota_{j_{{}_{l}}}}\right)\in \left\{\psi_{{}_{\varphi }}^{-1}({\left({£}_{\sigma }\right)}_{j}),\psi_{{}_{\varphi }}^{-1}({\left(\hbar_{\alpha }\right)}_{j}),{\overset{˜}{⋎}}^{\iota_{j}}|j\in J\right\}}{⋀}\;H_{e}^{\mu }\left(\psi_{{}_{\varphi }}^{-1}({\left({£}_{\sigma }\right)}_{j_{{}_{l}}}),\psi_{{}_{\varphi }}^{-1}({\left(\hbar_{\alpha }\right)}_{j_{{}_{l}}})\right)\leq \underset{\left({\left({£}_{\sigma }\right)}_{j_{{}_{l}}},{\left(\hbar_{\alpha }\right)}_{j_{{}_{l}}}),{\overset{˜}{R}}^{\iota_{j_{{}_{l}}}}\right)\in B}{⋀}{\left(H_{\varphi (e)}^{\mu }\right)}^{⋆}({\left({£}_{\sigma }\right)}_{j_{{}_{l}}},{\left(\hbar_{\alpha }\right)}_{j_{{}_{l}}})\leq \underset{N({£}_{\sigma \},\hbar_{\alpha }})}{⋁}\left(\underset{\left({\left({£}_{\sigma }\right)}_{j_{{}_{l}}},{\left(\hbar_{\alpha }\right)}_{j_{{}_{l}}},{\overset{˜}{R}}^{\iota_{j_{{}_{l}}}}\right)\in B}{⋀}{\left(H_{\varphi (e)}^{\mu }\right)}^{⋆}({\left({£}_{\sigma }\right)}_{j_{{}_{l}}},{\left(\hbar_{\alpha }\right)}_{j_{{}_{l}}})\right)={\left(H_{\varphi (e)}^{\mu }\right)}_{st}^{{}^{⋆}}({£}_{\sigma },\hbar_{\alpha })$$

$${\left(H_{e}^{\omega }\right)}_{st}\left(\psi_{{}_{\varphi }}^{-1}({£}_{\sigma }),\psi_{{}_{\varphi }}^{-1}(\hbar_{\alpha })\right)\leq \underset{\left(\psi_{{}_{\varphi }}^{-1}({\left({£}_{\sigma }\right)}_{j_{{}_{l}}}),\psi_{{}_{\varphi }}^{-1}({\left(\hbar_{\alpha }\right)}_{j_{{}_{l}}}),{\overset{˜}{\text{E}}}^{\iota_{j_{{}_{l}}}}\right)\in \left\{\psi_{{}_{\varphi }}^{-1}({\left({£}_{\sigma }\right)}_{j}),\psi_{{}_{\varphi }}^{-1}({\left(\hbar_{\alpha }\right)}_{j}),{\overset{˜}{⋎}}^{\iota_{j}}|j\in J\right\}}{⋀}\;H_{e}^{\omega }\left(\psi_{{}_{\varphi }}^{-1}({\left({£}_{\sigma }\right)}_{j_{{}_{l}}}),\psi_{{}_{\varphi }}^{-1}({\left(\hbar_{\alpha }\right)}_{j_{{}_{l}}})\right)\leq \underset{\left({\left({£}_{\sigma }\right)}_{j_{{}_{l}}},{\left(\hbar_{\alpha }\right)}_{j_{{}_{l}}}),{\overset{˜}{R}}^{\iota_{j_{{}_{l}}}}\right)\in B}{⋀}{\left(H_{\varphi (e)}^{\omega }\right)}^{⋆}({\left({£}_{\sigma }\right)}_{j_{{}_{l}}},{\left(\hbar_{\alpha }\right)}_{j_{{}_{l}}})\leq \underset{N({£}_{\sigma },\hbar_{\alpha })}{⋁}\left(\underset{\left({\left({£}_{\sigma }\right)}_{j_{{}_{l}}},{\left(\hbar_{\alpha }\right)}_{j_{{}_{l}}},{\overset{˜}{R}}^{\iota_{j_{{}_{l}}}}\right)\in B}{⋀}{\left(H_{\varphi (e)}^{\omega }\right)}^{⋆}({\left({£}_{\sigma }\right)}_{j_{{}_{l}}},{\left(\hbar_{\alpha }\right)}_{j_{{}_{l}}})\right)={\left(H_{\varphi (e)}^{\omega }\right)}_{st}^{{}^{⋆}}({£}_{\sigma },\hbar_{\alpha })$$

 □

Theorem 7 *(1) If* $H_{⋎}^{\upsilon \mu \omega }$ *is symmetrical svns-topogenous order, then* ${\left(H_{st}^{\upsilon \mu \omega }\right)}_{⋎}$ *is also symmetrical svns-topogenous order.*

*(2) If* $H_{⋎}^{\upsilon \mu \omega }$ *is perfect svns-topogenous order, then*

*(i)* ${\left(T_{H}^{\upsilon }\right)}_{e}({£}_{\sigma })=H_{e}^{\upsilon }({£}_{\sigma },{£}_{\sigma })$ *,* ${\left(T_{H}^{\mu }\right)}_{e}({£}_{\sigma })=H_{e}^{\mu }({£}_{\sigma },{£}_{\sigma })$ *and* ${\left(T_{H}^{\omega }\right)}_{e}({£}_{\sigma })=$

$H_{e}^{\omega }({£}_{\sigma },{£}_{\sigma })$ *is svns-topology related by* $H_{⋎}^{\upsilon \mu \omega }$

*(ii)* ${\left(T_{H_{st}}^{\upsilon \mu \omega }\right)}_{⋎}={\left[{\left(T_{H}^{\upsilon \mu \omega }\right)}_{st}\right]}_{{}_{⋎}}$ *.*

*(3) If* $T_{⋎}^{\upsilon \mu \omega }$ *is a svnst, then*

*(i)* ${\left(H_{{}_{T}}^{\upsilon }\right)}_{e}({£}_{\sigma },\hbar_{\alpha })=⋁\{T_{e}^{\upsilon }(g_{{}_{C}})|{£}_{\sigma }⊑g_{{}_{C}}⊑\hbar_{\alpha }\}$ *,* ${\left(H_{{}_{T}}^{\mu }\right)}_{e}({£}_{\sigma },\hbar_{\alpha })=⋀\{T_{e}^{\mu }(g_{{}_{C}})|$

${£}_{\sigma }⊑g_{{}_{C}}⊑\hbar_{\alpha }\}$ *and* ${\left(H_{{}_{T}}^{\omega }\right)}_{e}({£}_{\sigma },\hbar_{\alpha })=⋀\{T_{e}^{\omega }(g_{{}_{C}})|{£}_{\sigma }⊑g_{{}_{C}}⊑\hbar_{\alpha }\}$ *is perfect svns-topogenous order related by* $T_{⋎}^{\upsilon \mu \omega }$ *.*

*(ii)* ${\left(H_{{}_{T_{st}}}^{\upsilon \mu \omega }\right)}_{⋎}={\left[{\left(H_{T}^{\upsilon \mu \omega }\right)}_{{}_{st}}\right]}_{⋎}$ *is a perfect svns-topogenous order.*

Proof (1) We will prove that for each ${£}_{\sigma },\hbar_{\alpha }\in \overset{˜}{\left(¥,⋎\right)}$, then

$${\left(H_{st}^{\upsilon }\right)}_{e}({£}_{\sigma },\hbar_{\alpha })={\left(H_{st}^{\upsilon }\right)}_{e}(\hbar_{\alpha }^{c},{£}_{\sigma }^{c}),\;\;\;\;{\left(H_{st}^{\mu }\right)}_{e}({£}_{\sigma },\hbar_{\alpha })={\left(H_{st}^{\mu }\right)}_{e}(\hbar_{\alpha }^{c},{£}_{\sigma }^{c}),$$

$${\left(H_{st}^{\omega }\right)}_{e}({£}_{\sigma },\hbar_{\alpha })={\left(H_{st}^{\omega }\right)}_{e}(\hbar_{\alpha }^{c},{£}_{\sigma }^{c}).$$

Suppose that

$${\left(H_{st}^{\upsilon }\right)}_{e}({£}_{\sigma },\hbar_{\alpha })≰{\left(H_{st}^{\upsilon }\right)}_{e}(\hbar_{\alpha }^{c},{£}_{\sigma }^{c}),\;\;\;\;{\left(H_{st}^{\mu }\right)}_{e}({£}_{\sigma },\hbar_{\alpha })≱{\left(H_{st}^{\mu }\right)}_{e}(\hbar_{\alpha }^{c},{£}_{\sigma }^{c})$$

$${\left(H_{st}^{\omega }\right)}_{e}({£}_{\sigma },\hbar_{\alpha })≱{\left(H_{st}^{\omega }\right)}_{e}(\hbar_{\alpha }^{c},{£}_{\sigma }^{c}).$$

From the concept of ${\left(H_{st}^{\upsilon \mu \omega }\right)}_{⋎}$, there are $A=\left\{{\left({£}_{\sigma }\right)}_{{}_{j}},{\left(\hbar_{\alpha }\right)}_{j},{\overset{˜}{⋎}}^{\iota_{j}}|j\in J\right\}\in N({£}_{\sigma },\hbar_{\alpha })$ such that

$${\left(H_{st}^{\upsilon }\right)}_{e}(\hbar_{\alpha }^{c},{£}_{\sigma }^{c})\leq r\leq \underset{\left({\left({£}_{\sigma }\right)}_{i},{\left(\hbar_{\alpha }\right)}_{i},{\overset{˜}{⋎}}^{\iota_{i}}\right)\in A}{⋀}H_{e}^{\upsilon }({\left({£}_{\sigma }\right)}_{i},{\left(\hbar_{\alpha }\right)}_{i})$$

(8) $${\left(H_{st}^{\mu }\right)}_{e}(\hbar_{\alpha }^{c},{£}_{\sigma }^{c})\geq 1-r\geq \underset{\left({\left({£}_{\sigma }\right)}_{i},{\left(\hbar_{\alpha }\right)}_{i},{\overset{˜}{⋎}}^{\iota_{i}}\right)\in A}{⋁}H_{e}^{\mu }({\left({£}_{\sigma }\right)}_{i},{\left(\hbar_{\alpha }\right)}_{i})$$

$${\left(H_{st}^{\omega }\right)}_{e}(\hbar_{\alpha }^{c},{£}_{\sigma }^{c})\geq 1-r\geq \underset{\left({\left({£}_{\sigma }\right)}_{i},{\left(\hbar_{\alpha }\right)}_{i},{\overset{˜}{⋎}}^{\iota_{i}}\right)\in A}{⋁}H_{e}^{\omega }({\left({£}_{\sigma }\right)}_{i},{\left(\hbar_{\alpha }\right)}_{i}).$$

On another side,

$${\left(H_{st}^{\upsilon }\right)}_{e}(\hbar_{\alpha }^{c},{£}_{\sigma }^{c})\geq {\left(H_{st}^{\upsilon }\right)}_{e}\left[{⊓}_{j\in J}\left({\left(\hbar_{\alpha }^{c}\right)}_{j}⊔{\overset{˜}{⋎}}^{1-\iota_{{}_{j}}}\right),\;{⊓}_{j\in J}\left({\left({£}_{\sigma }^{c}\right)}_{j}⊔{\overset{˜}{⋎}}^{1-\iota_{{}_{j}}}\right)\right]\geq \underset{\left({\left({£}_{\sigma }\right)}_{i},{\left(\hbar_{\alpha }\right)}_{i},{\overset{˜}{⋎}}^{\iota_{i}}\right)\in A}{⋀}{\left(H_{st}^{\upsilon }\right)}_{e}\left[{\left(\hbar_{\alpha }^{c}\right)}_{i}⊔{\overset{˜}{⋎}}^{1-\iota_{{}_{i}}},{\left({£}_{\sigma }^{c}\right)}_{i}⊔{\overset{˜}{⋎}}^{1-\iota_{{}_{i}}}\right]\geq \underset{\left({\left({£}_{\sigma }\right)}_{i},{\left(\hbar_{\alpha }\right)}_{i},{\overset{˜}{⋎}}^{\iota_{i}}\right)\in A}{⋀}{\left(H_{st}^{\upsilon }\right)}_{e}\left({\left(\hbar_{\alpha }^{c}\right)}_{i},{\left({£}_{\sigma }^{c}\right)}_{i}\right)\wedge {\left(H_{st}^{\upsilon }\right)}_{e}\left({\overset{˜}{⋎}}^{1-\iota_{{}_{i}}},{\overset{˜}{⋎}}^{1-\iota_{{}_{i}}}\right)=\underset{\left({\left({£}_{\sigma }\right)}_{i},{\left(\hbar_{\alpha }\right)}_{i},{\overset{˜}{⋎}}^{\iota_{i}}\right)\in A}{⋀}{\left(H_{st}^{\upsilon }\right)}_{e}\left({\left(\hbar_{\alpha }^{c}\right)}_{i},{\left({£}_{\sigma }^{c}\right)}_{i}\right)\geq \underset{\left({\left({£}_{\alpha }\right)}_{i},{\left(\hbar_{\alpha }\right)}_{i},{\overset{˜}{⋎}}^{\iota_{i}}\right)\in A}{⋀}H_{e}^{\upsilon }\left({\left(\hbar_{\alpha }^{c}\right)}_{i},{\left({£}_{\sigma }^{c}\right)}_{i}\right)=\underset{\left({\left({£}_{\sigma }\right)}_{i},{\left(\hbar_{\alpha }\right)}_{i},{\overset{˜}{⋎}}^{\iota_{i}}\right)\in A}{⋀}H_{e}^{\upsilon }{\left({\left({£}_{\sigma }\right)}_{i},\hbar_{\alpha }\right)}_{i})\geq r.$$

Similarly, by using an analogous line of reasoning, we can show that ${\left(H_{st}^{\mu }\right)}_{e}(\hbar_{\alpha }^{c},{£}_{\sigma }^{c})\leq 1-r$ and ${\left(H_{st}^{\omega }\right)}_{e}(\hbar_{\alpha }^{c},{£}_{\sigma }^{c})\leq r-1$ are incompatible with equations (8). Hence,

$${\left(H_{st}^{\upsilon }\right)}_{e}({£}_{\sigma },\hbar_{\alpha })\leq {\left(H_{st}^{\upsilon }\right)}_{e}(\hbar_{\alpha }^{c},{£}_{\sigma }^{c}),\;\;\;\;{\left(H_{st}^{\mu }\right)}_{e}({£}_{\sigma },\hbar_{\alpha })\geq {\left(H_{st}^{\mu }\right)}_{e}(\hbar_{\alpha }^{c},{£}_{\sigma }^{c})$$

(9) $${\left(H_{st}^{\omega }\right)}_{e}({£}_{\sigma },\hbar_{{}_{B}})\geq {\left(H_{st}^{\omega }\right)}_{e}(\hbar_{\alpha }^{c},{£}_{\sigma }^{c}).$$

Similarly, we can establish through a parallel argument that

$${\left(H_{st}^{\upsilon }\right)}_{e}({£}_{\sigma },\hbar_{\alpha })\geq {\left(H_{st}^{\upsilon }\right)}_{e}(\hbar_{\alpha }^{c},{£}_{\sigma }^{c}),\;\;\;\;{\left(H_{st}^{\mu }\right)}_{e}({£}_{\sigma },\hbar_{\alpha })\leq {\left(H_{st}^{\mu }\right)}_{e}(\hbar_{\alpha }^{c},{£}_{\sigma }^{c}),$$

(10) $${\left(H_{st}^{\omega }\right)}_{e}({£}_{\sigma },\hbar_{\alpha })\leq {\left(H_{st}^{\omega }\right)}_{e}(\hbar_{\alpha }^{c}{£}_{\sigma }^{c}).$$

Based on (9) and (10), we have

$${\left(H_{st}^{\upsilon }\right)}_{e}({£}_{\sigma },\hbar_{\alpha })={\left(H_{st}^{\upsilon }\right)}_{e}(\hbar_{\alpha }^{c},{£}_{\sigma }^{c}),\;\;\;\;{\left(H_{st}^{\mu }\right)}_{e}({£}_{\sigma },\hbar_{\alpha })={\left(H_{st}^{\mu }\right)}_{e}(\hbar_{\alpha }^{c},{£}_{\sigma }^{c}),$$

$${\left(H_{st}^{\omega }\right)}_{e}({£}_{\sigma },\hbar_{\alpha })={\left(H_{st}^{\omega }\right)}_{e}(\hbar_{\alpha }^{c},{£}_{\sigma }^{c}).$$

(2) (i) straightforward.

(ii) Since ${\left(T_{H_{st}}^{\upsilon }\right)}_{e}({\overset{˜}{⋎}}^{\iota })={\left(H_{st}^{\upsilon }\right)}_{e}({\overset{˜}{⋎}}^{\iota },{\overset{˜}{⋎}}^{\iota })=1$, ${\left(T_{H_{st}}^{\mu }\right)}_{e}({\overset{˜}{⋎}}^{\iota })={\left(H_{st}^{\mu }\right)}_{e}({\overset{˜}{⋎}}^{\iota },{\overset{˜}{⋎}}^{\iota })=0$ and ${\left(T_{H_{st}}^{\omega }\right)}_{e}({\overset{˜}{⋎}}^{\iota })={\left(H_{st}^{\omega }\right)}_{e}({\overset{˜}{⋎}}^{\iota },{\overset{˜}{⋎}}^{\iota })=0$ for each ι∈ς we have ${\left(T_{H_{st}}^{\upsilon \mu \omega }\right)}_{⋎}$ is stratified which is finer than ${\left(T_{H}^{\upsilon \mu \omega }\right)}_{⋎}$. Thus, ${\left(T_{H_{st}}^{\upsilon \mu \omega }\right)}_{⋎}⊒{\left[{\left(T_{H}^{\upsilon \mu \omega }\right)}_{st}\right]}_{{}_{⋎}}$.

Conversely, suppose that ${£}_{\sigma }\in \overset{˜}{\left(¥,⋎\right)}$ such that

$${\left(T_{H_{st}}^{\upsilon }\right)}_{e}({£}_{\sigma })={\left(H_{st}^{\upsilon }\right)}_{e}({£}_{\sigma },{£}_{\sigma })≰{\left[{\left(T_{H}^{\upsilon }\right)}_{st}\right]}_{{}_{e}}({£}_{\sigma }),$$

(11) $${\left(T_{H_{st}}^{\mu }\right)}_{e}({£}_{\sigma })={\left(H_{st}^{\mu }\right)}_{e}({£}_{\sigma },{£}_{\sigma })≱{\left[{\left(T_{H}^{\mu }\right)}_{st}\right]}_{{}_{e}}({£}_{\sigma }),$$

$${\left(T_{H_{st}}^{\omega }\right)}_{e}({£}_{\sigma })={\left(H_{st}^{\omega }\right)}_{e}({£}_{\sigma },{£}_{\sigma })≱{\left[{\left(T_{H}^{\omega }\right)}_{st}\right]}_{{}_{e}}({£}_{\sigma }).$$

From the concept of ${\left(H_{st}^{\upsilon \mu \omega }\right)}_{⋎}$, there exists a collection

$$A=\{({\left({£}_{\sigma }\right)}_{j},{\left({£}_{\sigma }\right)}_{j},{\overset{˜}{⋎}}^{\iota_{j}})\}\in N({£}_{\sigma },{£}_{\sigma }),r\in \varsigma \;and\;j\in J$$

such that

$${\left[{\left(T_{H}^{\upsilon }\right)}_{st}\right]}_{{}_{e}}({£}_{\sigma })\leq r\leq \underset{\left({\left({£}_{{}_{A}}\right)}_{i},{\left({£}_{\sigma }\right)}_{i},{\overset{˜}{⋎}}^{\iota_{i}}\right)\in A}{⋀}H_{e}^{\upsilon }\left({\left({£}_{\sigma }\right)}_{i},{\left({£}_{\sigma }\right)}_{i}\right)=\underset{\left({\left({£}_{\sigma }\right)}_{i},{\left({£}_{\sigma }\right)}_{i},{\overset{˜}{⋎}}^{\iota_{i}}\right)\in A}{⋀}{\left(T_{H}^{\upsilon }\right)}_{e}\left({\left({£}_{\sigma }\right)}_{i}\right),$$

$${\left[{\left(T_{H}^{\mu }\right)}_{st}\right]}_{{}_{e}}({£}_{\sigma })\geq 1-r\geq \underset{\left({\left({£}_{\sigma }\right)}_{i},{\left({£}_{\sigma }\right)}_{i},{\overset{˜}{⋎}}^{\iota_{i}}\right)\in A}{⋁}H_{e}^{\mu }\left({\left({£}_{\sigma }\right)}_{i},{\left({£}_{\sigma }\right)}_{i}\right)=\underset{\left({\left({£}_{\sigma }\right)}_{i},{\left({£}_{\sigma }\right)}_{i},{\overset{˜}{⋎}}^{\iota_{i}}\right)\in A}{⋁}{\left(T_{H}^{\mu }\right)}_{e}\left({\left({£}_{\sigma }\right)}_{i}\right),$$

$${\left[{\left(T_{H}^{\omega }\right)}_{st}\right]}_{{}_{e}}({£}_{{}_{\sigma }})\geq 1-r\geq \underset{\left({\left({£}_{\sigma }\right)}_{i},{\left({£}_{\sigma }\right)}_{i},{\overset{˜}{⋎}}^{\iota_{i}}\right)\in A}{⋁}H_{e}^{\omega }\left({\left({£}_{\sigma }\right)}_{i},{\left({£}_{\sigma }\right)}_{i}\right)=\underset{\left({\left({£}_{\sigma }\right)}_{i},{\left({£}_{\sigma }\right)}_{i},{\overset{˜}{⋎}}^{\iota_{i}}\right)\in A}{⋁}{\left(T_{H}^{\omega }\right)}_{e}\left({\left({£}_{\sigma }\right)}_{i}\right).$$

On another side,

$${\left[{\left(T_{H}^{\upsilon }\right)}_{e}\right]}_{{}_{st}}({£}_{{}_{\sigma }})={\left[{\left(T_{H}^{\upsilon }\right)}_{st}\right]}_{{}_{e}}\left({⊔}_{j\in J}({\left({£}_{\sigma }\right)}_{j}⊓{\overset{˜}{⋎}}^{\iota_{{}_{j}}})\right)\geq \underset{j\in J}{⋀}{\left[{\left(T_{H}^{\upsilon }\right)}_{e}\right]}_{{}_{st}}\left({\left({£}_{\sigma }\right)}_{j}⊓{\overset{˜}{⋎}}^{\iota_{{}_{j}}}\right)\geq \underset{j\in J}{⋀}{\left[{\left(T_{H}^{\upsilon }\right)}_{e}\right]}_{{}_{st}}\left({\left({£}_{\sigma }\right)}_{j}\right)\wedge {\left[{\left(T_{H}^{\upsilon }\right)}_{st}\right]}_{{}_{e}}\left({\overset{˜}{⋎}}^{\iota_{{}_{j}}}\right)\geq \underset{\left({\left({£}_{{}_{A}}\right)}_{i},{\left({£}_{\sigma }\right)}_{i},{\overset{˜}{⋎}}^{\iota_{i}}\right)\in A}{⋀}{\left(T_{H}^{\upsilon }\right)}_{e}\left({\left({£}_{\sigma }\right)}_{i}\right)\geq r,$$

$${\left[{\left(T_{H}^{\mu }\right)}_{e}\right]}_{{}_{st}}({£}_{{}_{V}})={\left[{\left(T_{H}^{\mu }\right)}_{st}\right]}_{{}_{e}}\left({⊔}_{j\in J}({\left({£}_{\sigma }\right)}_{j}⊓{\overset{˜}{⋎}}^{1-\iota_{{}_{j}}})\right)\leq \underset{j\in J}{⋁}{\left[{\left(T_{H}^{\mu }\right)}_{e}\right]}_{{}_{st}}\left({\left({£}_{\sigma }\right)}_{j}⊓{\overset{˜}{⋎}}^{\iota_{{}_{j}}}\right)\leq \underset{j\in J}{⋁}{\left[{\left(T_{H}^{\mu }\right)}_{st}\right]}_{{}_{e}}\left({\left({£}_{\sigma }\right)}_{j}\right)\vee {\left[{\left(T_{H}^{\mu }\right)}_{st}\right]}_{{}_{e}}\left({\overset{˜}{⋎}}^{\iota_{{}_{j}}}\right)\leq \underset{\left({\left({£}_{\sigma }\right)}_{i},{\left({£}_{\sigma }\right)}_{i},{\overset{˜}{⋎}}^{\iota_{i}}\right)\in A}{⋁}{\left(T_{H}^{\mu }\right)}_{e}\left({\left({£}_{\sigma }\right)}_{i}\right)\leq 1-r,$$

$${\left[{\left(T_{H}^{\omega }\right)}_{e}\right]}_{{}_{st}}({£}_{\sigma })={\left[{\left(T_{H}^{\mu }\right)}_{st}\right]}_{{}_{e}}\left({⊔}_{j\in J}({\left({£}_{\sigma }\right)}_{j}⊓{\overset{˜}{⋎}}^{1-\iota_{{}_{j}}})\right)\leq \underset{j\in J}{⋁}{\left[{\left(T_{H}^{\omega }\right)}_{e}\right]}_{{}_{st}}\left({\left({£}_{\sigma }\right)}_{j}⊓{\overset{˜}{⋎}}^{\iota_{{}_{j}}}\right)\leq \underset{j\in J}{⋁}{\left[{\left(T_{H}^{\omega }\right)}_{st}\right]}_{{}_{e}}\left({\left({£}_{\sigma }\right)}_{j}\right)\vee {\left[{\left(T_{H}^{\omega }\right)}_{st}\right]}_{{}_{e}}\left({\overset{˜}{⋎}}^{\iota_{{}_{j}}}\right)\leq \underset{\left({\left({£}_{\sigma }\right)}_{i},{\left({£}_{\sigma }\right)}_{i},{\overset{˜}{\text{E}}}^{\iota_{i}}\right)\in A}{⋁}{\left(T_{H}^{\omega }\right)}_{e}\left({\left({£}_{\sigma }\right)}_{i}\right)\leq 1-r.$$

This contradicts the hypothesis in equation (11). Thus, ${\left(T_{H_{st}}^{\upsilon \mu \omega }\right)}_{⋎}={\left[{\left(T_{H}^{\upsilon \mu \omega }\right)}_{st}\right]}_{{}_{⋎}}$.

(3) (i) Straightforward.

(ii) Obvious. □

Definition 7 A mapping $Z^{\upsilon },Z^{\mu },Z^{\omega }:⋎\to \varsigma^{\overset{˜}{\left(¥,⋎\right)}}$ is called single-valued neutrosophic soft filter (*svns-filter*) on ¥. If the following criteria are met, ∀ e∈⋎ and ${£}_{\sigma },\hbar_{\alpha }\in \overset{˜}{\left(¥,⋎\right)}$:

($Z_{1}$) $Z_{e}^{\upsilon }(\Phi )=0,Z_{e}^{\mu }(\Phi )=1,Z_{e}^{\omega }(\Phi )=1$ and $Z_{e}^{\upsilon }(\overset{˜}{⋎})=1,Z_{e}^{\mu }(\overset{˜}{⋎})=0,Z_{e}^{\omega }(\overset{˜}{⋎})=0$,

($Z_{2}$) $Z_{e}^{\upsilon }({£}_{\sigma }⊓\hbar_{\alpha })\geq Z_{e}^{\upsilon }({£}_{\sigma })\wedge Z_{e}^{\upsilon }(\hbar_{\alpha })$, $Z_{e}^{\mu }({£}_{\sigma }⊓\hbar_{\alpha })\leq Z_{e}^{\mu }({£}_{\sigma })\vee Z_{e}^{\mu }(\hbar_{\alpha })$,

 $Z_{e}^{\omega }({£}_{\sigma }⊓\hbar_{\alpha })\leq Z_{e}^{\omega }({£}_{\sigma })\vee Z_{e}^{\omega }(\hbar_{\alpha })$,

($Z_{3}$) If ${£}_{\sigma }⊑\hbar_{{}_{B}}$, then $Z_{e}^{\upsilon }({£}_{\sigma })\leq Z_{e}^{\upsilon }(\hbar_{\alpha })$, $Z_{e}^{\mu }({£}_{\sigma })\geq Z_{e}^{\mu }(\hbar_{\alpha })$

 and $Z_{e}^{\omega }({£}_{\sigma })\geq Z_{e}^{\omega }(\hbar_{\alpha })$.

The *svns- filter* $Z_{\;}^{\upsilon \mu \omega }$ is called stratified iff the following condition is met.

($Z_{S}$) For every e∈⋎, ι∈ς and ${£}_{\sigma }\in \overset{˜}{\left(¥,⋎\right)}$, $Z_{e}^{\upsilon }({£}_{\sigma }⊓{\overset{˜}{⋎}}^{\iota })\geq Z_{e}^{\upsilon }({£}_{\sigma })\wedge (\iota )$, $Z_{e}^{\mu }({£}_{\sigma }⊓{\overset{˜}{⋎}}^{\iota })\leq Z_{e}^{\mu }({£}_{\sigma })\vee (\iota )$, and $Z_{e}^{\omega }({£}_{\sigma }⊓{\overset{˜}{⋎}}^{\iota })\leq Z_{e}^{\omega }({£}_{\sigma })\vee (\iota )$.

The pair $\left(¥,Z_{⋎}^{\upsilon \mu \omega }\right)$ is said to be *stratified svns-filters space*.

If $Z_{⋎}^{\upsilon \mu \omega }$ and $Z_{⋎}^{⋆\upsilon \mu \omega }$ are *svns-filters* on ¥, then $Z_{⋎}^{\pi \alpha \sigma }$ is finer than $Z_{⋎}^{⋆\upsilon \mu \omega }$ or ($Z_{⋎}^{⋆\upsilon \mu \omega }$ is coarser than $Z_{⋎}^{\upsilon \mu \omega }$) indicated by $Z_{⋎}^{\upsilon \mu \omega }⊒Z_{⋎}^{⋆\upsilon \mu \omega }$ if $Z_{e}^{\upsilon }({£}_{\sigma })\geq Z_{e}^{⋆\upsilon }({£}_{\sigma })$, $Z_{e}^{\mu }({£}_{\sigma })\leq Z_{e}^{⋆\mu }({£}_{\sigma }))$ and $Z_{e}^{\omega }({£}_{\sigma })\leq Z_{e}^{⋆\omega }({£}_{\sigma })$.

Theorem 8 *A consider that*$\left(¥,Z_{⋎}^{\upsilon \mu \omega }\right)$*is svns-filters space. Define the mapping*$Z_{st}^{\upsilon }:⋎\to \varsigma^{\overset{˜}{\left(¥,⋎\right)}}$*,*$Z_{st}^{\mu }:⋎\to \varsigma^{\overset{˜}{\left(¥,⋎\right)}}$*,*$Z_{st}^{\omega }:⋎\to \varsigma^{\overset{˜}{\left(¥,⋎\right)}}$*as next:* ∀ ${£}_{\sigma }\in \overset{˜}{\left(¥,⋎\right)}$*,* e∈⋎

$$⋁\left[\underset{j\in J}{⋀}Z_{e}^{\upsilon }({\left({£}_{\sigma }\right)}_{j})\wedge \iota_{j}|{⊔}_{i\in J}({\left({£}_{\sigma }\right)}_{j}⊓{\overset{˜}{⋎}}^{\iota_{{}_{j}}})⊑{£}_{\sigma }\right]={\left(Z_{st}^{\upsilon }\right)}_{e}({£}_{\sigma }),$$

$$⋀\left[\underset{j\in J}{⋁}Z_{e}^{\upsilon }({\left({£}_{\sigma }\right)}_{j})\vee \iota_{j}|{⊔}_{i\in J}({\left({£}_{\sigma }\right)}_{j}⊓{\overset{˜}{⋎}}^{\iota_{{}_{j}}})⊑{£}_{\sigma }\right]={\left(Z_{st}^{\mu }\right)}_{e}({£}_{\sigma }),$$

$$⋀\left[\underset{j\in J}{⋁}Z_{e}^{\omega }({\left({£}_{\sigma }\right)}_{j})\vee \iota_{j}|{⊔}_{i\in J}({\left({£}_{\sigma }\right)}_{j}⊓{\overset{˜}{⋎}}^{\iota_{{}_{j}}})⊑{£}_{\sigma }\right]={\left(Z_{st}^{\omega }\right)}_{e}({£}_{\sigma }),$$

*where* ⋁ *and* ⋀ *are taken over all collections* $\left\{({\left({£}_{\sigma }\right)}_{j},{\overset{˜}{⋎}}^{\iota_{j}})\right\}$ *for each* j∈J *and* Jisfinite) *with* ${£}_{\sigma }⊒{⊔}_{j\in J}({\left({£}_{\sigma }\right)}_{j}⊓{\overset{˜}{⋎}}^{\iota_{j}})$*. Then* ${\left(Z_{st}^{\upsilon \mu \omega }\right)}_{⋎}$ *is the coarsest* stratified svns-filter *on* ¥ *which is finer than* $Z_{⋎}^{\upsilon \mu \omega }$*. Also,* ${\left(Z_{st}^{\upsilon \mu \omega }\right)}_{⋎}$ *is the stratification of a* snvs-filter $Z_{⋎}^{\upsilon \mu \omega }$ *on* ¥*.*

Proof At initial, we will prove that ${\left(Z_{st}^{\upsilon \mu \omega }\right)}_{⋎}$ is *stratified snvs-filter*:

($Z_{1}$) For any ι∈ς, there are collections $\left\{\overset{˜}{⋎}\right\}$ and {Φ} with ${\overset{˜}{⋎}}^{\iota }={\overset{˜}{⋎}}^{\iota }⊓\overset{˜}{⋎}$. We have

$${\left(Z_{st}^{\upsilon }\right)}_{e}(\overset{˜}{⋎})\geq Z_{e}^{\upsilon }(\overset{˜}{⋎})\wedge 1=1,\;\;\;\;\;{\left(Z_{st}^{\mu }\right)}_{e}(\overset{˜}{⋎})\leq Z_{e}^{\mu }(\overset{˜}{⋎})\vee 0=0,$$

$${\left(Z_{⋎}^{\omega }\right)}_{st}(\overset{˜}{⋎})\leq Z_{⋎}^{\omega }(\overset{˜}{⋎})\vee 0=0,$$

$${\left(Z_{st}^{\upsilon }\right)}_{e}(\overset{˜}{\Phi })\geq Z_{e}^{\upsilon }(\overset{˜}{\Phi })\wedge 0=0,\;\;\;\;{\left(Z_{st}^{\mu }\right)}_{e}(\overset{˜}{\Phi })\leq Z_{e}^{\mu }(\overset{˜}{\Phi })\vee 1=1,$$

$${\left(Z_{\Phi }^{\omega }\right)}_{st}(\overset{˜}{\Phi })\leq Z_{⋎}^{\omega }(\Phi )\vee 1=1.$$

Hence, ${\left(Z_{st}^{\upsilon }\right)}_{e}(\overset{˜}{⋎})=1$, ${\left(Z_{st}^{\mu }\right)}_{e}(\overset{˜}{⋎})=0,{\left(Z_{st}^{\omega }\right)}_{e}(\overset{˜}{⋎})=0$ and ${\left(Z_{st}^{\upsilon }\right)}_{e}(\Phi )=0$, ${\left(Z_{st}^{\mu }\right)}_{e}(\Phi )=1$, ${\left(Z_{st}^{\omega }\right)}_{e}(\Phi )=1$.

($Z_{2}$) Let $D({£}_{\sigma })=\{\{({\left({£}_{\sigma }\right)}_{j},{\overset{˜}{⋎}}^{\iota_{j}})\}|{⊔}_{j\in J}({\left({£}_{\sigma }\right)}_{j}⊓{\overset{˜}{⋎}}^{\iota_{j}})⊑{£}_{\sigma }\}$ and $D(\hbar_{\alpha })=\{\{({\left(\hbar_{\alpha }\right)}_{i},{\overset{˜}{⋎}}^{\iota_{i}})\}|{⊔}_{i\in \Gamma }({\left(\hbar_{\alpha }\right)}_{i}⊓{\overset{˜}{⋎}}^{\iota_{i}})⊑\hbar_{\alpha }\}$ for all j∈J and i∈Γ. Then,

$${£}_{\sigma }⊓\hbar_{{}_{B}}⊒({⊔}_{j\in J}({\left({£}_{\sigma }\right)}_{j}⊓{\overset{˜}{⋎}}^{\iota_{j}}))⊓({⊔}_{i\in \Gamma }({\left(\hbar_{\alpha }\right)}_{i}⊓{\overset{˜}{⋎}}^{\iota_{i}}))={⊔}_{j\in J}{⊔}_{i\in \Gamma }(({\left({£}_{\sigma }\right)}_{j}⊓{\overset{˜}{⋎}}^{\iota_{j}})⊓({\left(\hbar_{\alpha }\right)}_{i}⊓{\overset{˜}{⋎}}^{\iota_{i}}))={⊔}_{j\in J}{⊔}_{i\in \Gamma }(({\left({£}_{\sigma }\right)}_{j}⊓{\left(\hbar_{\alpha }\right)}_{i})⊓({\overset{˜}{⋎}}^{\iota_{j}}⊓{\overset{˜}{⋎}}^{\iota_{i}}))={⊔}_{l\in j\cup \Gamma }({\left(g_{{}_{C}}\right)}_{l}⊓{\overset{˜}{⋎}}^{w_{{}_{l}}}),$$

where ${\overset{˜}{⋎}}^{w_{{}_{l}}}={\overset{˜}{⋎}}^{\iota_{{}_{j}}}⊓{\overset{˜}{⋎}}^{\iota_{{}_{i}}}$, $w_{{}_{l}}=\iota_{j}⊓\iota_{{}_{i}}$ and ${\left(g_{{}_{C}}\right)}_{l}={\left({£}_{\sigma }\right)}_{j}⊓{\left(\hbar_{\alpha }\right)}_{i}$, which implies

$${\left(Z_{st}^{\upsilon }\right)}_{e}({£}_{\sigma }⊓\hbar_{\alpha })\geq \underset{j\in J}{⋀}\left(Z_{e}^{\upsilon }({\left({£}_{\sigma }\right)}_{j}⊓{\left(\hbar_{\alpha }\right)}_{i})\right)\wedge w_{{}_{l}}\geq \underset{j}{⋀}(Z_{e}^{\upsilon }({\left({£}_{\sigma }\right)}_{j}))\wedge \underset{i}{⋀}(Z_{e}^{\upsilon }({\left(\hbar_{\alpha }\right)}_{i}))\wedge (\iota_{j}\wedge \iota_{i})\geq \left[\underset{j}{⋀}(Z_{e}^{\upsilon }({\left({£}_{\sigma }\right)}_{j})\wedge \iota_{j})\right]\wedge \left[\underset{i}{⋀}(Z_{e}^{\upsilon }({\left(\hbar_{\alpha }\right)}_{i})\wedge \iota_{i})\right]\geq {\left(Z_{st}^{\upsilon }\right)}_{e}({£}_{\sigma })\wedge {\left(Z_{st}^{\upsilon }\right)}_{e}(\hbar_{\alpha }),$$

$${\left(Z_{st}^{\mu }\right)}_{e}({£}_{\sigma }⊓\hbar_{\alpha })\leq \underset{j\in J}{⋁}\left(Z_{e}^{\mu }({\left({£}_{\sigma }\right)}_{j}⊓{\left(\hbar_{\alpha }\right)}_{i})\right)\vee w_{l}\leq \underset{j}{⋁}(Z_{e}^{\mu }({\left({£}_{{}_{A}}\right)}_{j}))\vee \underset{i}{⋁}(Z_{e}^{\mu }({\left(\hbar_{\alpha }\right)}_{i}))\vee (\iota_{j}\wedge \iota_{i})\leq \left[\underset{j}{⋁}(Z_{e}^{\mu }({\left({£}_{\sigma }\right)}_{j})\vee \iota_{j})\right]\vee \left[\underset{i}{⋁}(Z_{e}^{\mu }({\left(\hbar_{\alpha }\right)}_{i})\vee \iota_{i})\right]\geq {\left(Z_{st}^{\mu }\right)}_{e}({£}_{\sigma })\vee {\left(Z_{st}^{\mu }\right)}_{e}(\hbar_{\alpha }),$$

$${\left(Z_{st}^{\omega }\right)}_{e}({£}_{\sigma }⊓\hbar_{\alpha })\leq \underset{j\in J}{⋁}\left(Z_{e}^{om}({\left({£}_{\sigma }\right)}_{j}⊓{\left(\hbar_{\alpha }\right)}_{i})\right)\vee w_{l}\leq \left[\underset{j}{⋁}(Z_{e}^{\omega }({\left({£}_{\sigma }\right)}_{j}))\right]\vee \left[\underset{i}{⋁}(Z_{e}^{\omega }({\left(\hbar_{\alpha }\right)}_{i}))\vee (\iota_{j}\wedge \iota_{i})\right]\leq \left[\underset{j}{⋁}(Z_{e}^{\omega }({\left({£}_{\sigma }\right)}_{j})\vee \iota_{j})\right]\vee \left[\underset{i}{⋁}(Z_{e}^{\omega }({\left(\hbar_{\alpha }\right)}_{i})\vee \iota_{i})\right]\geq {\left(Z_{st}^{\omega }\right)}_{e}({£}_{\sigma })\vee {\left(Z_{st}^{\omega }\right)}_{e}(\hbar_{\alpha }).$$

Thus, the proof of ($Z_{2}$) is complete.

($Z_{3}$) Straightforward.

($Z_{S}$) Suppose

$$D({£}_{\sigma })=\{\{({\left({£}_{\sigma }\right)}_{j},{\overset{˜}{⋎}}^{\iota_{j}})|{£}_{\sigma }⊒{⊔}_{j\in J}({\left({£}_{\sigma }\right)}_{j}⊓{\overset{˜}{⋎}}^{\iota_{j}})\},\;for\;all\;j\in J,$$

$${\left(Z_{st}^{\upsilon }\right)}_{e}({£}_{\sigma }⊓{\overset{˜}{⋎}}^{\iota })\geq \left[\underset{j\in J}{⋀}(Z_{e}^{\upsilon }({\left({£}_{{}_{\sigma }}\right)}_{j})\wedge {\overset{˜}{⋎}}^{\iota })\wedge \iota_{j}\right]\geq \left[\underset{j\in J}{⋀}(Z_{e}^{\upsilon }({\left({£}_{\sigma }\right)}_{j})\wedge \iota )\wedge \iota_{j}\right]\geq \left[\underset{j\in J}{⋀}Z_{e}^{\upsilon }({\left({£}_{\sigma }\right)}_{j})\wedge \iota_{j}\right]\wedge (\iota )\geq {\left(Z_{st}^{\upsilon }\right)}_{e}({£}_{\sigma })\wedge (\iota ),$$

$${\left(Z_{st}^{\mu }\right)}_{e}({£}_{\sigma }⊓{\overset{˜}{⋎}}^{\iota })\leq \left[\underset{j}{⋁}(Z_{e}^{\mu }({\left({£}_{\sigma }\right)}_{j})\vee {\overset{˜}{⋎}}^{\iota })\vee \iota_{j}\right]\leq \left[\underset{j}{⋁}(Z_{e}^{\mu }({\left({£}_{\sigma }\right)}_{j})\vee \iota )\vee \iota_{j}\right]\leq \left[\underset{j}{⋁}Z_{e}^{\mu }({\left({£}_{\sigma }\right)}_{j})\vee \iota_{j}\right]\vee (\iota )\leq {\left(Z_{st}^{\mu }\right)}_{e}({£}_{\sigma })\vee (\iota ).$$

Similarly, by using an analogous line of reasoning, we can show that ${\left(Z_{st}^{\omega }\right)}_{e}({£}_{\sigma }⊓{\overset{˜}{⋎}}^{\iota })\leq {\left(Z_{st}^{\omega }\right)}_{e}({£}_{\sigma })\vee (\iota )$. Thus, the proof of ($Z_{S}$) is complete.

Secondly, for every ${£}_{\sigma }\in \overset{˜}{\left(¥,⋎\right)}$, e∈⋎, there exist collections $\left\{\overset{˜}{⋎}\right\}$ with ${£}_{\sigma }={£}_{{}_{A}}⊓\overset{˜}{⋎}$. Then, ${\left(Z_{st}^{\upsilon }\right)}_{e}({£}_{\sigma })\geq Z_{e}^{\upsilon }({£}_{\sigma })$, ${\left(Z_{st}^{\mu }\right)}_{e}({£}_{\sigma })\leq Z_{e}^{\mu }({£}_{\sigma }))$ and ${\left(Z_{st}^{\omega }\right)}_{e}({£}_{\sigma })\leq Z_{e}^{\omega }({£}_{\sigma })$. Thus, ${\left(Z_{st}^{\upsilon \mu \omega }\right)}_{⋎}$ is finer than $Z_{⋎}^{\upsilon \mu \omega }$.

Finally, let ${\left(Z_{⋆st}^{\upsilon },Z_{⋆st}^{\mu },Z_{⋆st}^{\omega }\right)}_{⋎}$ be *stratified snvs-filter* which is finer than ${\left(Z^{\upsilon },Z^{\mu },Z^{\omega }\right)}_{⋎}$ on ¥ and

$$D({£}_{\sigma })=\{\{({\left({£}_{\sigma }\right)}_{j},{\overset{˜}{⋎}}^{\iota_{j}})|{£}_{\sigma }⊒{⊔}_{j\in J}({\left({£}_{\sigma }\right)}_{j}⊓{\overset{˜}{⋎}}^{\iota_{j}})\},\forall j\in J,$$

then we have,

$${\left(Z_{⋆st}^{\upsilon }\right)}_{e}({£}_{\sigma })\geq {\left(Z_{⋆st}^{\upsilon }\right)}_{e}({⊔}_{j\in J}({\left({£}_{\sigma }\right)}_{j}⊓{\overset{˜}{⋎}}^{\iota_{j}}))\geq \underset{j\in J}{⋀}{\left(Z_{⋆st}^{\upsilon }\right)}_{e}\left({\left({£}_{\sigma }\right)}_{j}⊓{\overset{˜}{⋎}}^{\iota_{j}}\right)\geq \underset{j\in J}{⋀}\left({\left(Z_{⋆st}^{\upsilon }\right)}_{e}({\left({£}_{\sigma }\right)}_{j})\wedge \iota_{j}\right)\geq \underset{\left({\left({£}_{\sigma }\right)}_{k},{\overset{˜}{⋎}}^{\iota_{k}}\right)\in \left\{{\left({£}_{\sigma }\right)}_{j},{\overset{˜}{⋎}}^{\iota_{j}}|j\in J\right\}}{⋀}Z_{e}^{\upsilon }({\left({£}_{\sigma }\right)}_{p}\wedge \iota_{k})\geq {\left(Z_{st}^{\upsilon }\right)}_{e}({£}_{\sigma }).$$

Likewise, using related reasoning, we can determine that ${\left(Z_{⋆st}^{\mu }\right)}_{e}({£}_{\sigma })\leq {\left(Z_{st}^{\mu }\right)}_{e}({£}_{\sigma })$ and ${\left(Z_{⋆st}^{\omega }\right)}_{e}({£}_{\sigma })\leq {\left(Z_{st}^{\omega }\right)}_{e}({£}_{\sigma })$. Hence ${\left(Z_{st}^{\upsilon \mu \omega }\right)}_{⋎}$ is the coarsest *stratified snvs-filter* finer than $Z_{⋎}^{\upsilon \mu \omega }$. □

Theorem 9 *Let*$\Theta (\overset{˜}{\left(¥,⋎\right)})$*and*$\Omega (\overset{˜}{\left(¥,⋎\right)})$*be collections of all* svns-filters *and,* svns-topogenous *correspondingly. Define,* ∀*,* ${£}_{\sigma },\hbar_{\alpha }\in \overset{˜}{\left(¥,⋎\right)}$*,* e∈⋎*,*

$$O_{e}^{\upsilon }(H,Z)({£}_{\sigma })=\underset{\hbar_{\alpha }\in \overset{˜}{\left(¥,⋎\right)}}{⋁}\{H_{e}^{\upsilon }(\hbar_{\alpha },{£}_{\sigma })\wedge Z_{e}^{\upsilon }({£}_{\sigma })\}$$

$$O_{e}^{\mu }(H,Z)({£}_{\sigma })=\underset{\hbar_{\alpha }\in \overset{˜}{\left(¥,⋎\right)}}{⋀}\{H_{e}^{\mu }(\hbar_{\alpha },{£}_{\sigma })\vee Z_{e}^{\mu }({£}_{\sigma })\}$$

$$O_{e}^{\omega }(H,Z)({£}_{\sigma })=\underset{\hbar_{\alpha }\in \overset{˜}{\left(¥,⋎\right)}}{⋀}\{H_{e}^{\omega }(\hbar_{\alpha },{£}_{\sigma })\vee Z_{e}^{\omega }({£}_{\sigma })\}$$

*where* $H_{⋎}^{\upsilon \mu \omega }\in \Omega (\overset{˜}{(¥,⋎}))$ *and* $Z_{⋎}^{\upsilon \mu \omega }\in \Theta (\overset{˜}{\left(¥,⋎\right)})$*. Then:*

*(1)* $O_{⋎}^{\upsilon \mu \omega }(H,Z)\in \Theta (\overset{˜}{\left(¥,⋎\right)})$ *.*

*(2)* $O_{⋎}^{\upsilon \mu \omega }(H,Z)⊑Z_{⋎}^{\upsilon \mu \omega }$ *for all* $Z_{\text{E}}^{\upsilon \mu \omega }\in \Theta (\overset{˜}{\left(¥,⋎\right)})$ *.*

*(3)* $O_{⋎}^{\upsilon \mu \omega }(H,H_{{£}_{\sigma }})={\left(H_{{£}_{\sigma }}^{\upsilon \mu \omega }\right)}_{⋎}$ *.*

*(4)* $O_{⋎}^{\upsilon \mu \omega }(H_{st},Z_{st})=[{\left(O_{st}^{\upsilon \mu \omega }\right]}_{{}_{⋎}}(H,Z)$ *.*

Proof ($Z_{1}$) Since $Z_{e}^{\upsilon }(\Phi )=0,Z_{e}^{\mu }(\Phi )=1,Z_{e}^{\omega }(\Phi )=1$ and $Z_{e}^{\upsilon }(\overset{˜}{⋎})=1,Z_{e}^{\mu }(\overset{˜}{⋎})=0,Z_{e}^{\omega }(\overset{˜}{⋎})=0$, we obtain; ∀ e∈⋎,

$$O_{e}^{\upsilon }(H,Z)(\Phi )=\underset{g_{{}_{C}}\in \overset{˜}{\left(¥,⋎\right)}}{⋁}\{H_{e}^{\upsilon }(g_{{}_{C}},\Phi )\wedge Z_{e}^{\upsilon }(\Phi )\},$$

$$O_{e}^{\mu }(H,Z)(\Phi )=\underset{g_{{}_{C}}\in \overset{˜}{\left(¥,⋎\right)}}{⋀}\{H_{e}^{\mu }(g_{{}_{C}},\Phi )\vee Z_{e}^{\mu }(\Phi )\},$$

$$O_{e}^{\omega }(H,Z)(\Phi )=\underset{g_{{}_{C}}\in \overset{˜}{\left(¥,⋎\right)}}{⋀}\{H_{e}^{\omega }(g_{{}_{C}},\Phi )\vee Z_{e}^{\omega }(\Phi )\},$$

thus, $O_{e}^{\upsilon }(H,Z)(\Phi )=0,O_{e}^{\mu }(H,Z)(\Phi )=1,O_{e}^{\omega }(H,Z)(\Phi )=1$ and $O_{e}^{\upsilon }(H,Z)(\overset{˜}{⋎})=1,O_{e}^{\mu }(H,Z)(\overset{˜}{⋎})=0,O_{e}^{\omega }(H,Z)(\overset{˜}{⋎})=0$.

($Z_{2}$) Let ${£}_{\sigma },\hbar_{\alpha }\in \overset{˜}{\left(¥,⋎\right)}$. Then we obtain

$$O_{e}^{\upsilon }(H,Z)({£}_{\sigma }⊓\hbar_{\alpha })=\underset{g_{{}_{C}}\in \overset{˜}{\left(¥,⋎\right)}}{⋁}\{H_{e}^{\upsilon }(g_{{}_{C}},{£}_{\sigma }⊓\hbar_{\alpha })\wedge Z_{e}^{\upsilon }({£}_{\sigma }⊓\hbar_{\alpha })\}\geq \underset{g_{{}_{C}}\in \overset{˜}{\left(¥,⋎\right)}}{⋁}\{(H_{e}^{\upsilon }(g_{{}_{C}},{£}_{\sigma })\wedge H_{e}^{\upsilon }(g_{{}_{C}},\hbar_{\alpha }))\wedge (Z_{e}^{\upsilon }({£}_{\sigma })\wedge Z_{e}^{\upsilon }(\hbar_{\alpha }))\}\geq \underset{g_{{}_{C}}\in \overset{˜}{\left(¥,⋎\right)}}{⋁}\{(H_{e}^{\upsilon }(g_{{}_{C}},{£}_{\sigma })\wedge Z_{e}^{\upsilon }({£}_{\sigma }))\}\wedge \underset{g_{{}_{C}}\in \overset{˜}{\left(¥,⋎\right)}}{⋁}\{(H_{e}^{\upsilon }(g_{{}_{C}},\hbar_{\alpha })\wedge Z_{e}^{\upsilon }(\hbar_{\alpha }))\}\geq O_{e}^{\upsilon }(H,Z)({£}_{\sigma })\wedge O_{e}^{\upsilon }(H,Z)(\hbar_{\alpha }),$$

$$O_{e}^{\mu }(H,Z)({£}_{\sigma }⊓\hbar_{\alpha })=\underset{g_{{}_{C}}\in \overset{˜}{\left(¥,⋎\right)}}{⋀}\{H_{e}^{\mu }(g_{{}_{C}},{£}_{\sigma }⊓\hbar_{\alpha })\vee Z_{e}^{\mu }({£}_{\sigma }⊓\hbar_{\alpha })\}\leq \underset{g_{{}_{C}}\in \overset{˜}{\left(¥,⋎\right)}}{⋀}\{(H_{e}^{\mu }(g_{{}_{C}},{£}_{\sigma })\vee H_{e}^{\mu }(g_{{}_{C}},\hbar_{\alpha }))\vee (Z_{e}^{\mu }({£}_{\sigma })\vee Z_{e}^{\mu }(\hbar_{\alpha }))\}\leq \underset{g_{{}_{C}}\in \overset{˜}{\left(¥,⋎\right)}}{⋀}\{(H_{e}^{\mu }(g_{{}_{C}},{£}_{\sigma })\vee Z_{e}^{\mu }({£}_{\sigma }))\}\vee \underset{g_{{}_{C}}\in \overset{˜}{\left(¥,⋎\right)}}{⋀}\{(H_{e}^{\mu }(g_{{}_{C}},\hbar_{{}_{\alpha }})\vee Z_{e}^{\mu }(\hbar_{\alpha }))\}\leq O_{e}^{\mu }(H,Z)({£}_{\sigma })\vee O_{e}^{\mu }(H,Z)(\hbar_{\alpha }).$$

Likewise, using related reasoning, we can determine that

$$O_{e}^{\omega }(H,Z)({£}_{\sigma }⊓\hbar_{\alpha })\leq O_{e}^{\omega }(H,Z)({£}_{\sigma })\vee O_{e}^{\omega }(H,Z)(\hbar_{\alpha }).$$

($Z_{3}$) If ${£}_{\sigma }⊑\hbar_{\alpha }$, then;

$$O_{e}^{\upsilon }(H,Z)({£}_{\sigma })=\underset{g_{{}_{C}}\in \overset{˜}{\left(¥,⋎\right)}}{⋁}\{H_{e}^{\upsilon }(g_{{}_{C}},{£}_{\sigma })\wedge Z_{e}^{\upsilon }({£}_{{}_{\sigma }})\}\leq \underset{g_{{}_{C}}\in \overset{˜}{\left(¥,⋎\right)}}{⋁}\{H_{e}^{\upsilon }(g_{{}_{C}},\hbar_{\alpha })\wedge Z_{e}^{\upsilon }(\hbar_{\alpha })\}=O_{e}^{\upsilon }(H,Z)(\hbar_{\alpha }),$$

$$O_{e}^{\mu }(H,Z)({£}_{\sigma })=\underset{g_{{}_{C}}\in \overset{˜}{\left(¥,⋎\right)}}{⋀}\{H_{e}^{\mu }(g_{{}_{C}},{£}_{\sigma })\vee Z_{e}^{\mu }({£}_{\sigma })\}\geq \underset{g_{{}_{C}}\in \overset{˜}{\left(¥,⋎\right)}}{⋀}\{H_{e}^{\mu }(g_{{}_{C}},\hbar_{\alpha })\vee Z_{e}^{\mu }(\hbar_{\alpha })\}=O_{e}^{\mu }(H,Z)(\hbar_{\alpha }),$$

$$O_{e}^{\omega }(H,Z)({£}_{\sigma })=\underset{g_{{}_{C}}\in \overset{˜}{\left(¥,⋎\right)}}{⋀}\{H_{e}^{\omega }(g_{{}_{C}},{£}_{\sigma })\vee Z_{e}^{\omega }({£}_{\sigma })\}\geq \underset{g_{{}_{C}}\in \overset{˜}{\left(¥,⋎\right)}}{⋀}\{H_{e}^{\omega }(g_{{}_{C}},\hbar_{\alpha })\vee Z_{e}^{\mu }(\hbar_{\alpha })\}=O_{e}^{\mu }(H,Z)(\hbar_{\alpha }).$$

(2) It is obvious from the definition.

(3) From (2), we obtain $O_{⋎}^{\upsilon \mu \omega }(H,H_{{£}_{\sigma }})⊑{\left(H_{{£}_{\sigma }}^{\upsilon \mu \omega }\right)}_{\text{E}}$. Now we just need to prove that $O_{⋎}^{\upsilon \mu \omega }(H,H_{{£}_{\sigma }})⊒{\left(H_{{£}_{\sigma }}^{\upsilon \mu \omega }\right)}_{⋎}$. Let $\Phi \neq \hbar_{\alpha }\in \overset{˜}{\left(¥,¥\right)}$. Then we obtain

$$O_{⋎}^{\upsilon }(H,H_{{£}_{\sigma }})(\hbar_{\alpha })=\underset{g_{{}_{C}}\in \overset{˜}{\left(¥,⋎\right)}}{⋁}\{H_{e}^{\upsilon }(g_{{}_{C}},\hbar_{\alpha })\wedge {\left(H_{{£}_{\sigma }}^{\upsilon }\right)}_{e}(\hbar_{\alpha })\}=\underset{g_{{}_{C}}\in \overset{˜}{\left(¥,⋎\right)}}{⋁}\{H_{e}^{\upsilon }(g_{{}_{C}},\hbar_{\alpha })\wedge H_{e}^{\upsilon }({£}_{\sigma },\hbar_{\alpha })\}\geq H_{e}^{\upsilon }({£}_{\sigma },\hbar_{\alpha })\wedge H_{e}^{\upsilon }({£}_{\sigma },\hbar_{\alpha })=H_{e}^{\upsilon }({£}_{\sigma },\hbar_{\alpha })={\left(H_{{£}_{\sigma }}^{\upsilon }\right)}_{e}(\hbar_{\alpha }).$$

Similarly, we can establish through a parallel argument that

$$O_{⋎}^{\mu }(H,H_{{£}_{\sigma }})(\hbar_{\alpha })\geq {\left(H_{{£}_{\sigma }}^{\mu }\right)}_{e}(\hbar_{\alpha }),\;\;\;O_{⋎}^{\omega }(H,H_{{£}_{\sigma }})(\hbar_{\alpha })\geq {\left(H_{{£}_{\sigma }}^{\omega }\right)}_{e}(\hbar_{\alpha })$$

(4) Theorem 5, Theorem 8 provide a clear and simple explanation. □

## 4 Stratified single-valued neutrosophic soft quasi-proximity

Definition 8 A mapping $Q^{\upsilon },Q^{\mu },Q^{\omega }:⋎\to \varsigma^{\overset{˜}{\left(¥,⋎\right)}\times \overset{˜}{\left(¥,⋎\right)}}$ is said to be *svnsq-proximity* on ¥ if the following criteria are met, ∀ e∈⋎, ${£}_{A},\hbar_{B}\in \overset{˜}{\left(¥,⋎\right)}$:

($Q_{1}$) $Q_{e}^{\upsilon }(\overset{˜}{⋎},\Phi )=Q_{e}^{\upsilon }(\Phi ,\overset{˜}{⋎})=0$, $Q_{e}^{\mu }(\overset{˜}{⋎},\Phi )=Q_{e}^{\mu }(\Phi ,\overset{˜}{⋎})=1$, $Q_{e}^{\omega }(\overset{˜}{⋎},\Phi )=Q_{e}^{\omega }(\Phi ,\overset{˜}{⋎})=$

 1.

($Q_{2}$) If $Q_{e}^{\upsilon }({£}_{\sigma },\hbar_{\alpha })\neq 1$, $Q_{e}^{\mu }({£}_{\sigma },\hbar_{\alpha })\neq 0$ and $Q_{e}^{\omega }({£}_{\sigma },\hbar_{\alpha })\neq 0$, then ${£}_{\sigma }⊑\hbar_{\alpha }^{c}$.

($Q_{3}$) If ${£}_{\sigma }⊑\hbar_{\alpha }$, then $Q_{e}^{\upsilon }({£}_{\sigma },g_{{}_{C}})\leq Q_{e}^{\upsilon }(\hbar_{\alpha },g_{{}_{C}})$, $Q_{e}^{\mu }({£}_{\sigma },g_{{}_{C}})\geq Q_{e}^{\mu }(\hbar_{\alpha },g_{{}_{C}})$,

 $Q_{e}^{\omega }({£}_{\sigma },g_{{}_{C}})\geq Q_{e}^{\omega }(\hbar_{\alpha },g_{{}_{C}})$ for any ${£}_{A},\hbar_{\alpha },g_{{}_{C}}\in \overset{˜}{\left(¥,⋎\right)}$.

($Q_{4}$) $Q_{e}^{\upsilon }({\left({£}_{\sigma }\right)}_{1}⊓{\left({£}_{\sigma }\right)}_{2},{\left(\hbar_{\alpha }\right)}_{1}⊔{\left(\hbar_{\alpha }\right)}_{2})\leq Q_{e}^{\upsilon }({\left({£}_{\sigma }\right)}_{1}⊓{\left(\hbar_{\alpha }\right)}_{1})\wedge Q_{e}^{\upsilon }({\left({£}_{\sigma }\right)}_{2}⊔{\left(\hbar_{\alpha }\right)}_{2})$,

 $Q_{e}^{\mu }({\left({£}_{\sigma }\right)}_{1}⊓{\left({£}_{\sigma }\right)}_{2},{\left(\hbar_{\alpha }\right)}_{1}⊔{\left(\hbar_{\alpha }\right)}_{2})\geq Q_{e}^{\mu }({\left({£}_{\sigma }\right)}_{1}⊓{\left(\hbar_{\alpha }\right)}_{1})\vee Q_{e}^{\mu }({\left({£}_{\sigma }\right)}_{2}⊔{\left(\hbar_{\alpha }\right)}_{2})$,

 $Q_{e}^{\omega }({\left({£}_{\sigma }\right)}_{1}⊓{\left({£}_{\sigma }\right)}_{2},{\left(\hbar_{\alpha }\right)}_{1}⊔{\left(\hbar_{\alpha }\right)}_{2})\geq Q_{e}^{\omega }({\left({£}_{\sigma }\right)}_{1}⊓{\left(\hbar_{\alpha }\right)}_{1})\vee Q_{e}^{\omega }({\left({£}_{\sigma }\right)}_{2}⊔{\left(\hbar_{\alpha }\right)}_{2})$.

($Q_{5}$) $Q_{e}^{\upsilon }({£}_{\sigma },\hbar_{\alpha })\geq \underset{g_{{}_{C}}\in \overset{˜}{(¥},⋎)}{⋀}\left[Q_{e}^{\upsilon }({£}_{\sigma },g_{{}_{C}})\vee Q_{e}^{\upsilon }(g_{{}_{C}}^{c},\hbar_{\alpha })\right]$,

 $Q_{e}^{\mu }({£}_{\sigma },\hbar_{\alpha })\leq \underset{g_{{}_{C}}\in \overset{˜}{\left(¥,⋎\right)}}{⋁}\left[Q_{e}^{\mu }({£}_{\sigma },g_{{}_{C}})\wedge Q_{e}^{\mu }(g_{{}_{C}}^{c},\hbar_{\alpha })\right]$,

 $Q_{e}^{\omega }({£}_{A},\hbar_{\alpha })\leq \underset{g_{{}_{C}}\in \overset{˜}{\left(¥,⋎\right)}}{⋁}\left[Q_{e}^{\omega }({£}_{\sigma },g_{{}_{C}})\wedge Q_{e}^{\omega }(g_{{}_{C}}^{c},\hbar_{\alpha })\right]$.

The *svnsq-proximity* $Q_{⋎}^{\upsilon \mu \omega }$ is called stratified iff the following condition is met.

($Q_{S}$) $Q_{e}^{\upsilon }({\overset{˜}{⋎}}^{\iota },{\overset{˜}{⋎}}^{1-\iota })=0$, $Q_{e}^{\mu }({\overset{˜}{⋎}}^{\iota },{\overset{˜}{⋎}}^{1-\iota })=Q_{e}^{\omega }({\overset{˜}{⋎}}^{\iota },{\overset{˜}{⋎}}^{1-\iota })=1$, ∀ ι∈ς.

In this case, a pair $\left(¥,Q_{⋎}^{\upsilon \mu \omega }\right)$ is said to be *stratified svnsq-proximity space*.

Let ${\left(Q_{⋎}^{\upsilon \mu \omega }\right)}_{1}$ and ${\left(Q_{⋎}^{\upsilon \mu \omega }\right)}_{2}$ be *svnsq-proximity* on ⋎. We say ${\left(Q_{⋎}^{\upsilon \mu \omega }\right)}_{1}$ is finer than ${\left(Q_{⋎}^{\upsilon \mu \omega }\right)}_{2}$[${\left(Q_{⋎}^{\upsilon \mu \omega }\right)}_{2}$ is coarser than ${\left(Q_{⋎}^{\upsilon \mu \omega }\right)}_{1}$] if ${\left(Q_{e}^{\upsilon }\right)}_{2}({£}_{\sigma },\hbar_{\alpha })\geq {\left(Q_{e}^{\upsilon }\right)}_{1}({£}_{\sigma },\hbar_{\alpha })$, ${\left(Q_{e}^{\mu }\right)}_{2}({£}_{\sigma },\hbar_{\alpha })\leq {\left(Q_{e}^{\mu }\right)}_{1}({£}_{\sigma },\hbar_{\alpha })$, ${\left(Q_{e}^{\omega }\right)}_{2}({£}_{\sigma },\hbar_{\alpha })\leq {\left(Q_{e}^{\omega }\right)}_{1}({£}_{\sigma },\hbar_{\alpha })$.

Definition 9 Let $\left(¥,{\left(Q_{⋎}^{\upsilon \mu \omega }\right)}_{1}\right)$ and $\left(U,{\left(Q_{R}^{\upsilon \mu \omega }\right)}_{2}\right)$ be *svnsq-proximity spaces*. A mapping $\psi_{{}_{\varphi }}:(¥,{\left(Q_{⋎}^{\upsilon \mu \omega }\right)}_{1})\to (U,{\left(Q_{R}^{\upsilon \mu \omega }\right)}_{2})$ is called *svnsq-proximity continuous* iff

$${\left(Q_{e}^{\upsilon }\right)}_{1}(\psi_{{}_{\varphi }}^{-1}(\hbar_{\alpha }),\psi_{{}_{\varphi }}^{-1}(g_{{}_{C}}))\geq {\left(Q_{\varphi (e)}^{\upsilon }\right)}_{2}(\hbar_{\alpha },g_{{}_{c}}),$$

$${\left(Q_{e}^{\mu }\right)}_{1}(\psi_{{}_{\varphi }}^{-1}(\hbar_{\alpha }),\psi_{{}_{\varphi }}^{-1}(g_{{}_{C}}))\leq {\left(Q_{\varphi (e)}^{\mu }\right)}_{2}(\hbar_{\alpha },g_{{}_{c}})$$

$${\left(Q_{e}^{\omega }\right)}_{1}(\psi_{{}_{\varphi }}^{-1}(\hbar_{\alpha }),\psi_{{}_{\varphi }}^{-1}(g_{{}_{C}}))\leq {\left(Q_{\varphi (e)}^{\omega }\right)}_{2}(\hbar_{\alpha },g_{{}_{c}}),$$

for any $g_{{}_{c}},\hbar_{\alpha }\in \overset{˜}{\left(U,R\right)}$, e∈⋎.

Theorem 10 *Let*$\left(¥,Q_{⋎}^{\upsilon \mu \omega }\right)$*be* svnsq-proximity space*. For each* e∈⋎*, define*

$${\left(Q_{st}^{\upsilon }\right)}_{e}({£}_{\sigma },\hbar_{\alpha })=\underset{\left\{{\left({£}_{\sigma }\right)}_{j},{\left(\hbar_{\alpha }\right)}_{j},{\overset{˜}{⋎}}^{\iota_{j}}|j\in J\}\in D({£}_{\sigma },\hbar_{\alpha })\right\}}{⋀}\left\{\underset{\left({\left({£}_{\sigma }\right)}_{i},{\left(\hbar_{\alpha }\right)}_{i},{\overset{˜}{⋎}}^{\iota_{i}}\right)\in \left\{{\left({£}_{\sigma }\right)}_{j},{\left(\hbar_{\alpha }\right)}_{j},{\overset{˜}{⋎}}^{\iota_{j}}|j\in J\right\}}{⋁}Q_{e}^{\upsilon }({\left({£}_{\sigma }\right)}_{i},{\left(\hbar_{\alpha }\right)}_{i})\right\},$$

$${\left(Q_{st}^{\mu }\right)}_{e}({£}_{\sigma },\hbar_{\alpha })=\underset{\left\{{\left({£}_{\sigma }\right)}_{j},{\left(\hbar_{\alpha }\right)}_{j},{\overset{˜}{⋎}}^{\iota_{j}}|j\in J\}\in D({£}_{\sigma },\hbar_{\alpha })\right\}}{⋁}\left\{\underset{\left({\left({£}_{\sigma }\right)}_{i},{\left(\hbar_{\alpha }\right)}_{i},{\overset{˜}{⋎}}^{\iota_{i}}\right)\in \left\{{\left({£}_{\sigma }\right)}_{j},{\left(\hbar_{\alpha }\right)}_{j},{\overset{˜}{⋎}}^{\iota_{j}}|j\in J\right\}}{⋀}Q_{e}^{\mu }({\left({£}_{\sigma }\right)}_{i},{\left(\hbar_{\alpha }\right)}_{i})\right\},$$

$${\left(Q_{st}^{\omega }\right)}_{e}({£}_{\sigma },\hbar_{\alpha })=\underset{\left\{{\left({£}_{\sigma }\right)}_{j},{\left(\hbar_{\alpha }\right)}_{j},{\overset{˜}{⋎}}^{\iota_{j}}|j\in J\}\in D({£}_{\sigma },\hbar_{\alpha })\right\}}{⋁}\left\{\underset{\left({\left({£}_{\sigma }\right)}_{i},{\left(\hbar_{\alpha }\right)}_{i},{\overset{˜}{⋎}}^{\iota_{i}}\right)\in \left\{{\left({£}_{\sigma }\right)}_{j},{\left(\hbar_{\alpha }\right)}_{j},{\overset{˜}{⋎}}^{\iota_{j}}|j\in J\right\}}{⋀}Q_{e}^{\omega }({\left({£}_{\sigma }\right)}_{i},{\left(\hbar_{\alpha }\right)}_{i})\right\},$$

*where* $D({£}_{\sigma },\hbar_{\alpha })=\{\{({\left({£}_{\sigma }\right)}_{j},{\left(\hbar_{\alpha }\right)}_{j},{\overset{˜}{\text{E}}}^{\iota_{j}})|j\in J,J\;is\;finite)\}|{£}_{\sigma }⊑{⊔}_{j\in J}({\left({£}_{\sigma }\right)}_{j}⊓{\overset{˜}{\text{E}}}^{\iota_{j}})\;and\;\hbar_{\alpha }⊑{⊓}_{j\in J}({\left(\hbar_{\alpha }\right)}_{j}⊔{\overset{˜}{\text{E}}}^{1-\iota_{j}}),\iota \in \varsigma \}$*. Then* ${\left(Q_{st}^{\upsilon \mu \omega }\right)}_{⋎}$ *is the coarsest* stratified svnsq-proximity *on* ¥ *which is finer than* $Q_{⋎}^{\upsilon \mu \omega }$*.*

Proof We will prove ($Q_{5}$) only; Conditions ($Q_{1}$) to ($Q_{4}$) are similar to proving Theorem 5.

($Q_{5}$) Presume there exists ${£}_{\sigma },\hbar_{\alpha }\in \overset{˜}{\left(¥,⋎\right)}$, e∈⋎ such that

$${\left(Q_{st}^{\upsilon }\right)}_{e}({£}_{\sigma },\hbar_{\alpha })≱\underset{g_{{}_{C}}\in \overset{˜}{\left(¥,⋎\right)}}{⋀}\left[{\left(Q_{st}^{\upsilon }\right)}_{e}({£}_{\sigma },g_{{}_{C}})\vee {\left(Q_{st}^{\upsilon }\right)}_{e}(g_{{}_{C}}^{c},\hbar_{\alpha })\right],$$

$${\left(Q_{st}^{\mu }\right)}_{e}({£}_{\sigma },\hbar_{\alpha })≰\underset{g_{{}_{C}}\in \overset{˜}{\left(¥,⋎\right)}}{⋁}\left[{\left(Q_{st}^{\mu }\right)}_{e}({£}_{\sigma },g_{{}_{C}})\wedge {\left(Q_{st}^{\mu }\right)}_{e}(g_{{}_{C}}^{c},\hbar_{\alpha })\right],$$

$${\left(Q_{st}^{\omega }\right)}_{e}({£}_{\sigma },\hbar_{\alpha })≰\underset{g_{{}_{C}}\in \overset{˜}{\left(¥,⋎\right)}}{⋁}\left[{\left(Q_{st}^{\omega }\right)}_{e}({£}_{\sigma },g_{{}_{C}})\wedge {\left(Q_{st}^{\omega }\right)}_{e}(g_{{}_{C}}^{c},\hbar_{\alpha })\right],$$

then there exists $r\in \varsigma_{0}$ such that

$${\left(Q_{st}^{\upsilon }\right)}_{e}({£}_{\sigma },\hbar_{\alpha })<r\leq \underset{g_{{}_{C}}\in \overset{˜}{\left(¥,⋎\right)}}{⋀}\left[{\left(Q_{st}^{\upsilon }\right)}_{e}({£}_{\sigma },g_{{}_{C}})\vee {\left(Q_{st}^{\upsilon }\right)}_{e}(g_{{}_{C}}^{c},\hbar_{\alpha })\right],$$

(12) $${\left(Q_{st}^{\mu }\right)}_{e}({£}_{\sigma },\hbar_{\alpha })>1-r\geq \underset{g_{{}_{C}}\in \overset{˜}{\left(¥,⋎\right)}}{⋁}\left[{\left(Q_{st}^{\mu }\right)}_{e}({£}_{\sigma },g_{{}_{C}})\wedge {\left(Q_{st}^{\mu }\right)}_{e}(g_{{}_{C}}^{c},\hbar_{\alpha })\right],$$

$${\left(Q_{st}^{\omega }\right)}_{e}({£}_{\sigma },\hbar_{{}_{B}})>1-r\geq \underset{g_{{}_{C}}\in \overset{˜}{\left(¥,⋎\right)}}{⋁}\left[{\left(Q_{st}^{\omega }\right)}_{e}({£}_{\sigma },g_{{}_{C}})\wedge {\left(Q_{st}^{\omega }\right)}_{e}(g_{{}_{C}}^{c},\hbar_{\alpha })\right].$$

From the concept of ${\left(Q_{st}^{\upsilon \mu \omega }\right)}_{¥}$, there exists a collection $A=\{{\left({£}_{\sigma }\right)}_{j},{\left(\hbar_{\alpha }\right)}_{j},{\overset{˜}{⋎}}^{\iota_{j}}|j\in J\}$ $\in D({£}_{\sigma },\hbar_{\alpha })\}$ such that

$${\left(Q_{st}^{\upsilon }\right)}_{e}({£}_{\sigma },\hbar_{\alpha })=⋀\left[\underset{\left[{\left({£}_{\sigma }\right)}_{i},{\left(\hbar_{\alpha }\right)}_{i},{\overset{˜}{⋎}}^{\iota_{i}}\right]\in A}{⋁}Q_{e}^{\upsilon }({\left({£}_{\sigma }\right)}_{i},{\left(\hbar_{\alpha }\right)}_{i})\right]\geq \underset{g_{{}_{C}}\in \overset{˜}{\left(¥,⋎\right)}}{⋀}\left[\underset{\left({\left({£}_{\sigma }\right)}_{i},{\left(\hbar_{\alpha }\right)}_{i},{\overset{˜}{⋎}}^{\iota_{i}}\right)\in A}{⋁}\left[{\left(Q_{st}^{\upsilon }\right)}_{e}({\left({£}_{\sigma }\right)}_{i},{\left(g_{{}_{C}}\right)}_{i})\vee {\left(Q_{st}^{\upsilon }\right)}_{e}({\left(g_{{}_{C}}^{c}\right)}_{i},{\left(\hbar_{\alpha }\right)}_{i})\right]\right]\geq r,$$

$${\left(Q_{st}^{\mu }\right)}_{e}({£}_{\sigma },\hbar_{\alpha })=⋁\left[\underset{\left({\left({£}_{\alpha }\right)}_{i},{\left(\hbar_{\alpha }\right)}_{i},{\overset{˜}{⋎}}^{\iota_{i}}\right)\in A}{⋀}Q_{e}^{\mu }({\left({£}_{\sigma }\right)}_{i},{\left(\hbar_{\alpha }\right)}_{i})\right]\leq \underset{g_{{}_{C}}\in \overset{˜}{\left(¥,⋎\right)}}{⋁}\left[\underset{\left({\left({£}_{\sigma }\right)}_{i},{\left(\hbar_{\alpha }\right)}_{i},{\overset{˜}{\text{E}}}^{\iota_{i}}\right)\in A}{⋀}\left[{\left(Q_{st}^{\mu }\right)}_{e}({\left({£}_{\sigma }\right)}_{i},{\left(g_{{}_{C}}\right)}_{i})\wedge {\left(Q_{st}^{\mu }\right)}_{e}({\left(g_{{}_{C}}^{c}\right)}_{i},{\left(\hbar_{\alpha }\right)}_{i})\right]\right]\leq 1-r,$$

$${\left(Q_{st}^{\omega }\right)}_{e}({£}_{\sigma },\hbar_{\alpha })=⋁\left[\underset{\left({\left({£}_{\sigma }\right)}_{i},{\left(\hbar_{\alpha }\right)}_{i},{\overset{˜}{⋎}}^{\iota_{i}}\right)\in A}{⋀}Q_{e}^{\omega }({\left({£}_{\sigma }\right)}_{i},{\left(\hbar_{\alpha }\right)}_{i})\right]\leq \underset{g_{{}_{C}}\in \overset{˜}{\left(¥,⋎\right)}}{⋁}\left[\underset{\left({\left({£}_{\sigma }\right)}_{i},{\left(\hbar_{\alpha }\right)}_{i},{\overset{˜}{⋎}}^{\iota_{i}}\right)\in A}{⋀}\left[{\left(Q_{st}^{\omega }\right)}_{e}({\left({£}_{\sigma }\right)}_{i},{\left(g_{{}_{C}}\right)}_{i})\wedge {\left(Q_{st}^{\omega }\right)}_{e}({\left(g_{{}_{C}}^{c}\right)}_{i},{\left(\hbar_{\alpha }\right)}_{i})\right]\right]\leq 1-r.$$

A contradiction for equation (12). □

Theorem 11 *Let*$\left(¥,Q_{⋎}^{\upsilon \mu \omega }\right)$*and*$\left(U,{\left(Q_{R}^{\upsilon \mu \omega }\right)}^{⋆}\right)$*be two* svnsq-proximity spaces*. If* $\psi_{{}_{\varphi }}:(¥,Q_{⋎}^{\upsilon \mu \omega })\to \overset{˜}{(U,{\left(Q_{R}^{\upsilon \mu \omega }\right)}^{⋆}})$ *be a* svnsq-proximity continuous*, then* $\psi_{{}_{\varphi }}:(¥,{\left(Q_{⋎}^{\upsilon \mu \omega }\right)}_{st})\to \overset{˜}{(U,{\left(Q_{R}^{\upsilon \mu \omega }\right)}_{st}^{⋆}})$ *is a* svnsq-proximity continuous

Proof Let ${£}_{\sigma },\hbar_{\alpha }\in \overset{˜}{\left(U,R\right)}$ with ${£}_{\sigma }⊑{⊔}_{j\in J}({\left({£}_{\sigma }\right)}_{j}⊓{\overset{˜}{R}}^{\iota_{j}})\;and\;\hbar_{\alpha }⊑{⊓}_{j\in J}({\left(\hbar_{\alpha }\right)}_{j}⊔{\overset{˜}{R}}^{1-\iota_{j}})$. Then $\psi_{{}_{\varphi }}^{-1}({£}_{\sigma })⊑{⊔}_{j\in J}\psi_{{}_{\varphi }}^{-1}({\left({£}_{\sigma }\right)}_{j})⊓{\overset{˜}{⋎}}^{\iota_{j}}$ and $\psi_{{}_{\varphi }}^{-1}(\hbar_{\alpha })⊑{⊓}_{j\in J}\psi_{{}_{\varphi }}^{-1}({\left(\hbar_{\alpha }\right)}_{j})⊔{\overset{˜}{⋎}}^{1-\iota_{j}}$. For each collection $D({£}_{\sigma },\hbar_{\alpha })=\{\{({\left({£}_{\sigma }\right)}_{j},{\left(\hbar_{\alpha }\right)}_{j},{\overset{˜}{R}}^{\iota_{j}})|j\in J,J\;is\;finite)\}|{£}_{\sigma }⊑{⊔}_{j\in J}({\left({£}_{\sigma }\right)}_{j}⊓{\overset{˜}{R}}^{\iota_{j}})\;and\;\hbar_{\alpha }⊑{⊓}_{j\in J}({\left(\hbar_{\alpha }\right)}_{j}⊔{\overset{˜}{R}}^{1-\iota_{j}}),\iota \in \varsigma \}$, and put $A=\{{\left({£}_{\sigma }\right)}_{j},{\left(\hbar_{\alpha }\right)}_{j},{\overset{˜}{R}}^{\iota_{j}}|j\in J\}$, we have

$${\left(Q_{e}^{\upsilon }\right)}_{st}\left(\psi_{{}_{\varphi }}^{-1}({£}_{\sigma }),\psi_{{}_{\varphi }}^{-1}(\hbar_{\alpha })\right)\geq \underset{\left(\psi_{{}_{\varphi }}^{-1}({\left({£}_{\sigma }\right)}_{j_{{}_{l}}}),\psi_{{}_{\varphi }}^{-1}({\left(\hbar_{\alpha }\right)}_{j_{{}_{l}}}),{\overset{˜}{⋎}}^{\iota_{j_{{}_{l}}}}\right)\in \left\{\psi_{{}_{\varphi }}^{-1}({\left({£}_{\sigma }\right)}_{j}),\psi_{{}_{\varphi }}^{-1}({\left(\hbar_{\alpha }\right)}_{j}),{\overset{˜}{⋎}}^{\iota_{j}}|j\in J\right\}}{⋁}\;Q_{e}^{\upsilon }\left(\psi_{{}_{\varphi }}^{-1}({\left({£}_{\sigma }\right)}_{j_{{}_{l}}}),\psi_{{}_{\varphi }}^{-1}({\left(\hbar_{\alpha }\right)}_{j_{{}_{l}}})\right)\geq \underset{\left({\left({£}_{\sigma }\right)}_{j_{{}_{l}}},{\left(\hbar_{\alpha }\right)}_{j_{{}_{l}}}),{\overset{˜}{R}}^{\iota_{j_{{}_{l}}}}\right)\in A}{⋁}{\left(Q_{\varphi (e)}^{\upsilon }\right)}^{⋆}({\left({£}_{\sigma }\right)}_{j_{{}_{l}}},{\left(\hbar_{\alpha }\right)}_{j_{{}_{l}}})\geq \underset{D({£}_{\sigma },\hbar_{\alpha })}{⋀}\left(\underset{\left({\left({£}_{\sigma }\right)}_{j_{{}_{l}}},{\left(\hbar_{\alpha }\right)}_{j_{{}_{l}}}),{\overset{˜}{R}}^{\iota_{j_{{}_{l}}}}\right)\in A}{⋁}{\left(Q_{\varphi (e)}^{\upsilon }\right)}^{⋆}({\left({£}_{\sigma }\right)}_{j_{{}_{l}}},{\left(\hbar_{\alpha }\right)}_{j_{{}_{l}}})\right)={\left(Q_{\varphi (e)}^{\upsilon }\right)}_{st}^{{}^{⋆}}({£}_{\sigma },\hbar_{\alpha })$$

$${\left(Q_{e}^{\mu }\right)}_{st}\left(\psi_{{}_{\varphi }}^{-1}({£}_{\sigma }),\psi_{{}_{\varphi }}^{-1}(\hbar_{\alpha })\right)\leq \underset{\left(\psi_{{}_{\varphi }}^{-1}({\left({£}_{\sigma }\right)}_{j_{{}_{l}}}),\psi_{{}_{\varphi }}^{-1}({\left(\hbar_{\alpha }\right)}_{j_{{}_{l}}}),{\overset{˜}{⋎}}^{\iota_{j_{{}_{l}}}}\right)\in \left\{\psi_{{}_{\varphi }}^{-1}({\left({£}_{\sigma }\right)}_{j}),\psi_{{}_{\varphi }}^{-1}({\left(\hbar_{\alpha }\right)}_{j}),{\overset{˜}{⋎}}^{\iota_{j}}|j\in J\right\}}{⋀}\;Q_{e}^{\mu }\left(\psi_{{}_{\varphi }}^{-1}({\left({£}_{\sigma }\right)}_{j_{{}_{l}}}),\psi_{{}_{\varphi }}^{-1}({\left(\hbar_{\alpha }\right)}_{j_{{}_{l}}})\right)\leq \underset{\left({\left({£}_{\sigma }\right)}_{j_{{}_{l}}},{\left(\hbar_{\alpha }\right)}_{j_{{}_{l}}}),{\overset{˜}{R}}^{\iota_{j_{{}_{l}}}}\right)\in A}{⋀}{\left(Q_{\varphi (e)}^{\mu }\right)}^{⋆}({\left({£}_{\sigma }\right)}_{j_{{}_{l}}},{\left(\hbar_{\alpha }\right)}_{j_{{}_{l}}})\leq \underset{D({£}_{\sigma \},\hbar_{\alpha }})}{⋁}\left(\underset{\left({\left({£}_{\sigma }\right)}_{j_{{}_{l}}},{\left(\hbar_{\alpha }\right)}_{j_{{}_{l}}},{\overset{˜}{R}}^{\iota_{j_{{}_{l}}}}\right)\in A}{⋀}{\left(Q_{\varphi (e)}^{\mu }\right)}^{⋆}({\left({£}_{\sigma }\right)}_{j_{{}_{l}}},{\left(\hbar_{\alpha }\right)}_{j_{{}_{l}}})\right)={\left(Q_{\varphi (e)}^{\mu }\right)}_{st}^{{}^{⋆}}({£}_{\sigma },\hbar_{\alpha })$$

$${\left(Q_{e}^{\omega }\right)}_{st}\left(\psi_{{}_{\varphi }}^{-1}({£}_{\sigma }),\psi_{{}_{\varphi }}^{-1}(\hbar_{\alpha })\right)\leq \underset{\left(\psi_{{}_{\varphi }}^{-1}({\left({£}_{\sigma }\right)}_{j_{{}_{l}}}),\psi_{{}_{\varphi }}^{-1}({\left(\hbar_{\alpha }\right)}_{j_{{}_{l}}}),{\overset{˜}{⋎}}^{\iota_{j_{{}_{l}}}}\right)\in \left\{\psi_{{}_{\varphi }}^{-1}({\left({£}_{\sigma }\right)}_{j}),\psi_{{}_{\varphi }}^{-1}({\left(\hbar_{\alpha }\right)}_{j}),{\overset{˜}{⋎}}^{\iota_{j}}|j\in J\right\}}{⋀}\;Q_{e}^{\omega }\left(\psi_{{}_{\varphi }}^{-1}({\left({£}_{\sigma }\right)}_{j_{{}_{l}}}),\psi_{{}_{\varphi }}^{-1}({\left(\hbar_{\alpha }\right)}_{j_{{}_{l}}})\right)\leq \underset{\left({\left({£}_{\sigma }\right)}_{j_{{}_{l}}},{\left(\hbar_{\alpha }\right)}_{j_{{}_{l}}}),{\overset{˜}{R}}^{\iota_{j_{{}_{l}}}}\right)\in A}{⋀}{\left(Q_{\varphi (e)}^{\omega }\right)}^{⋆}({\left({£}_{\sigma }\right)}_{j_{{}_{l}}},{\left(\hbar_{\alpha }\right)}_{j_{{}_{l}}})\leq \underset{D({£}_{\sigma \},\hbar_{\alpha }})}{⋁}\left(\underset{\left({\left({£}_{\sigma }\right)}_{j_{{}_{l}}},{\left(\hbar_{\alpha }\right)}_{j_{{}_{l}}},{\overset{˜}{R}}^{\iota_{j_{{}_{l}}}}\right)\in A}{⋀}{\left(Q_{\varphi (e)}^{\omega }\right)}^{⋆}({\left({£}_{\sigma }\right)}_{j_{{}_{l}}},{\left(\hbar_{\alpha }\right)}_{j_{{}_{l}}})\right)={\left(Q_{\varphi (e)}^{\omega }\right)}_{st}^{{}^{⋆}}({£}_{\sigma },\hbar_{\alpha })$$

By Definition 5, we have $\psi_{{}_{\varphi }}:(¥,{\left(Q_{⋎}^{\upsilon \mu \omega }\right)}_{st})\to \overset{˜}{(U,{\left(Q_{R}^{\upsilon \mu \omega }\right)}_{st}^{⋆}})$ is a *svnsq-proximity continuous*. □

Theorem 12 *Let*$\left(¥,Q_{⋎}^{\upsilon \mu \omega }\right)$*be* svnsq-proximity space*. Then, for* ${£}_{\sigma },\hbar_{{}_{B}}\in \overset{˜}{\left(¥,⋎\right)}$*,* e∈⋎*.*

*(i)*$H_{Q}^{\upsilon },H_{Q}^{\mu },H_{Q}^{\omega }:⋎\to \varsigma^{\overset{˜}{\left(¥,⋎\right)}\times \overset{˜}{\left(¥,⋎\right)}}$*defined by,*${\left(H_{Q}^{\upsilon }\right)}_{e}({£}_{\sigma },\hbar_{\alpha })={\left[Q_{e}^{\upsilon }({£}_{\sigma },\hbar_{\alpha }^{c})\right]}^{c}$*,*${\left(H_{Q}^{\mu }\right)}_{e}({£}_{\sigma },\hbar_{\alpha })={\left[Q_{e}^{\mu }({£}_{{}_{\sigma }},\hbar_{\alpha }^{c})\right]}^{c}$*and*${\left(H_{Q}^{\omega }\right)}_{e}({£}_{\sigma },\hbar_{\alpha })={\left[Q_{e}^{\omega }({£}_{\sigma },\hbar_{\alpha }^{c})\right]}^{c}$*is* symmetrical svn-topogenous order *on* ¥*.*

*(ii)* ${\left(H_{Q_{st}}^{\upsilon \mu \omega }\right)}_{⋎}={\left[{\left(H_{Q}^{\upsilon \mu \omega }\right)}_{\text{st}}\right]}_{⋎}$ *.*

Proof (i) Straightforward.

(ii) Since ${\left(H_{Q_{st}}^{\upsilon }\right)}_{e}({\overset{˜}{⋎}}^{\iota },{\overset{˜}{⋎}}^{\iota })={\left[{\left(Q_{st}^{\upsilon }\right)}_{e}({\overset{˜}{⋎}}^{\iota },{\overset{˜}{⋎}}^{1-\iota })\right]}^{c}=1$, ${\left(H_{Q_{st}}^{\mu }\right)}_{e}({\overset{˜}{⋎}}^{\iota },{\overset{˜}{⋎}}^{\iota })={\left[{\left(Q_{st}^{\mu }\right)}_{e}({\overset{˜}{⋎}}^{\iota },{\overset{˜}{⋎}}^{1-\iota })\right]}^{c}=0$ and ${\left(H_{Q_{st}}^{\omega }\right)}_{e}({\overset{˜}{⋎}}^{\iota },{\overset{˜}{⋎}}^{\iota })={\left[{\left(Q_{st}^{\omega }\right)}_{e}({\overset{˜}{⋎}}^{\iota },{\overset{˜}{⋎}}^{1-\iota })\right]}^{c}=0$, for every ι∈ς, we obtain ${\left[{\left(H_{Q}^{\upsilon \mu \omega }\right)}_{st}\right]}_{⋎}$ is stratified which is finer than ${\left(H_{Q}^{\upsilon \mu \omega }\right)}_{⋎}$. Hence, ${\left(H_{Q_{st}}^{\upsilon \mu \omega }\right)}_{⋎}⊒{\left[{\left(H_{Q}^{\upsilon \mu \omega }\right)}_{st}\right]}_{⋎}$.

Conversely, assume there exist ${£}_{{}_{A}},\hbar_{{}_{\alpha }}\in \overset{˜}{\left(¥,⋎\right)}$, e∈⋎ such that,

$${\left(H_{Q_{st}}^{\upsilon }\right)}_{e}({£}_{\sigma },\hbar_{\alpha })={\left[{\left(Q_{st}^{\upsilon }\right)}_{e}({£}_{\sigma },\hbar_{\alpha }^{c})\right]}^{c}≰{\left[{\left(H_{Q}^{\upsilon }\right)}_{st}\right]}_{e}({£}_{\sigma },\hbar_{\alpha }),$$

$${\left(H_{Q_{st}}^{\mu }\right)}_{e}({£}_{\sigma },\hbar_{\alpha })={\left[{\left(Q_{st}^{\mu }\right)}_{e}({£}_{\sigma },\hbar_{\alpha }^{c})\right]}^{c}≱{\left[{\left(H_{Q}^{\mu }\right)}_{st}\right]}_{e}({£}_{\sigma },\hbar_{\alpha }),$$

$${\left(H_{Q_{st}}^{\omega }\right)}_{e}({£}_{\sigma },\hbar_{\alpha })={\left[{\left(Q_{st}^{\omega }\right)}_{e}({£}_{\sigma },\hbar_{\alpha }^{c})\right]}^{c}≱{\left[{\left(H_{Q}^{\omega }\right)}_{st}\right]}_{e}({£}_{\sigma },\hbar_{\alpha }).$$

By the concept of ${\left(Q_{st}^{\upsilon \mu \omega }\right)}_{\text{E}}$, there exists a collection $A=\{{\left({£}_{\sigma }\right)}_{j},{\left(\hbar_{\alpha }\right)}_{j},{\overset{˜}{⋎}}^{\iota_{j}}|j\in J\}$ $\in D({£}_{\sigma },\hbar_{\alpha }^{c})\}$ such that

$${\left[{\left(H_{Q}^{\upsilon }\right)}_{st}\right]}_{e}({£}_{\sigma },\hbar_{\alpha })≱\underset{\left({\left({£}_{\sigma }\right)}_{i},{\left(\hbar_{\alpha }\right)}_{i},{\overset{˜}{⋎}}^{\iota_{i}}\right)\in A}{⋀}{\left[Q_{e}^{\upsilon }({\left({£}_{\sigma }\right)}_{i},{\left(\hbar_{\alpha }\right)}_{i})\right]}^{c},$$

(13) $${\left[{\left(H_{Q}^{\mu }\right)}_{st}\right]}_{e}({£}_{\sigma },\hbar_{\alpha })≰\underset{\left({\left({£}_{\sigma }\right)}_{i},{\left(\hbar_{\alpha }\right)}_{i},{\overset{˜}{⋎}}^{\iota_{i}}\right)\in A}{⋁}{\left[Q_{e}^{\mu }({\left({£}_{\sigma }\right)}_{i},{\left(\hbar_{\alpha }\right)}_{i})\right]}^{c},$$

$${\left[{\left(H_{Q}^{\omega }\right)}_{st}\right]}_{e}({£}_{\sigma },\hbar_{\alpha })≰\underset{\left({\left({£}_{\sigma }\right)}_{i},{\left(\hbar_{\alpha }\right)}_{i},{\overset{˜}{⋎}}^{\iota_{i}}\right)\in A}{⋁}{\left[Q_{e}^{\omega }({\left({£}_{\sigma }\right)}_{i},{\left(\hbar_{\alpha }\right)}_{i})\right]}^{c}.$$

On another side, $M=\left\{{\left({£}_{\sigma }\right)}_{j},{\left(\hbar_{\alpha }^{c}\right)}_{j},{\overset{˜}{⋎}}^{\iota_{j}}|j\in J\right\}\in N({£}_{\sigma },\hbar_{\alpha })$ we have

$${\left[{\left(H_{Q}^{\upsilon }\right)}_{st}\right]}_{e}({£}_{\sigma },\hbar_{\alpha })\geq \underset{\left({\left({£}_{\sigma }\right)}_{i},{\left(\hbar_{\alpha }^{c}\right)}_{i},{\overset{˜}{⋎}}^{\iota_{i}}\right)\in M}{⋀}{\left(H_{Q}^{\upsilon }\right)}_{{}_{e}}({\left({£}_{\sigma }\right)}_{i},{\left(\hbar_{\alpha }^{c}\right)}_{i})\geq \underset{\left({\left({£}_{\sigma }\right)}_{i},{\left(\hbar_{\alpha }^{c}\right)}_{i},{\overset{˜}{⋎}}^{\iota_{i}}\right)\in M}{⋀}{\left[Q_{e}^{\upsilon }({\left({£}_{\sigma }\right)}_{i},{\left(\hbar_{\alpha }\right)}_{i})\right]}^{c},$$

$${\left[{\left(H_{Q}^{\mu }\right)}_{st}\right]}_{e}({£}_{\sigma },\hbar_{\alpha })\leq \underset{\left({\left({£}_{\sigma }\right)}_{i},{\left(\hbar_{\alpha }^{c}\right)}_{i},{\overset{˜}{⋎}}^{\iota_{i}}\right)\in M}{⋁}{\left(H_{Q}^{\mu }\right)}_{{}_{e}}({\left({£}_{\sigma }\right)}_{i},{\left(\hbar_{\alpha }^{c}\right)}_{i})\leq \underset{\left({\left({£}_{\sigma }\right)}_{i},{\left(\hbar_{\alpha }^{c}\right)}_{i},{\overset{˜}{⋎}}^{\iota_{i}}\right)\in M}{⋁}{\left[Q_{e}^{\mu }({\left({£}_{\sigma }\right)}_{i},{\left(\hbar_{\alpha }\right)}_{i})\right]}^{c},$$

$${\left[{\left(H_{Q}^{\omega }\right)}_{st}\right]}_{e}({£}_{\sigma },\hbar_{\alpha })\leq \underset{\left({\left({£}_{{}_{A}}\right)}_{i},{\left(\hbar_{\alpha }^{c}\right)}_{i},{\overset{˜}{⋎}}^{\iota_{i}}\right)\in M}{⋁}{\left(H_{Q}^{\omega }\right)}_{{}_{e}}({\left({£}_{\sigma }\right)}_{i},{\left(\hbar_{\alpha }^{c}\right)}_{i})\leq \underset{\left({\left({£}_{\sigma }\right)}_{i},{\left(\hbar_{\alpha }^{c}\right)}_{i},{\overset{˜}{\text{E}}}^{\iota_{i}}\right)\in M}{⋁}{\left[Q_{e}^{\omega }({\left({£}_{\sigma }\right)}_{i},{\left(\hbar_{\alpha }\right)}_{i})\right]}^{c},$$

which is a contradiction of equations (13). Therefore, ${\left(H_{Q_{st}}^{\upsilon \mu \omega }\right)}_{⋎}={\left[{\left(H_{Q}^{\upsilon \mu \omega }\right)}_{st}\right]}_{⋎}$. □

Theorem 13 *Let*$H_{⋎}^{\upsilon \mu \omega }$*be* symmetrical svn-topogenous order *on* ⋎*. Then, for all* ${£}_{\sigma },\hbar_{\alpha }\in \overset{˜}{\left(¥,⋎\right)}$*,* e∈⋎*.*

*(i)* $Q_{H}^{\upsilon },Q_{H}^{\mu },Q_{H}^{\omega }:⋎\to \varsigma^{\overset{˜}{\left(¥,⋎\right)}\times \overset{˜}{\left(¥,⋎\right)}}$ *defined by,* ${\left(Q_{H}^{\upsilon }\right)}_{e}({£}_{\sigma },\hbar_{\alpha })={\left[H_{e}^{\upsilon }({£}_{\sigma },\hbar_{\alpha }^{c})\right]}^{c}$ *,*

${\left(Q_{H}^{\mu }\right)}_{e}({£}_{\sigma },\hbar_{\alpha })={\left[H_{e}^{\mu }({£}_{\sigma },\hbar_{\alpha }^{c})\right]}^{c}$*and*${\left(Q_{H}^{\omega }\right)}_{e}({£}_{\sigma },\hbar_{\alpha })={\left[H_{e}^{\omega }({£}_{\sigma },\hbar_{{}_{B}}^{c})\right]}^{c}$*is* svnq-proximity *on* ¥*.*

*(ii)* ${\left(Q_{H_{st}}^{\upsilon \mu \omega }\right)}_{⋎}={\left[{\left(Q_{H}^{\upsilon \mu \omega }\right)}_{st}\right]}_{⋎}$ *.*

Proof (i) Obvious.

(ii) Since ${\left(Q_{H_{st}}^{\upsilon }\right)}_{e}({\overset{˜}{⋎}}^{\iota },{\overset{˜}{⋎}}^{\iota })=1-{\left(H_{st}^{\upsilon }\right)}_{e}({\overset{˜}{⋎}}^{\iota },{\overset{˜}{⋎}}^{1-\iota })=1-0=1$, ${\left(Q_{H_{st}}^{\mu }\right)}_{e}({\overset{˜}{⋎}}^{\iota },{\overset{˜}{⋎}}^{\iota })=1-{\left(H_{st}^{\mu }\right)}_{e}({\overset{˜}{⋎}}^{\iota },{\overset{˜}{⋎}}^{1-\iota })]=1-1=0$ and ${\left(Q_{H_{st}}^{\omega }\right)}_{e}({\overset{˜}{⋎}}^{\iota },{\overset{˜}{⋎}}^{\iota })=1-{\left(H_{st}^{\omega }\right)}_{e}({\overset{˜}{⋎}}^{\iota },{\overset{˜}{⋎}}^{1-\iota })=1-1=0$, for every ι∈ς, we have ${\left[{\left(Q_{H}^{\upsilon \mu \omega }\right)}_{st}\right]}_{⋎}$ is stratified which is finer than ${\left(Q_{H}^{\upsilon \mu \omega }\right)}_{⋎}$. Hence, ${\left(Q_{H_{st}}^{\upsilon \mu \omega }\right)}_{⋎}⊒{\left[{\left(Q_{H}^{\upsilon \mu \omega }\right)}_{st}\right]}_{⋎}$.

Conversely, suppose that there exist ${£}_{{}_{A}},\hbar_{{}_{\alpha }}\in \overset{˜}{\left(¥,⋎\right)}$, e∈⋎ such that,

$${\left(Q_{H_{st}}^{\upsilon }\right)}_{e}({£}_{\sigma },\hbar_{\alpha })=1-{\left(H_{st}^{\upsilon }\right)}_{e}({£}_{\sigma },\hbar_{\alpha }^{c})≰{\left[{\left(Q_{H}^{\upsilon }\right)}_{st}\right]}_{e}({£}_{\sigma },\hbar_{\alpha }),$$

$${\left(Q_{H_{st}}^{\mu }\right)}_{e}({£}_{\sigma },\hbar_{\alpha })=1-{\left(H_{st}^{\mu }\right)}_{e}({£}_{\sigma },\hbar_{\alpha }^{c})≱{\left[{\left(Q_{H}^{\mu }\right)}_{st}\right]}_{e}({£}_{\sigma },\hbar_{\alpha }),$$

$${\left(Q_{H_{st}}^{\omega }\right)}_{e}({£}_{\sigma },\hbar_{\alpha })=1-{\left(H_{st}^{\omega }\right)}_{e}({£}_{\sigma },\hbar_{\alpha }^{c})≱{\left[{\left(Q_{H}^{\omega }\right)}_{st}\right]}_{e}({£}_{\sigma },\hbar_{\alpha }).$$

By the concept of ${\left(H_{st}^{\upsilon \mu \omega }\right)}_{\text{E}}$, there exists a collection $\left\{{\left({£}_{\sigma }\right)}_{j},{\left(\hbar_{\alpha }\right)}_{j},{\overset{˜}{⋎}}^{\iota_{j}}|j\in J\right\}=A\in N({£}_{\sigma },\hbar_{\alpha }^{c})$ such that

$${\left[{\left(Q_{H}^{\upsilon }\right)}_{st}\right]}_{e}({£}_{\sigma },\hbar_{\alpha })≱\underset{\left({\left({£}_{\sigma }\right)}_{i},{\left(\hbar_{\alpha }\right)}_{i},{\overset{˜}{⋎}}^{\iota_{i}}\right)\in A}{⋀}1-H_{e}^{\upsilon }({\left({£}_{\sigma }\right)}_{i},{\left(\hbar_{\alpha }\right)}_{i}),$$

(14) $${\left[{\left(Q_{H}^{\mu }\right)}_{st}\right]}_{e}({£}_{\sigma },\hbar_{\alpha })≰\underset{\left({\left({£}_{\sigma }\right)}_{i},{\left(\hbar_{\alpha }\right)}_{i},{\overset{˜}{⋎}}^{\iota_{i}}\right)\in A}{⋁}1-H_{e}^{\mu }({\left({£}_{\sigma }\right)}_{i},{\left(\hbar_{\alpha }\right)}_{i}),$$

$${\left[{\left(Q_{H}^{\omega }\right)}_{st}\right]}_{e}({£}_{\sigma },\hbar_{\alpha })≰\underset{\left({\left({£}_{\sigma }\right)}_{i},{\left(\hbar_{\alpha }\right)}_{i},{\overset{˜}{⋎}}^{\iota_{i}}\right)\in A}{⋁}1-H_{e}^{\omega }({\left({£}_{\sigma }\right)}_{i},{\left(\hbar_{\alpha }\right)}_{i}).$$

On the other hand, $P=\left\{{\left({£}_{\sigma }\right)}_{j},{\left(\hbar_{\alpha }^{c}\right)}_{j},{\overset{˜}{⋎}}^{\iota_{j}}|j\in J\right\}\in D({£}_{\sigma },\hbar_{\alpha })$ we have

$${\left[{\left(Q_{H}^{\upsilon }\right)}_{st}\right]}_{e}({£}_{\sigma },\hbar_{\alpha })\geq \underset{\left({\left({£}_{\sigma }\right)}_{i},{\left(\hbar_{\alpha }^{c}\right)}_{i},{\overset{˜}{⋎}}^{\iota_{i}}\right)\in P}{⋀}{\left(Q_{H}^{\upsilon }\right)}_{{}_{e}}({\left({£}_{\sigma }\right)}_{i},{\left(\hbar_{\alpha }^{c}\right)}_{i})\geq \underset{\left({\left({£}_{\sigma }\right)}_{i},{\left(\hbar_{\alpha }^{c}\right)}_{i},{\overset{˜}{⋎}}^{\iota_{i}}\right)\in P}{⋀}1-H_{e}^{\upsilon }({\left({£}_{\sigma }\right)}_{i},{\left(\hbar_{\alpha }\right)}_{i}),$$

$${\left[{\left(Q_{H}^{\mu }\right)}_{st}\right]}_{e}({£}_{\sigma },\hbar_{\alpha })\leq \underset{\left({\left({£}_{\sigma }\right)}_{i},{\left(\hbar_{\alpha }^{c}\right)}_{i},{\overset{˜}{⋎}}^{\iota_{i}}\right)\in P}{⋁}{\left(Q_{H}^{\mu }\right)}_{{}_{e}}({\left({£}_{\sigma }\right)}_{i},{\left(\hbar_{\alpha }^{c}\right)}_{i})\leq \underset{\left({\left({£}_{\sigma }\right)}_{i},{\left(\hbar_{\alpha }^{c}\right)}_{i},{\overset{˜}{⋎}}^{\iota_{i}}\right)\in P}{⋁}1-H_{e}^{\mu }({\left({£}_{\sigma }\right)}_{i},{\left(\hbar_{\alpha }\right)}_{i}),$$

$${\left[{\left(Q_{H}^{\omega }\right)}_{st}\right]}_{e}({£}_{\sigma },\hbar_{\alpha })\leq \underset{\left({\left({£}_{{}_{A}}\right)}_{i},{\left(\hbar_{\alpha }^{c}\right)}_{i},{\overset{˜}{⋎}}^{\iota_{i}}\right)\in P}{⋁}{\left(Q_{H}^{\omega }\right)}_{{}_{e}}({\left({£}_{\sigma }\right)}_{i},{\left(\hbar_{\alpha }^{c}\right)}_{i})\leq \underset{\left({\left({£}_{\sigma }\right)}_{i},{\left(\hbar_{\alpha }^{c}\right)}_{i},{\overset{˜}{\text{E}}}^{\iota_{i}}\right)\in P}{⋁}1-H_{e}^{\omega }({\left({£}_{\sigma }\right)}_{i},{\left(\hbar_{\alpha }\right)}_{i}),$$

which is a contradiction of equations (14). Hence, ${\left(Q_{H_{st}}^{\upsilon \mu \omega }\right)}_{⋎}={\left[{\left(Q_{H}^{\upsilon \mu \omega }\right)}_{st}\right]}_{⋎}$. ${\left(H_{Q_{st}}^{\upsilon \mu \omega }\right)}_{⋎}={\left[{\left(H_{Q}^{\upsilon \mu \omega }\right)}_{st}\right]}_{⋎}$. □

## 5 Conclusions

Theoretically, we have advanced a set of overarching concepts derived from the principles outlined in previous works [48], [49]. These include the stratified single-valued neutrosophic soft quasi proximity, stratified single-valued neutrosophic soft topogenous order, stratified single-valued neutrosophic soft filter, and stratified single-valued neutrosophic soft quasi uniformity, along with an exploration of their respective characteristics. Additionally, we have studied the interconnectedness between these single-valued neutrosophic soft topological constructs and their stratifications.

Regarding further research, we intend to evaluate the correlations between our findings and advancements in neural networks [50], [51], [52], [53], [54], multidimensional systems and signal processing [55], [56], [57], as well as optimization algorithms [58] and global optimization [59]. Finally, we present a practical application of these concepts in solving decision-making problems. This inventive expansion has the potential to greatly expand existing theoretical frameworks for managing indeterminacy, while also opening up new paths for application and research.

**For forthcoming papers**.

The theory can be extended in the next normal methods,

1-Basic concepts can be studied of neutrosophic metric topological spaces using the notion of single-valued neutrosophic soft quasi-uniform present in this article;

2-Examine the connected, separation axioms and soft closure spaces in the context of neutrosophic soft quasi-uniform.

## CRediT authorship contribution statement

**Fahad Alsharari:** Writing – review & editing, Writing – original draft, Funding acquisition, Conceptualization. **Yaser Saber:** Writing – review & editing. **Hanan Alohali:** Writing – review & editing, Writing – original draft. **Mesfer H. Alqahtani:** Writing – original draft, Supervision, Resources, Funding acquisition. **Mubarak Ebodey:** Writing – review & editing. **Tawfik Elmasry:** Writing – review & editing. **Jafar Alsharif:** Writing – review & editing. **Amal F. Soliman:** Writing – review & editing, Data curation. **Florentin Smarandache:** Writing – review & editing. **Fahad Sikander:** Funding acquisition, Formal analysis.

## Declaration of Competing Interest

The authors declare the following financial interests/personal relationships which may be considered as potential competing interests:

Yaser Saber reports financial support was provided by Majmaah University.

## Data Availability

The article outlines the research that used no data.
